# Selected Abstracts from the 2018 NCRI Cancer Conference of National Cancer Research Institute

**DOI:** 10.1038/s41416-018-0299-z

**Published:** 2018-11-08

## Abstract

**Scottish Event Campus, Glasgow, Sunday 4 – Tuesday 6 November 2018**

Attendees of the 2018 NCRI Cancer Conference will be able to contact corresponding authors through the Conference App and e-poster platform at the Conference.

W: https://conference.ncri.org.uk: E: conference@ncri.org.uk

Organised by the National Cancer Research Institute, which is a partnership of 19 cancer research funders, the NCRI Cancer Conference provides a platform for researchers, clinicians, people affected by cancer and industry representatives to come together to discuss, present and showcase high-quality research.

**Sponsorship Statement:** Publication of this supplement was sponsored by Roche. All content was reviewed and approved by the NCRI Scientific Committee, which held full responsibility for the abstract selection.

## 2018 NCRI Cancer Conference Scientific Committee

### 2018 Joint Chairs

**Margaret Frame**, Science Director, CRUK Edinburgh Centre, and Director, MRC Institute of Genetics and Molecular Medicine (IGMM), University of Edinburgh, UK

**Owen Sansom**, Director, Cancer Research UK Beatson Institute, UK

### Scientific Committee Members

**Sue Bailey**, Strategic partnership and Early Asset Director, BMS UK and Ireland, UK, Chair of Industry Consultation Group – no involvement in presentation or speaker selection

**Linda Bauld**, Professor of Health Policy and Dean of Research, University of Stirling, CRUK/BUPA Chair in Behavioural Research for Cancer Prevention, Cancer Research UK

**Francesca Buffa**, Associate Professor and Principal Investigator, Computational Biology and Integrative Genomics, University of Oxford, UK

**David Cameron**, Professor of Oncology and Centre Clinical Director, CRUK Edinburgh Centre, The University of Edinburgh, UK

**Jason Carroll**, Research Group Leader, Cancer Research UK Cambridge Institute, UK

**Pippa Corrie**, Consultant and Associate Lecturer in Medical Oncology, Cambridge University Hospitals NHS Foundation Trust, UK

**Lesley Fallowfield**, Director, Sussex Health Outcomes Research & Education in Cancer (SHORE-C), Brighton & Sussex Medical School, University of Sussex, UK

**Richard Gilbertson**, Director of Cambridge Cancer Centre, Li Ka Shing Chair of Oncology, Head of Dept of Oncology, Cambridge Cancer Centre, UK

**Di Gilson**, Chair of Clinical Oncology Professional Learning and Development Subcommittee

**Diana Greenfield**, Macmillan Consultant Nurse, Sheffield Teaching Hospitals NHS Foundation Trust, UK

**Maria Hawkins**, Associate Professor in Clinical Oncology, MRC Clinical Group Leader/Honorary Consultant Clinical Oncologist, CRUK MRC Oxford Institute for Radiation Oncology, University of Oxford, UK

**Richard Houlston**, Team Leader, The Institute of Cancer Research, UK

**David Jayne**, Professor of Surgery, University of Leeds, UK

**Terry Jones**, Professor of Head and Neck Surgery, Cancer Lead for NIHR CRN NWC and Honorary Consultant Otolaryngologist / Head and Neck Surgeon, University of Liverpool and Aintree University Hospitals NHS Foundation Trust, UK

**Katarzyna Leszczynska**, Postdoctoral Researcher, CRUK/MRC Oxford Institute for Radiation Oncology, University of Oxford, UK

**Chris Lord**, Professor of Cancer Genomics, Deputy Director, Breast Cancer Now Toby Robins Research Centre, The Institute of Cancer Research, UK

**John Marshall**, Consumer Representative

**Tim Maughan**, Professor of Clinical Oncology and Deputy Director of the MRC-CRUK Oxford Institute of Radiation Oncology, Department of Oncology, University of Oxford, UK

**Gillies McKenna**, Director, Molecular Resistance to Treatments Group, CRUK/MRC Oxford Institute for Radiation Oncology, UK

**Eric O’Neill**, CRUK/MRC Oxford Institute, University of Oxford, UK

**Liz Patton**, Programme Leader, MRC Human Genetics Unit Reader, CRUK Edinburgh Centre, MRC Institute of Genetics and Molecular Medicine, University of Edinburgh, UK

**Gemma Pearce**, Research Associate, Centre for Advances in Behavioural Science, Coventry University, UK

**Nitzan Rosenfeld**, Senior Group Leader, CRUK-CI, University of Cambridge, UK

**Matt Seymour**, Chair of Gastrointestinal Cancer Medicine & Hon. Consultant Medical Oncologist, Director of NIHR Cancer Research Network (NCRN), Clinical Research Director of National Cancer Research Institute (NCRI), NIHR Clinical Research Network: cancer, Leeds, UK and the National Cancer Research Institute, UK

**Ian Tomlinson**, Director of the Institute of Cancer and Genomic Sciences, University of Birmingham, UK

**Fiona Walter**, Principal Researcher/ Reader in the Primary Care Unit, Department of Public Health & Primary Care, University of Cambridge, UK

**David Weller**, Head of General Practice, Usher Institute of Population Health Sciences & Informatics, University of Edinburgh, UK

**Lynda Wyld**, Professor of Surgical Oncology, Department of Oncology and Metabolism, University of Sheffield, UK

### Advisor

**Fiona Hemsley**, Director of Research Operations, The Institute of Cancer Research, UK

## Cancer control, living with & beyond and cancer outcomes

### **1. Returning to work after cancer in Romania – the patients' views on motivations and barriers**

#### Adela Popa^*1*^

##### ^*1*^*Lucian Blaga University of Sibiu, Romania*

**Background:** A substantial proportion of cancer patients in Europe and Romania are of working age. In Romania, the number of working-age cancer patients is growing, hence returning to work (RTW) after treatment is an option that must be considered. This research aims to explore the cancer survivors’ perspectives on the motivations and barriers which influenced their RTW, in a country where the legislation provides no incentives or support for RTW.

**Method:** Data were collected through semi-structured interviews. Twenty-eight subjects who either returned to work (n = 16) or not (n = 12) were recruited via oncologists and specialized physicians. The interviews were thematically analysed using Nvivo 11 http://www.qsrinternational.com/nvivo/nvivo-products/nvivo-11-for-windows.

**Results:** The findings suggest important differences in the two sub-samples regarding the motivations for RTW and barriers encountered. The most important motivations mentioned by the patients who returned to work were: seeing work as a distraction from the disease, the financial and the social motivations. Part of the patients who did not return to work was intensely motivated to work, yet they could not return to the same working conditions or find another job due to the diagnosis. Part of the patients which returned to work mentioned they encountered no barriers but admitted the RTW was not phased or prepared in any way. The patients who did not return to work mentioned physical barriers, unreceptive attitude or discrimination from the employer and fear of the co-worker’s reaction.

**Conclusion:** Most of the employees in our sample proved a strong motivation to return to work but only a few of them were offered the needed support for returning to work. In the absence of formal and structured support, RTW was determined by the type of work and the type and stage of the disease. The study emphasizes the importance of structured RTW support that will enable employees to re-enter the workforce.

Disclosure: Funded by Romanian National Authority for Scientific Research, CNCS - UEFISCDI

Corresponding author: Adela Popa

### **2. Randomised controlled trial of holistic needs assessment in outpatient oncology**

#### Austyn Snowden^1^, Jenny Young^1^

##### ^*1*^*Edinburgh Napier University*

**Background:** Holistic needs assessment (HNA) is a 48-item checklist designed to help patients articulate their physical, social, psychological, financial and practical concerns during consultation. The study objective was to establish if and how HNA changes consultation dynamics. It was hypothesised that HNA will support greater patient participation, facilitate shared decision-making and increase self-efficacy.

**Method:** Randomised controlled trial. Three outpatient oncology clinics in West of Scotland recruited patients with head and neck or colorectal cancer who had received treatment (2016-ongoing). Control group entailed routine consultation. Intervention group: patients completed HNA before consultation then the clinician used it to guide consultation. All consultations were audiorecorded. Patient participation was determined by MEDICODE: dialogue ratio (DR) and preponderance of initiative (PI) within consultation. Shared decision-making was measured with CollaboRATE; self-efficacy with Lorig’s measure.

**Results:** Fifty-five people were randomised to intervention, 61 to control. More patient concerns were discussed in the intervention group (8.9) than control (6,6) (p = 0.00). Emotional concerns were discussed more frequently (respectively 27,1% and 19,4%, p = 0.01), and clinicians expressed more support (14,4% compared to 10,2%, p=0.08) in intervention group. Discussion was initiated mainly by clinician in both groups (76,4%), and participation tended to monologue in both (54.1%). However, patient initiative was significantly higher in experimental group when discussing symptom control(respectively 40,0% and 24,1%, p=0.03). Dialogue was higher when discussing symptoms (respectively 75,1% and 63,6%, p=0.01). Self-efficacy was higher in the experimental group (mean Lorig: 8.3 v 7.6 p=0.093), as was CollaboRATE (25.1 v 23.4, p=0.121), though neither was statistically significant.

**Conclusion:** These preliminary results showed HNA changed the dynamics of the clinical consultation away from clinician led monologue towards shared decision making. These trends may become statistically significant when the study reaches sufficient power. The presentation will discuss a more nuanced analysis of the impact of HNA on consultation style.

Clinical Trial Register: NCT02274701, https://www.clinicaltrial.gov

Disclosure: Funded by Macmillan Cancer Support UK

Corresponding author: Jenny Young

### **3. Socioeconomic status and the risk of breast cancer among Nigerian women**

#### Samuel O. Azubuike, Richard McNally, Louise Hayes

##### *Institute of Health and Society, Newcastle University*

**Background:** Breast cancer incidence in Nigeria has risen by > 120% since 2000. The mortality rate (25.9 /100 000/year) ranks highest in Africa. Unfortunately, studies investigating breast cancer risk factors in Africa are few, and none of these studies was aimed at investigating the role of socioeconomic status (SES). In Nigeria, though poverty remains conspicuous in the general population, recent reports have suggested that the SES of many women has been improving. This aim of the study was to investigate if there is an association between socioeconomic status and risk of breast cancer in Nigeria.

**Method:** The study was a hospital-based case-control design involving 379 histologically confirmed breast cancer cases and 403 controls. The participants aged ≥20 years were selected across five facilities in northern (Abuja) and southern (Lagos) Nigeria. They were interviewed in-person between October 2016 and May 2017 using a pretested questionnaire. Cases were selected from oncology wards, while controls were from ophthalmology wards. SES was measured based on education, occupation and income. Data were analysed using multivariable logistic regression. SPSS version 23 was used for all analyses.

**Results:** After controlling for the effects of parity, breastfeeding, age at first birth, hormonal contraceptive use, body mass index, alcohol use, age at menarche, abortion, ethnicity, menopausal status, urban-rural difference, and family size; women in the upper categories of family income (OR: 0.4, 95% CI: 0.18, 0.67, ptrend =0.007) and education (OR: 0.29, 95% CI: 0.12, 0.72, ptrend = 0.035) had reduced risk of breast cancer compared to those in lowest categories of income and education respectively.

**Conclusion:** High income and higher educational attainment were protective against breast cancer in Nigeria. Improving SES of Nigerian women through increased access to higher education and income will provide an opportunity for breast cancer prevention. Future studies could consider using community controls to authenticate the findings.

Disclosure: Funded by National Open University of Nigeria

Corresponding author: Samuel O. Azubuike

### **4. Unsupervised machine learning of integrated health and social care data from the Macmillan Improving the Cancer Journey service in Glasgow**

#### Kean Lee Kang^1^, Margaret Greer^2^, James Bown^1^, Janice Preston^2^, Judith Mabelis^2^, Leigh-Anne Hepburn^3^, Miriam Fisher^4^, Ruth Falconer^1^, Sandra McDermott^5^, Stuart Deed^4^

##### ^*1*^Abertay University, ^2^Macmillan Cancer Support, ^3^Glasgow School of Art, ^4^Digital Health and Care Institute, ^5^Glasgow City Council

**Background:** Improving the Cancer Journey (ICJ) was launched in 2014 by Glasgow City Council and Macmillan Cancer Support. As part of routine service, data is collected on ICJ users including demographic and health information, results from holistic needs assessments and quality of life scores as measured by EQ-5D health status. There is also data on the number and type of referrals made and feedback from users on the overall service. By applying artificial intelligence and interactive visualization technologies to this data, we seek to improve service provision and optimize resource allocation.

**Method:** An unsupervised machine-learning algorithm was deployed to cluster the data. The classical k-means algorithm was extended with the k-modes technique for categorical data, and the gap heuristic automatically identified the number of clusters. The resulting clusters are used to summarize complex data sets and produce three-dimensional visualizations of the data landscape. Furthermore, the traits of new ICJ clients are predicted by approximately matching their details to the nearest existing cluster center.

**Results:** Cross-validation showed the model’s effectiveness over a wide range of traits. For example, the model can predict marital status, employment status and housing type with an accuracy between 2.4 to 4.8 times greater than random selection. One of the most interesting preliminary findings is that area deprivation (measured through Scottish Index of Multiple Deprivation-SIMD) is a better predictor of an ICJ client’s needs than primary diagnosis (cancer type).

**Conclusion:** A key strength of this system is its ability to rapidly ingest new data on its own and derive new predictions from those data. This means the model can guide service provision by forecasting demand based on actual or hypothesized data. The aim is to provide intelligent person-centered recommendations. The machine-learning model described here is part of a prototype software tool currently under development for use by the cancer support community.

Disclosure: Funded by Macmillan Cancer Support

Corresponding author: Kean Lee Kang

### **5. Young colorectal cancer patients experience referral delays in primary care leading to emergency diagnoses**

#### Chanpreet Arhi^1^, Paul Ziprin^1^, Alex Bottle^1^, Elaine Burns^1^, Paul Aylin^1^, Ara Darzi^1^

##### ^*1*^*Imperial College London, UK*

**Background:** The incidence of colorectal cancer in young patients is increasing. In this population based case-control study, we hypothesise missed opportunities for diagnosis in primary care are leading to referral delays and emergency diagnoses in young patients.

**Method:** We compared the interval before diagnosis, presenting symptom (red-flag based on NICE guidance 2005 vs non-specific), the odds ratio (OR) of an emergency diagnosis and stage at diagnosis for those under the age of 50 compared with older patients, sourced from the cancer registry with linkage to the Clinical Practice Research Datalink database of primary care records.

**Results:** 7315 patients were included of which 561 (7.7%) were under 50, 1287 (17.6%) 50–59, 2458 (33.6%) 60–69 and 3534 (48.3%) 70–79 years old. Young patients were more likely to present with abdominal pain (21.1%) and were diagnosed with later stage (48.0%) and via an emergency (29.1%) compared with all age groups. They experienced a longer interval between referral to diagnosis by 12.5 days compared with those aged 60–69, reflecting the higher proportion of referrals via the non-urgent pathway (33.3% vs 26.5%). The OR of an emergency diagnosis did not differ with age if a red-flag symptom was noted at presentation but increased significantly for young patients if the symptom was non-specific compared with all age groups (OR 0.62 95% CI 0.49-0.80 for the 60-69 age group).

**Conclusion:** Young patients are more likely to present with symptoms that fall outside the referral guidelines. Referral criteria should be tailored according to the age group to reduce the risk of an emergency diagnosis due to missed opportunities in primary care.

Disclosure: Funded by Imperial College London

Corresponding author: Chanpreet Arhi

### **6. Delays in primary care are associated with a late stage and worse prognosis for colorectal cancer patients: A population based study**

#### Chanpreet Arhi^1^, Elaine Burns^1^, Alex Bottle^1^, George Bouras^1^, Paul Aylin^1^, Paul Ziprin^1^, Ara Darzi^1^

##### ^1^*Imperial College London UK*

**Background:** Delays in referral for colorectal cancer patients may occur if the presenting symptom is falsely attributed to a benign condition. We hypothesis primary care delays are associated with a later stage at diagnosis and worse prognosis.

**Method:** Patients with a non-emergency colon or rectal cancer in the cancer registry with linkage to the Clinical Practice Research Datalink were included. All had a colorectal associated symptom defined as ‘red flag’ (rectal bleeding, abdominal mass, change in bowel habit, diarrhoea, anaemia) or ‘non-specific’ (abdominal pain, weight loss, constipation, other bowel function) and a relevant referral recorded in the year leading up to diagnosis. The primary care interval (presentation to referral), hospital interval (referral to diagnosis) and stage was compared for each symptom individually and after grouping as above. Cox modelling determined the hazard ratios (HR) of death for each symptom compared with rectal bleeding, and whether delays in referral were associated with a worse prognosis.

**Results:** 4527(63.5%) colon and 2603 (36.5%) rectal cancer patients were included. 16.9% and 13.5% presenting with red-flag symptoms respectively experienced a delay of over three months before referral, compared with 35.7% and 42.9% of those with non-specific symptoms. Delays in primary care did not increase the hospital interval. Each symptom was associated with reduced survival compared with rectal bleeding for both cancers. Patients referred after three months who had presented with red-flag symptoms (Colon: HR 1.53 (1.29–1.81) Rectal: HR 1.30 (1.06 – 1.60)), demonstrated a significantly worse prognosis compared with referrals within two weeks with similar symptoms. This was not seen for patients with non-specific symptoms. Delays in referral significantly increased the proportion of late stage cancers.

**Conclusion:** The first presentation to the GP provides a referral opportunity to identify the underlying cancer, which if missed for patients presenting with red-flag symptoms, is associated with a later stage in diagnosis and worse survival.

Disclosure: Funded by Imperial College London

Corresponding author: Chanpreet Arhi

### **7. Embedding patient voice in cancer service improvement: A qualitative study of patients’ experience of the NHS cancer diagnostic care pathway**

#### Sarah Sowden^1^, Anna Haste^1^, Linda Sharp^1^, Mark Lambert^2^, Richard Thomson^1^

##### ^*1*^*Institute of Health and Society, Newcastle University*, ^*2*^*Public Health England, North East Centre*

**Background:** The 62 day NHS cancer waiting times target for urgent GP referrals is frequently not achieved. Patient insight is essential to understanding why this is the case to inform efforts to reduce waiting times. This study aimed to explore patients’ experiences of the NHS upper gastrointestinal (UGI) cancer diagnostic pathway, seek patients’ ideas for service improvement and identify transferrable insights for other areas of cancer care.

**Method:** Qualitative semi-structured interviews were undertaken in 2017 with 20 patients within 6 months of receiving their first treatment for UGI cancer referred through the urgent (2 week) GP route in the North East and Cumbria. Thematic analysis was undertaken.

**Results:** Key themes identified were organisation of care, diagnosis, support and expectations of the NHS. Some patients were offered immediate access to testing, others required specialist approval first. Patients’ were often unclear about the purpose of visits and journey ahead. Patients contrasted the ‘urgency’ indicated by the requirements placed upon them (and therefore often upon family and friends) to attend numerous appointments, at short notice, in close succession, often across multiple hospital sites, with the seemingly long time taken to receive results, and the unsettling time spent waiting and ‘not knowing’ the prognosis. Patients’ were grateful for the NHS and described how staff were time-pressured but working in their best interests. They did not want to be seen as ‘complaining’ and were often reluctant to express concerns or suggest improvements.

**Conclusion:** Current pathways are complex, varied, can be poorly communicated to patients and do not always appear to be patient-centred. Clearer communication is necessary but insufficient without a review of the diagnostic pathway to reduce unwarranted variation and simplify the patients’ journey. Patients’ gratitude for the NHS and stoic acceptance of the status quo should not be seen as justification for no change.

Disclosure: Funded by NHS England Specialised Commissioning (North East England hub)

Corresponding author: Sarah Sowden

### **8. Real-World Treatment Patterns and Clinical Outcomes in Advanced/Metastatic NSCLC Patients in England**

#### Katharina Verleger^1^, Nadine Hertel^2^, Caitlyn Solem^1^, John R. Penrod^2^, Cynthia Macahilig^3^, Linlin Luo^1^, S. Michael Crawford^4^

##### ^*1*^*Pharmerit International*, ^*2*^*Bristol-Myers Squibb*, ^*3*^*Medical Data Analytics*, ^*4*^*Airedale NHS Foundation Trust*

**Background:** To describe treatment patterns and clinical outcomes for squamous (SQ) and non-SQ (NSQ) advanced non-small-cell lung cancer (aNSCLC) patients in Europe, the LENS (Leading the Evaluation of NSQ and SQ NSCLC) retrospective medical chart review was undertaken. This abstract details findings in England.

**Method:** Adult patients (≥18 years) with NSCLC stage IIIb/IV diagnosis between 07/2010-09/2012 were sampled retrospectively from oncology/respiratory medicine practices in England. Patient medical chart data were extracted from diagnosis to most recent visit/death.

**Results:** 267 patients from 14 hospital-based sites had median age 69.0 years, 53.9% were male, 82.0% stage IV, and 35.6% had ECOG PS 1 (0: 16.5%; 2: 16.9%). 146 (54.7%) patients had ≥1 systemic anti-cancer therapy line (SACT), 6 (2.3%) surgery and 106 (39.7%) radiotherapy. NSQ vs SQ patients were significantly more likely to receive first line (1L) (64.2% vs 45.7%) and second line (2L) (22.6% vs 11.7%) SACT (p<0.05). As 1L treatment, 62.5% of NSQ patients received a pemetrexed combination (SQ: 0.0%), 19.3% other platinum-based combinations, and 13.6% gefitinib monotherapy.

Controlling for death, median time to discontinuation was 2.2 and 2.3 months (m) for 1L and 2L, median time from end of 1L to start of 2L was 23.3m. Median overall survival (OS) from date of aNSCLC diagnosis was 9.8/2.6m for patients with/without 1L SACT. Median 1-year OS rate OS from start of 1L was 23.4% for both SQ and NSQ patients. OS was 14.9m (1-year rate: 60.0%) for targeted 1L SACT patients.

**Conclusion:** The majority of patients in this real-world sample of aNSCLC patients managed by medical oncologists received 1L SACT using agents appropriate for their histological type. However, patient prognosis remained generally poor. The rapidly decreasing proportion of patients receiving SACT in each subsequent line and worse OS in patients not receiving 1L suggest that access to more effective therapies is needed in England.

Disclosure: Funded by Bristol-Myers Squibb

Corresponding author: S. Michael Crawford

### **9. Oncology to oncogeriatrics – improving the skill base of trainees**

#### Cressida Lorimer^1^, Sally Appleyard^1^, Tom Levett^2^

##### ^*1*^*Brighton and Sussex Medical School*, ^*2*^*Royal Sussex County Hospital*

**Background:** The ageing population is providing new challenges for oncologists. Oncology trainees are underequipped to manage geriatric oncology patients with a recent survey showing 66% never receive any formal geriatric oncology trainingGeriatric experience prior to oncology training is mainly limited to inpatient settings, whereas the majority of oncology workload is outpatient. The Rapid Access Clinic for Older Persons (RACOP) is a potential environment in which oncology trainees could experience geriatric assessment techniques.

This project explores the quantity and quality of geriatric oncology training available within the RACOP clinic with a view to implementing a novel training opportunity.

**Method:** Clinic lists for 3 months of RACOP clinics extracted. Notes from patients who had previous, current or new cancer diagnoses made during the clinic were examined. 2 oncology trainees attended RACOP clinics and provided structured reflection on the potential training value.

**Results:** 220 patients were identified in total. 30% had a previous, existing or new cancer diagnosis made. Of the 30 patients (14%) that had a new cancer diagnosis made from their appointment only 2 received active treatment from oncologists due to either overwhelming comorbidities or patient choice. Approximately 75 other geriatric conditions were managed during this time period. Trainees are currently attending RACOP clinic and feedback will be available by the time of the NCRI conference.

**Conclusion:** Geriatric oncology services are being developed in a small number of centres, usually run by geriatricians. The growing numbers of older people living with cancer is likely to outstrip the supply of geriatricians if this model were to be implemented nationally. There is a drive to empower non-geriatricians to perform basic geriatric assessments.

The RACOP clinic provides an excellent resource of an unselected geriatric population and new geriatric oncology patients. This experience can up skill clinical oncology trainees to improve confidence in assessing and managing older oncology patients.

Disclosure: None declared

Corresponding author: Cressida Lorimer

### **10. The role of dual modality imaging in the detection of residual nodal disease following radical chemoradiation for locally advanced head and neck cancer**

#### Vanita Gandhi^1^, Shelly English^2^, Anna Thompson^2^

##### ^1^*Royal Free NHS Trust*, ^2^*North Middlesex University Hospital*

**Background:** Adjuvant treatment following definitive chemoradiotherapy (CRT) for node-positive head and neck squamous cell carcinoma (HNSCC) has evolved from routine neck dissection (ND), towards risk-stratification with positron emission tomography with computed tomography (PET-CT). Patients with a complete response (CR) remain on clinical follow-up, and those with residual disease reported as an incomplete response (IR) have surgery. However, there remains no consensus for those with an equivocal response (ER).

The sensitivity of PET-CT is reported at 100% with specificity being only 84%. Studies have reported that combining diffusion-weighted magnetic resonance imaging (DW-MRI) with PET-CT improves specificity to 94%. Our centre uses both modalities at 3 months after completing CRT to assess treatment response. We retrospectively analysed the outcomes of these patients.

**Method:** Data was gathered from radiological reports and specialist multidisciplinary discussions for 43 patients diagnosed with advanced node positive HNSCC treated with definitive CRT at our centre from December 2015 to January 2018.

**Results:** CR, IR and ER were seen in 84% (n=36); 2% (n=1); and 14% (n=6) respectively on PET-CT. All DW-MRI scans reported those with residual lymphadenopathy as reactive or post-radiation change.

The patient with a radiological IR declined a ND and had a subsequent negative node biopsy. The node remains stable at one-year clinical follow-up. Of the 6 patients with ER: one underwent a ND and 0/39 nodes contained viable tumour cells. The remaining 5 patients are all in remission at a median follow up of 13.7 months.

**Conclusion:** These results demonstrate that combination imaging with PET-CT and DW-MRI may improve the evaluation of persistent lymphadenopathy following radical CRT. This may reduce the number of ‘equivocal’ responses identified and so avoid the need for salvage surgery, which is associated with additional morbidity and cost. We recommend performing a randomised control trial to examine this strategy further.

Disclosure: None declared

Corresponding author: Vanita Gandhi

### **11. Effects of a 12-week exercise and nutrition programme on cardiovascular fitness, body composition and psychological well-being in women at all stages of the breast cancer care continuum**

#### Joe Othen-Price^1^, Matthew Thompson^1^, Yvonne Rumble^1^, Sam Olden^1^, Ivor Cradock^1^

##### ^1^*Clinical Prevention and Rehabilitation, London, UK*

**Background:** It is well established that breast cancer treatment can have a deleterious effect on physical function, body composition and psychological well-being. It has been proposed that lifestyle factors, such as exercise and nutrition can have an important role in offsetting these negative changes, however, exercise programme guidelines remain generic and unvalidated. We propose that a 4 Pillars® framework provides an effective model for exercise and nutrition programmes for all breast cancer patients, from diagnosis through treatment and into survivorship.

**Method:** 24 females, aged 50 ± 10.6 years at various stages of the breast cancer care continuum completed a 12-week 1-2-1 programme at CP+R® based on 4 Pillars; twice-weekly resistance sessions, twice-weekly cardiovascular training within a prescribed heart rate zone, daily step-count monitoring and nutritional guidance. The programme was led by a clinical exercise specialist and overseen by a clinical nurse. All patients underwent cardiorespiratory fitness (VO_2_ peak) testing, body composition analysis and psychological profiling pre and post programme.

**Results:** There was a significant increase in VO_2_ peak, by 2.9 ± 4.1 ml/kg/min (p<0.05). Body fat percentage reduced by 1.8 ± 3.2 % (p<0.05) and dry lean mass increased by 0.3 ± 1.2 kg (p=0.25). Exercise and confidence score increased by 8.9 ± 16.2 % (p<0.05) and hospital anxiety and depression score reduced by 2.4 ± 6.1 points (p<0.05).

**Conclusion:** A 1-2-1 exercise and nutrition programme incorporating a 4 Pillar model significantly improved a range of physiological and psychological factors. The 4 Pillar model could provide a blueprint for exercise and nutrition programmes for breast cancer patients at all stages of the cancer care continuum.

Disclosure: None declared

Corresponding author: Joe Othen-Price

### **12. Headache suspicious of cancer - The Edinburgh early diagnosis pathway**

#### Karolis Zienius^1^, David Maxwell^2^, Lorna Porteous^2^, Janet Pooley^4^, David Summers^4^, Lesley McKinlay^4^, David Weller^1^, Helen Bulbeck^6^, Paul Brennan^6^, Robin Grant^7^

##### ^1^*University of Edinburgh*, ^2^*NHS Lothian, Edinburgh*, ^3^*Scottish Government*, ^4^*Western General Hospital, Edinburgh*, ^6^*Brainstrust*, ^7^*University Department of Neurosurgery, Edinburgh*

**Background:** Patients with “Headache Suspicious of Cancer” should be referred quickly for evaluation, especially if there is optic disc swelling or visual field loss. Previous work shows patients with tumour headaches often have asymptomatic cognitive findings. Based on this we developed an *Edinburgh Protocol Based Referral (PBR) Pathway*.

**Method:** The *Edinburgh PBR for “Headache suspicion of cancer"* incorporates an expedited GP open access CT (OACT) within 24-48 hours, if headache is associated with personality or cognitive change but in addition requires completion of a *semantic verbal fluency test* (SVFT) - a simple fast screening test of cognition (number of animals thought of in one minute). Also, if GP cannot see the discs or is uncertain about visual field defect, local optometry services will check the patient within 24-48 hours. The Edinburgh protocol is integrated into the electronic GP referral system *RefHelp* – with supporting information.

**Results:** In year 1, 20% (169/623) of all, and 74% (169/228) urgent, OACT scans came through the PBR pathway. Many forms were incompletely submitted. In forms completed correctly (including the SVFT) median time to scan was 2 days (1-7); Incomplete PBR was 9 days (3-13) and routine 13 days (9-16). 16.5% of all PBR requests were abnormal (3 had tumours: glioma 2, meningioma 1). Where SVFT was completed there was an association between abnormal CT scan and poorer SVFT (abnormal - median SVFT 13 (IQR 10-17); normal - median SVFT 19 (IQR 13-22).

The Optometry pathway is being promoted and is being considered for extension across Scotland in the new Scottish Cancer Referral Guideline (2018/19).

**Conclusion:** The Edinburgh PBR for “Headache suspicion of cancer" has been implemented. Expedited scans are being done. More work needs to be done around GP compliance with SVFT as this could be a valuable "risk factor" for secondary headache. Scottish Optometry support and promote the pathway.

Disclosure: Funded by The Brain Tumour Charity

Corresponding author: Robin Grant

### **13. Give us the tools... Ensuring consumer involvement adds value**

#### Richard Stephens^1^, Mat Baker^1^

##### ^*1*^*National Cancer Research Institute, UK*

**Background:** The cancer research community has long encouraged consumer involvement in the design, delivery and dissemination of research, including its oversight, regulation, funding and priority-setting. Recognition of the benefits that consumers bring to the research process has prompted the development of a number of toolkits that assist researchers in recruiting and using patients, carers and members of the public in cancer research. Less well developed are approaches to ensuring that consumer involvement is relevant and effective and that it adds the value that researchers, funders, patients and carers are seeking.

**Method:** The NCRI Consumer Forum provides consumer involvement services to the NCRI and to NCRI partners. It seeks to quality assure the contributions of its members and to ensure that these contributions are supported by training and development that enable members to operate to best effect. These collectively constitute an *expert practice model (EPM)* that carries a new recruit from selection and appointment to a Clinical Studies Group or other NCRI role, through induction training and developmental learning, supporting them to make a useful contribution in their individual role, and to add value to the wider contributions of the Consumer Forum as whole.

**Results:** Data from the Value and Impact Measures (VIM) indicate that this structured approach to the development of NCRI consumers’ expertise is enhancing the value and the positive impact of their contributions. Periodic reviews indicate that induction training and initial support, sponsored attendance at the NCRI Conference, tailored instruments to assist contributions, attendance at Forum meetings, involvement in Dragons Den events and the availability of the NCRI Consumer Toolkit are all regarded as helping to shape a coherent professional approach.

**Conclusion:** It is a goal of NCRI to improve the quality of research. Evidence is accumulating that the *EPM* is underpinning Consumer Involvement as a key enabler to deliver that goal.

Disclosure: Funded by National Cancer Research Institute, UK

Corresponding author: Richard Stephens

### **14. A quality toolkit for general practice: improving care after cancer treatment**

#### Paul Baughan^1^, Lorna Porteous^2^, Jean Sargeant^1^, Lorraine Sloan^1^, Andy Murphy^1^, Judith Mabelis^1^

##### ^*1*^*Macmillan Cancer Support*, ^*2*^*NHS Lothian*

**Background:** Primary Care has an important role in supporting people following a diagnosis of cancer. The ‘*Macmillan Cancer Care in Primary Care: a quality toolkit’* was developed to help GP practices and GP clusters improve care for people living with cancer.

**Method:** By using case studies, reflective practice, data collection and critical analysis, practices identified opportunities for improvements in current systems and processes as well as content of Cancer Care Reviews (CCR). Support and advice was offered from local Macmillan GPs and health board cancer leads.

Practices completed baseline and follow-up questionnaires, and a quantitative and qualitative evaluation was also undertaken.

**Results:** 251 Scottish GP practices (26% of total) from 12 health boards completed the toolkit. 190 practices (76%) completed both data collection questionnaires.

At baseline, 45% of practices had a robust system in place to routinely contact people after a diagnosis of cancer, increasing to 78% of practices following completion of the Toolkit (figure 1).

The range of topics discussed at a CCR increased following completion of the Toolkit (figure 2). More practices reported discussing the provision of cancer information (increased from 28% to 58%), the benefits of physical activity (increased from 25% to 46%), and the financial impact of a diagnosis (increased from 32% to 61%) at follow-up.

Following completion of the Toolkit, 142 GP practices (76% of total) reported that they felt better equipped to support people living with cancer by either a moderate or large extent (figure 3).

**Conclusion:** People with cancer often consult Primary Care professionals following their diagnosis. A significant proportion of Scottish GP Practices (26%) undertook the Macmillan Quality Toolkit. Following completion there is evidence of improved systems for contacting people following a diagnosis of cancer, a broader range of issues discussed at CCR, including further information regarding cancer, exercise, and the financial impact of diagnosis.

Disclosure: Funded by Macmillan Cancer Support

Corresponding author: Paul Baughan

### **15. A systematic review update on cost-effectiveness of colorectal cancer screening: identification of an optimal strategy in Europe**

#### Chih-Yuan Cheng^1^, Tao Ran^1^, Michael Schlander^1^

##### ^1^Division of Health Economics, German Cancer Research Center (DKFZ)

**Background:** Biennial guaiac-based faecal occult blood test (gFOBT), biennial faecal immunochemical test (FIT) and 10-yearly colonoscopy are the most commonly adopted colorectal cancer (CRC) screening strategies in Europe. Evidence regarding the effectiveness and cost-effectiveness of those strategies have been substantiated, yet there is no consensus on the most cost-effective modality. We aimed to identify a cost-effectively optimal CRC screening strategy among the three by conducting a systematic review.

**Method:** We searched PubMed, EMBASE, NHS EED, EconLit, and three other databases for European cost-effectiveness analyses evaluating any of the three strategies, published between January 2010 and December 2017. Two researchers independently conducted the review following the standard systematic review method. Net monetary benefit approach and incremental cost-effectiveness ratio (ICER) were used to assess the optimal strategy. Costs were converted to 2016 USD.

**Results:** We identified 17 European studies. Only two studies compared all three strategies– a French study measured the outcome with life-year gained (LYG) and a German study with quality-adjusted life-year (QALY). Both found biennial FIT cost-effectively optimal at and above the willingness-to-pay (WTP) threshold of $20,000 per LYG/QALY. If focusing on stool-based strategies, ten studies in total compared biennial FIT and gFOBT. Compared with gFOBT, FIT was cost-saving and more effective in two English and one German study, and had ICERs ranging from $2,406 to $11,310 per LYG or $478 to $2,591 per QALY in the remaining seven studies.

**Conclusion:** More evidence comparing all three strategies in Europe is needed, although two studies pointed at biennial FIT being cost-effectively optimal among the three at various WTP levels, as opposed to biennial gFOBT and 10-yearly colonoscopy. Between the two stool-based strategies, biennial FIT was shown to be highly cost-effective (at $50,000 WTP threshold), or even cost-saving. Our findings support the trend in Europe shifting from gFOBT to FIT in CRC screening programmes.

Disclosure: None declared

Corresponding author: Chih-Yuan Cheng

### **16. The development of an exercise focused self-management app (iExhale) for lung cancer survivors to improve their symptom control**

#### Catherine Henshall^1^

##### ^1^*Oxford Brookes University, UK*

**Background:** Lung cancer affects over 33,000 people per year in the UK and many of these have significant smoking related co-morbidities, which can negatively impact on their activities of daily living and subsequent quality of life. Common symptoms experienced include breathlessness, fatigue and depression. Exercise practices have been shown to improve symptom control in lung cancer patients and exercise based self-management mobile applications have the potential to provide tailor made, individualised care to lung cancer survivors and decrease pressure on NHS services.

The aim of this study was to design and develop an exercise-focused self-management mobile application for lung cancer survivors to improve their symptoms of breathlessness, fatigue and depression.

**Method:** The study design consisted of three stages. Stage 1 was a systematic review to examine the impact of exercise interventions in improving breathlessness, fatigue and depression in lung cancer survivors. Stage 2 consisted of qualitative focus groups with lung cancer patients, carers and health professionals to explore their views on the usefulness of exercise in improving symptom control. Findings from Stages 1 and 2 informed Stage 3 which consisted of mobile application development and a usability study.

**Results:** Stages 1 and 2 found evidence that exercise does improve symptom control for lung cancer survivors and that exercises need to be carefully tailored to meet the preferences and needs of individual lung cancer survivors. These findings were used to develop the content and format of the mobile application, which was further modified following a usability study with lung cancer survivors.

**Conclusion:** A working, theoretically underpinned, patient centred exercise mobile application (iExhale) has now been developed to help improve the symptom control of lung cancer survivors. Plans are to test the feasibility and acceptability of the application in the community setting, before conducting a full scale randomised controlled trial.

Disclosure: Funded by Oxford Brookes University

Corresponding author: Catherine Henshall

### **17. Living with and beyond cancer with comorbid illness: a qualitative systematic review**

#### Debbie Cavers^1^, Liset Habets^2^, Sarah Cunningham-Burley^1^, Eila Watson^3^, Elspeth Banks^4^, Christine Campbell^1^

##### ^*1*^*University of Edinburgh*, ^*2*^*University of Leiden*, ^*3*^*Oxford Brookes University*, ^4^*Independant*

**Background:** There is a need to explore the experience of the growing number of people living with and beyond cancer with additional long term chronic conditions, with implications for cancer survivorship management and support. This review aims to identify the qualitative evidence on the experience of cancer and comorbid illness from the perspective of patients, carers and health care professionals to identify psycho-social support needs, experience of health care, and to highlight areas where more research is needed.

**Method:** PRISMA guidance was used to review the evidence. Relevant research databases were searched using an exhaustive list of search terms. Two reviewers independently screened titles and abstracts and discussed variations. Included articles were subject to quality appraisal before data extraction of article characteristics and findings. Thematic synthesis of extracted findings was undertaken following Thomas and Harden’s prescribed method.

**Results:** 30 articles were included in the review covering a range of cancer types and comorbid conditions; with varying time since cancer diagnosis and apparent severity of disease for both cancer and other conditions. Studies are set in developed countries and include the views of patients and professionals but not carers. Few studies focused exclusively on the experience of living with comorbid conditions alongside cancer. Key themes identified included: the interaction between cancer and comorbid conditions; the added symptom burden; illness identities and ageing; self-management; prioritising conditions, and treatment decision-making.

**Conclusion:** In addition to a better understanding of the complex experience of such illness to illuminate developing models of patient-centred care, the review will combine with patient engagement work to inform an interview study with the defined patient group.

Acknowledgement: A version of this abstract has been published previously, see https://onlinelibrary.wiley.com/doi/10.1002/pon.4639 for original and CC-BY license.

Disclosure: Funded by CSO

Corresponding author: Debbie Cavers

### **18. Changes in physical activity and quality of life in men with prostate cancer participating in a physical activity behaviour change pathway**

#### Louis Fox^1^, Nicola Peat^2^, Isla Veal^2^, Mieke Van Hemelrijck^1^

##### ^*1*^*Translational Oncology and Urology Research, King's College London*, ^*2*^*Guy's and St Thomas' NHS Foundation Trust*

**Background:** Exercise has been demonstrated to be beneficial to men with prostate cancer (PCa). However, maintaining these exercise behaviours remains a challenge. This study examined a 12-month person-centred physical activity behaviour change pathway, delivered through a London-based NHS cancer centre, and determined changes in self-reported physical activity (PA) and self-reported quality of life (QoL) over the course of 12 months.

**Method:** 221 men diagnosed with PCa were given the option of exercising independently/in the community; or undergoing 8-12 weeks of supervised exercise, followed by independent/community exercise. All men were given an initial 1-hour consultation, subsequently followed up via telephone at 4, 7, and 12 months. All men were offered motivational interviewing, aimed at encouraging exercise behaviour, at all time points.

**Results:** 68% (n=150) of men remained engaged with the pathway at 12 months. This retention rate was similar across treatment groups. Overall, there were increases in self-reported PA compared with baseline, which were significant at 4 and 7, but not 12 months. The trend for increases in PA amongst men on curative treatment showed significance at 4 months compared with baseline, but not at 7 or 12 months. Men undergoing systemic treatment reported increases in PA compared with baseline which were significant at all follow up time points. There was a trend toward increases in reported QoL over time, with significant increases at 4 and 12 months compared with baseline. Men undergoing curative treatment showed the most pronounced changes in QoL.

**Conclusion:** Men with PCa who receive a 1-hour PA behaviour change consultation, followed by optional supervised exercise classes for 8-12 weeks, and behaviour change follow-up telephone consults tend to report increases in PA levels and QoL over 12 months, compared with baseline. Improvements in PA and QoL were most apparent for men on systemic and curative treatment, respectively.

Disclosure: Funded by Guy's and St Thomas' NHS Foundation Trust; Mr Christopher Cottrell (independent donor)

Corresponding author: Louis Fox

### **19. Impact of psychological counselling service at Penny Brohn UK**

#### Jo Durrant, Helen French, Marian Naidoo, Sarah Churchward, Michelle Griffiths, Helen Seers

##### *Penny Brohn UK*

**Background:** For people with cancer, research has shown counselling may be beneficial for wellbeing, anxiety, depression and helping with the sexual side-effects of cancer. Penny Brohn UK (PBUK) is a cancer charity that supports people to live well with cancer. It offers face-to-face or telephone counselling for anyone aged over 18 with a cancer diagnosis and their close supporters.

**Method:** PBUK’s counselling service was evaluated using the validated person-centred outcome measure MYCaW (Measure Yourself Concerns and Wellbeing), measuring the impact of cancer support services on the severity of people’s cancer related concerns and wellbeing. Concerns were rated at the start of counselling and re-rated at the end of the last session. Qualitative data was captured on the MYCaW tool regarding other things going on in people’s life affecting health and what was important about the service they received. Data was analysed using the accompanying qualitative coding framework.

Data was analysed for all clients who had attended counselling appointments in January- December 2017 and provided full pre- post-counselling MYCaW data.

**Results:** 40 clients provided pre-post MYCaW data and attended an average of 6 sessions (range 1 to 18). Psychological and emotional concerns were top rated for concern 1 and 2 (81% and 78% respectively). Prior to counselling, concerns were rated fairly severely (5.1/6 for concern 1 and 2; 6 being the worst). After counselling concerns showed a statistically significant improvement (2.2/6 and 2/6 for concern 1 and 2 respectively, p<.000 for both). 90% of clients showed a clinically significant improvement in their concerns. Qualitative data indicated that counselling at PBUK gave clients the opportunity to talk, provided time for themselves and made them feel supported and understood.

**Conclusion:** Counselling at PBUK led to a statistically significant improvement in cancer-related concerns and wellbeing. A larger sample is needed to explore this further.

Disclosure: Funded by Penny Brohn UK

Corresponding author: Helen Seers

### **20. An exploratory study on the use of game-based learning using Microsoft Kinect to teach oncology phase I clinical trial designs**

#### Alan Bilsland^1^, Caroline Kelly^2^, Jennifer Roccisana^3^, James Paul^2^, Rob Jones^2^, Joanne Edwards^3^, Antonia Roseweir^4^, Torsten Stein^3^, Katherine West^5^

##### ^*1*^*Institute of Cancer Science, Wolfson Wohl Cancer Research Centre, University of Glasgow*, ^*2*^*Cancer Research UK Clinical Trials Unit, Institute of Cancer Science, University of Glasgow*, ^*3*^*Institute of Cancer Science, University of Glasgow*, ^*4*^*School of Medicine, Dentistry and Nursing, University of Glasgow*, ^*5*^*School of Life Sciences, University of Glasgow*

**Background:** Phase I trials are the first stage in drug development. Major objectives include determining toxicities of new agents and maximum tolerated dose (MTD) for later clinical stages. The 3+3 design is most common, although support is growing for newer “model-based” designs. One criticism of these is their complexity. To investigate gamification in teaching these designs to MSc/BMedSci Cancer Science students, a computer game using Microsoft Kinect motion sensing was used to supplement existing lectures.

**Method:** The game objective is to find MTD of “drug X”. Groups of 3-5 receive a draft trial protocol, pre-clinical toxicology report, and instructions for allometric scaling to human dose. Players first decide dose ranges to investigate. Using the game, patient cohorts are recruited, administered drug X, and toxicities checked. Players decide if dose limiting toxicity (DLT) is observed and whether to dose-escalate/de-escalate, expand the cohort, or stop. Each group plays a different trial design. Feedback was taken using a questionnaire investigating technology interaction, group working, learning outcomes and engagement.

**Results:** The game was tested with 3 student cohorts using different control methods (student-led, tutor-led, or mixed). 7/10, 7/8, and 12/21 students consented to analysis of their responses. Across cohorts, learner engagement items scored particularly highly. Nearly all also reported improved understanding of the concepts of DLT and MTD. Many also reported improved understanding of different phase I designs. Mann-Whitney analysis of responses under the different control conditions revealed few significant differences. Our favoured approach is mixed-mode control.

**Conclusion:** In the clinical trials education literature, studies of active learning approaches appear to be sparse. We have not yet formally evaluated student-reported learning improvements in a test/re-test setting. However, positive feedback across items of our questionnaire suggests that students value the activity. We envisage potential future extensions to the game addressing aspects of phase I design beyond recruitment and dosing.

Reference: Microsoft Kinect, https://marketplace.xbox.com/en-GB/Product/Kinect-for-Xbox-360/66acd000-77fe-1000-9115-d8025858084b

Disclosure: Funded by Glasgow Experimental Cancer Medicine Centre (funded by Cancer Research UK and the Chief Scientist Office, Scotland); University of Glasgow

Corresponding author: Alan Bilsland

### **21. Successes and challenges in the implementation of the HOPE self-management programme for people living with and beyond cancer**

#### Gemma Pearce^1^, Sally Pezaro^1^, Joanne Parsons^1^, Andy Turner^1^, Paramjit Gill^2^, Tracey Norris^3^

##### ^*1*^*Coventry University*, ^*2*^*University of warwick*, ^*3*^*Associate Learning and development Manager and regional lead for H.O.P.E Macmillan Cancer support*

**Background:** The HOPE Programme© is a self-management course developed with people living with and beyond cancer and licensed by Macmillan Cancer Support to be delivered across the UK. Despite this, delivery is inconsistent across regions. The aim of this research was to examine barriers and successes of implementation to improve future delivery.

**Method:** 10 focus groups and 27 interviews were completed across 6 UK regions using an Interpretive Description approach. Participants (n=73) were recruited by Macmillan gatekeepers for being involved in the promotion and delivery of the HOPE Programme (Macmillan staff, commissioners, healthcare professionals, attendees and facilitators). Analysis was completed using Framework Analysis.

**Results:** Barriers to implementation included getting the course up-and-running regularly, incorporating the programme into the care pathway, buy in from professionals, time, money, venue, supporting people returning to work, recruiting men, younger people and BME groups.

Successes of implementing the course occurred when Macmillan staff, healthcare professionals, volunteers and management were involved in the plans from the beginning, actively referring and encouraging course retention. Cancer Information Centres were useful hubs to recruit onto the course. Patients liked the friendly neutral space and professionals found it useful to signpost patients. The most successful time to refer patients to the course was after being discharged from treatment when patients often felt isolated like “falling off of a cliff”. Professionals liked having the HOPE Programme to refer patients to meet their unmet psychosocial needs.

**Conclusion:** These findings help understand barriers further and provide examples of how some sites have successfully overcome them. Some felt that more of a nationwide recruitment strategy and advertisement scheme would be beneficial, encouraging regions to incorporate the programme into the patient’s care pathway. Sharing these lessons of implementation will hopefully improve implementation consistency across the nation, increase the impact of the course and inform the implementation science field.

Disclosure: Funded by Coventry University

Corresponding author: Gemma Pearce

### **22. Introduction of treatment summaries and holistic needs assessments for teenagers and young adults after cancer treatment in Scotland: a national feasibility study**

#### Angela Edgar^1^, Nicola Davison^1^, Simita Kumar^1^, Bernadine Wilkie^1^, Jeff White^2^

##### ^*1*^*NHS Lothian*, ^*2*^*NHS Greater Glasgow and Clyde*

**Background:** The Scottish Government recommends that all teenagers and young adults (TYA) treated for cancer, receive a Treatment Summary (TS) and Holistic Needs Assessment (HNA) to aid communication, education, self-management and identify areas of concern. The objectives of this study were: i) To determine the feasibility of implementing TS and HNA for Scottish TYA after cancer treatment; and ii) to share the TS with health professionals to improve communication and engagement.

**Method:** Scottish TYA (16-24 years) completing cancer treatment, between April 2016 -April 2017, were identified at the weekly National TYA Multidisciplinary Team (TYA MDT) meeting. Treatment Summaries and HNA were completed and distributed to patients and health professionals.

**Results:** Of 83 eligible patients, 51 were recruited (response rate 61%); 26 (51%) males, median (range) age 22 (16 – 25) years. Among non-recruits: 2 relocated, 1 relapsed, lead consultant deemed the process ‘not relevant’ (n=6), or did not respond (n=23). Cancer diagnoses: germ cell tumours (39%), lymphomas (27%) and bone tumours (14%) over represented; carcinoma (8%), melanoma (2%) and CNS tumours (2%) under-represented.

**Conclusion:** Treatment Summaries and Holistic Needs Assessments were completed for almost two thirds of TYA identified after cancer treatment. Recruitment failure was due largely to consultant disengagement. Based on incidence data, current pathways do not allow identification of almost half of patients at the end of treatment. Completion of TS/HNA is labour intense and adequate time and training must be provided. By sharing Treatment Summaries, we have improved communication and engagement with Primary Care and provided clear pathways for referral of patients to hospital when problems arise.

Disclosure: Funded by Macmillan Cancer Care

Corresponding author: Angela Edgar

### **23. Holistic needs assessments for teenagers and young adults after cancer treatment in Scotland identified a significant burden of unmet needs**

#### Angela Edgar^1^, Nicola Davison^1^, Simita Kumar^1^, Bernadine Wilkie^1^, Jeff White^2^

##### ^*1*^*NHS Lothian*, ^*2*^*NHS Greater Glasgow and Clyde*

**Background:** Psychosocial issues are common amongst Teenagers and young adults (TYA) after cancer treatment and TYA report feeling unsupported. The objective of this study was to determine the burden of needs amongst TYA after cancer treatment and evaluate whether these needs are being met.

**Method:** Scottish TYA (16-24 years) completing cancer treatment, between April 2016-April 2017, were identified. Treatment Summaries (TS) and Holistic Needs Assessment (HNA) were completed and distributed (TS only) to patients and health professionals. Qualitative analysis identified concerns.

**Results:** Fifty-one of 83 eligible TYA were recruited (response rate 61%); 26 (51%) males, median (range) age 22 (16 – 25) years with over-representation of germ cell tumours (39%), lymphomas (27%), bone tumours (14%). TS data was available for 51 (100%) and raw HNA data for 26 patients (50%). 45 TYA (88%) reported concerns: 35 (69%) reported 1-5 concerns; 2 (4%) reported 6-10 concerns; 5 (10%) reporting 11-15 concerns; 1 (2%) reported 1 concern and 2 (4%) reported >20 concerns: general appearance, physical fitness and emotional issues, were the most numerous. One third of TYA were referred for psychological support; two-thirds benefitted from third sector support programmes.

**Conclusion:** Almost 90% of TYA reported at least one concern after cancer treatment, with more than two-thirds of patients reporting five to 10 concerns, and 10% reporting up to 15 concerns. While support services, largely provided by third sector, are in place for many patients, further evaluation of the Health Needs Assessments, exploring relationships to diagnoses, and identification of gaps in services, are required to inform future developments.

Disclosure: Funded by Macmillan Cancer Care

Corresponding author: Angela Edgar

### **24. Identifying and counting people living with treatable but not curable cancer in the England cancer registry**

#### Rachel White^1^, Joanna Pethick^2^, Archie Macnair^3^, Gregory Fallica^1^, Jennifer Than^4^, Jane Maher^1^

##### ^*1*^*Macmillan Cancer Support*, ^*2*^*National Cancer Registration and Analysis Service, Public Health England*, ^*3*^*Macmillan Cancer Support & The Royal College of Radiologists*, ^*4*^*Macmillan Cancer Support & National Cancer Registration and Analysis Service, Public Health England*

**Background:** There is a growing cohort of people who, although they cannot be cured of their cancer, are on treatments that can reduce cancer burden, alleviate symptoms and prolong life. This is a heterogeneous group with different prognoses and treatments, which could be described as living with treatable but not curable cancer (TNCC).

This project aims to build a search criterion to identify the TNCC population using the England cancer registry.

**Method:** A set of possible search criteria were developed for evaluation by 20 oncologists, haematologists and specialist nurses. Through their expertise and analysis, we will refine this into a single, comprehensive search criterion.

**Results:** Our first criterion was based on distant metastatic cancer at diagnosis. There were 76,000 people in England in 2015 diagnosed either at stage IV cancer, with a secondary malignancy or with certain haematological cancers. Our next criterion focused on metastatic disease developed post-diagnosis. It included 135,000 people identified in inpatient HES and 10,000 from the Cancer Waiting Times dataset.

Our next set of criteria focused on treatment. This selected 46,000 people who, in 2015, received one of 236 chemotherapy regimens thought to target TNCC; or had a second chemotherapy treatment over a year from their first treatment. It found 7,000 people who had a second round of radiotherapy after a six-month gap. Another criterion pinpointed 99,000 people who received palliative chemotherapy or radiotherapy.

In the final searches we identified 57,000 people diagnosed with an intermediate survival cancer and 78,000 with a shorter-term survival cancer. McConnell et al. (2017) hypothesised that intermediate survival cancers are often long-term conditions. New treatments will make the shorter-term survival group an important part of TNCC.

**Conclusion:** The criteria will not cover all circumstances but, ultimately, this project will allow better recognition of people with TNCC and help appropriate services to be provided for them.

Disclosure: Funded by Macmillan Cancer Care

Corresponding author: Rachel White

### **25. A randomised controlled trial to assess the impact of regular early specialist symptom control treatment on quality of life in malignant mesothelioma – ‘RESPECT-Meso’**

#### Fraser Brims^1^, Samal Gunatilake^2^, Iain Lawrie^3^, Laura Marshall^2^, Carole Fogg^4^, Cathy Qi^5^, Nick Maskell^6^, Karen Forbes^6^, Najib Rahman^5^, Steve Morris^7^, Stephen Gerry^5^, Anoop on behalf of the RESPECT-Meso investigators Chauhan^2^

##### ^*1*^*Sir Charles Gairdner Hospital*, ^*2*^*Portsmouth Hospitals NHS Trust*, ^*3*^*North Manchester General Hospital*, ^*4*^*University of Portsmouth*, ^*5*^*University of Oxford*, ^*6*^*University of Bristol*, ^*7*^*University College London*

**Background:** Malignant pleural mesothelioma (MPM) is a cancer with a high symptom burden and a median survival of less than one year. Evidence from other cancer types suggests there may be some benefit in health related quality of life (HRQoL) with the integration of early specialist palliative care (SPC) with existing oncological services, but the level of certainty of evidence is low.

**Method:** Participants with newly diagnosed MPM were randomised to early SPC integrated with standard care, or standard care alone, in a 1:1 ratio. Main carers were recruited additionally. Quality of life (QoL) and mood were assessed at baseline and every 4 weeks for up to 24 weeks with the EORTC QLQ–C30 questionnaire for QoL and General Health Questionnaire (GHQ-12) for anxiety/depression. The primary outcome was the change in EORTC C30 Global Health Status (GHS) QoL 12 weeks after randomisation.

**Results:** Between April 2014 and October 2016 we randomised 174 participants. The two groups were well matched after randomisation. There was no significant between group difference in QoL score at 12 weeks (mean difference adjusted for baseline between groups 1.8 (95% CI: -4.9 to 8.5; p = 0.59)). QoL did not differ at 24 weeks (mean difference adjusted for baseline -2.0 (95% CI: -8.6 to 4.6; p = 0.54)). There was no difference in depression/anxiety scores at 12 or 24 weeks. In carers there was no between group difference quality of life and mood at 12 or 24 weeks, although there was a consistent preference for care, favouring the intervention arm.

**Conclusion:** There is no role for routine referral to SPC soon after diagnosis of MPM for patients who are cared for in specialist centres with good access to SPC when required.

References: QLQ-C30 - Aaronson NK, Ahmedzai S, Bergman B, Bullinger M, Cull A, Duez NJ, et al. The European-Organization-For-Research-And-Treatment-Of-Cancer QLQ-C30 – A quality-of-life instrument for use in international clinical trials in oncology. J Natl Cancer Inst 1993;85:365–76. https://www.ncbi.nlm.nih.gov/pubmed/8433390

GHQ-12 - Goldberg, D., & Williams, P. (1988). A user’s guide to the General Health Questionnaire. Windsor, UK: NFER-Nelson https://books.google.co.uk/books/about/A_User_s_Guide_to_the_General_Health_Que.html?id=LpSuGQAACAAJ&redir_esc=y

Clinical Trial Registry: ISRCTN18955704; http://www.isrctn.com

Disclosure: Funded by British Lung Foundation and Australian Communities Grant

Corresponding author: Cathy Qi

### **26. Prevalence, characteristics and outcomes of bone metastases in patients with neuroendocrine neoplasms (NENs)**

#### Hussain Raja^1^, Kok Haw Jonathan Lim^2^, Paolo D'Arienzo^2^, Jorge Barrisuso^3^, Mairéad McNamara^3^, Richard Hubner^2^, Was Mansoor^2^, Juan Valle^3^, Angela Lamarca^2^

##### ^1^The Christie NHS Foundation Trust, ^2^Department of Medical Oncology, The Christie NHS Foundation Trust, Manchester, United Kingdom, ^3^Department of Medical Oncology, The Christie NHS Foundation Trust, Manchester, United Kingdom. Division of Cancer Sciences, University of Manchester, Manchester, United Kingdom

**Background:** Bone metastases (BMs) from NENs are rare. Updated guidelines for adequate management of BMs in NENs are lacking, with current practice based on recommendations for other malignancies.

**Method:** This is a retrospective study of patients with NENs (any grade); patients with grade 3 NENs were eligible if non-lung primary. BMs were classified as symptomatic in the presence of pain, hypercalcaemia and/or skeletal-related events (SRE; pathological fracture or metastatic spinal cord compression). Median survival was estimated using Kaplan-Meier analysis (SPSS V.23.0, https://www.ibm.com/analytics/spss-statistics-software).

**Results:** Of 1459 patients screened, 85 (7.0%) were identified to have NEN-related BMs. Median age was 58 years (IQR 47.5 – 67.5). Most patients were male (56.5%), and had non-functional (43.5%), grade 1/2 (81.2%), gastro-entero-pancreatic (49.4%) NENs. Only 28.2% of patients presented with BMs at first diagnosis of metastatic disease. The median time from diagnosis of metastatic disease to identification of BMs was 4 months (95%CI 0.0 – 8.2). The most frequent pattern of BMs was widespread (61.2%) (oligometastatic 32.9%, unknown 5.9%). Most patients were asymptomatic (58.8%) at initial diagnosis of BMs, although a majority (77.6%) later develop BM-related symptoms (pain/hypercalcaemia 63.5%, SRE 20.0%). In this cohort, BMs were managed mainly with analgesia (43.5%). Radiotherapy and bisphosphonates were used in 34.1% (36.5% when analysis limited to patients with BM-related pain) and 21.2% (26.9% when analysis limited to patients with widespread BMs), respectively. Surgery was rarely performed (2.4%). Median overall survival from identification of BMs was 31.0 months (95% CI 19.6 – 24), and from the time of development of BM-related symptoms was 18.9 months (95%CI 8.7 – 29.1).

**Conclusion:** BMs, albeit rare in NENs, frequently become symptomatic. Radiotherapy and bisphosphonates are used less frequently in NENs. Increased awareness and earlier interventions may improve patients’ quality of life following diagnosis/symptoms of BMs.

Disclosure: Funded by The Christie NHS Foundation Trust

Corresponding author: Hussain Raja

### **27. Patient-reported outcomes in men with advanced and localised disease: Results from the UK-wide Life After Prostate Cancer Diagnosis study**

#### Amy Downing^1^, Penny Wright^1^, Sarah Wilding^1^, David Donnelly^2^, Luke Hounsome^3^, Eila Watson^4^, Richard Wagland^5^, Hugh Butcher^1^, Paul Kind^1^, Peter Selby^1^, James Catto^6^, William Cross^7^, Dyfed Huws^8^, David Brewster^9^, Anna Gavin^2^, Adam Glaser^1^

##### ^*1*^*University of Leeds*, ^*2*^*Queens University Belfast*, ^*3*^*Public Health England*, ^*4*^*Oxford Brookes University*, ^*5*^*University of Southampton*, ^*6*^*University of Sheffield*, ^*7*^*Leeds Teaching Hospitals NHS Trust*, ^*8*^*Public Health Wales*, ^*9*^*University of Edinburgh*

**Background:** Prostate cancer (PCa) outcome studies frequently focus on localised cancer and little is known regarding advanced disease. The Life After Prostate Cancer Diagnosis (LAPCD) study is a large-scale population-wide evaluation of patient-reported outcomes in men with PCa, including all stages of disease and all treatments. Here we report the functional outcomes and health-related quality of life (HRQL) of men with advanced and localised disease.

**Method:** Men diagnosed 18–42 months previously were identified through cancer registration data in each United Kingdom (UK) nation. Postal surveys were used to collect data on functional outcomes (EPIC-26 plus interventions for sexual dysfunction) and generic HRQL (EQ-5D-5L and self-assessed health [SAH; rated 0-100]), alongside sociodemographic and treatment information.

**Results:** 35,823 (60.8%) men responded; median age 71 years. Stage at diagnosis was known for 85.8% of respondents; 63.8% stage I/II, 23.5% stage III, 12.8% stage IV. Overall HRQL reports were good. Poor sexual function was common (78.9%), regardless of disease stage and few men received help for this (medication: 41.4%; devices: 22.6%; specialist services: 14.8%; 56.5% offered no intervention). Androgen deprivation therapy (ADT) use was associated with poorer HRQL, hot flushes and lack of energy. A quarter of men with stage IV disease reported no problems on any EQ-5D dimension (compared to 42.1% stage I/II, 36.4% stage III). SAH was 6 points lower in men with stage IV disease (71.6) compared to men with localised cancer (77.8) but this difference was greater in younger men.

**Conclusion:** 18-42 months after diagnosis of PCa, a high proportion of men report sexual dysfunction and less than half were offered intervention. Loss of HRQL through ADT is common and is more pronounced in younger men. The good overall HRQL allows clinicians to present positive goals for quality of survival after PCa, including for many diagnosed with advanced disease.

Disclosure: Funded by the Movember Foundation, in partnership with Prostate Cancer UK (grant number BO26/MO)

Corresponding author: Adam Glaser

### **28. Home renal monitoring for cancer patients: technical assessment and patient acceptability**

#### Leanne Ogden, Donal Landers, Jenny Royle, Laura Stephenson, Laura Hutchinson

##### *Digital Experimental Cancer Medicine Team*

**Background:** Recruitment to cancer clinical trials is challenging and usually limited to patients with preserved kidney function. Restrictions that allow those with an eGFR >50ml/min is arbitrary and not a risk-based approach driven by current clinical science. Due to increased survival rates for both conditions, there is now a significant population who have both cancer and chronic kidney disease. The aim of this research was to assess whether new technological advances in point of care (POC) creatinine meters and digital science could be used to give these people access to oncology clinical trials through personalised risk-based monitoring. We created an approach that explored the potential and acceptability of using a POC device, data capture via a smartphone, and risk-categorisation through an Acute Kidney Injury (AKI) algorithm, to enable decision-making and the first step in addressing this unmet clinical need.

**Method:** Three POC devices were evaluated for usability, size and complexity. A smart phone app was developed, which captures device data and sends securely to a Cloud environment. Creatinine testing, calibration and patient acceptability in the hospital was conducted over a 2 week period with 17 interactions (patient/carer/nurse) and including two patient focus groups.

**Results:** The Nova Biomedical Creatinine StatSensor® was the preferred device chosen to enable home creatinine readings with good user feedback and stable performance characteristics. The smartphone app user interface design was acceptable with patients based on patient acceptability testing- producing near-real time creatinine readings which could be reviewed by the medical team.

**Conclusion:** This proof of concept demonstrated that creatinine can be measured by a POC device, the data captured by an app and reported in near-real time. Further work is now being conducted to develop a clinical rules engine based on the NHS/NICE published algorithm for AKI; and apply this in clinical trials to alert investigators to impending AKI.

Disclosure: Funded by AstraZeneca

Corresponding author: Leanne Ogden

### **29. Patient reported experiences (PREs) from the PERSEPHONE early breast cancer (EBC) trial**

#### Janet Dunn^1^, Maggie Wilcox^2^, Claire Balmer^1^, Sophie Gasson^1^, Louise Hiller^1^, Anne-Laure Vallier^3^, Claire Hulme^4^, Kerry Raynes^1^, Donna Howe^1^, Helen Higgins^1^, David Miles^5^, Andrew Wardley^6^, David Cameron^7^, Helena Earl^3^

##### ^*1*^*University of Warwick*, ^*2*^*Independant Cancer Patients’ Voice (ICPV), London, UK*, ^*3*^*Cambridge University Hospitals*, ^*4*^*University of Leeds*, ^*5*^*Mount Vernon Cancer Centre*, ^*6*^*The Christie Clinical Research Facility*, ^*7*^*University of Edinburgh*

**Background:** PERSEPHONE is a Phase 3 randomised non-inferiority trial comparing 6 months of trastuzumab to the standard 12 months in patients with HER2 positive EBC. An important component of the trial was collecting information about patient experiences. Collecting ‘quasi-qualitative’ data via open-ended questions is becoming increasingly common and championed as a way to add depth to assessments and complement quantitative survey data.

**Method:** Alongside the toxicities reported on the trial case report forms (CRF) and patient booklets being collected, including quality of life (QoL) and Health Care Resource Usage, patients were invited to record comments about the study and their treatment. Experiences were recorded prior to commencement of trastuzumab, then 3-monthly for a year, then every 6 months up to year 2. Within a mixed methods framework, both the trial researcher and patient representatives explored the information collected using a thematic content analysis.

**Results:** Between Oct’07 and Jul’15, 4088 patients were randomised. In total, 5542 experiences were recorded from 2456 patients across the 6 time-points. Patients offered information on all aspects of the study, including their views on the treatment, their care, the QoL questionnaire and the research itself. Most often mentioned was the impact the treatment had on participants personally - physically, psychologically or socially. Most frequently cited were aches and pains and fatigue; for many, these did appear to be particularly distressing and intractable. In parallel, the CRFs reported 20% of patients reporting a grade 3/4 toxicity during treatment (23% 12 month, 18% 6 month, p=0.004), with significantly higher rates of cough, pain, fatigue, chills and palpitations reported by 12 month patients (p<0.05).

**Conclusion:** Patients’ experiences during and beyond trastuzumab highlighted the long-term cumulative effects of their treatment and confirm that patients do suffer from burdensome toxicity with dramatic impact on their QoL.

Disclosure: Funded by NIHR Health Technology Assessment (HTA)

Corresponding author: Janet Dunn

### **30. Evaluation of Penny Brohn UK’s national programme of ‘Living Well With and Beyond Cancer’ services**

#### Helen Seers^1^, Michelle Griffiths^1^, Sarah Churchward^1^, Rebecca Samuel^2^, Marian Naidoo^1^, Helen French^1^, Rachel Jolliffe^1^, Jo Durrant^1^

##### ^*1*^*Penny Brohn UK*, ^*2*^*University of Bath*

**Background:** Penny Brohn UK (PBUK) is a leading UK charity specialising in helping people live well with the impact of cancer. National Lottery’s Big Lottery funding between 2015-18 has supported PBUK Living Well with and Beyond Cancer (LWWBC) services in five areas of the UK. Services include Living Well courses (LWC), Follow-Up (FU) support.

**Method:** Pre and 6-weeks post-LWWBC questionnaires used were: Patient Activation Measure (PAM) for health self-management, Measure Yourself Concerns and Wellbeing (MYCaW) for concerns and wellbeing perception, and Patient Reported Experience Measure for personal reflection on support gained.

**Results:** 145 courses (123 LWC and 22 FU), located in North England (8), South Coast (32), Midlands (29), South East (43) and South West (33) are running over 3 years. 901 people with cancer enrolled in the first 2 years with between 12-23% (111-206) evaluation postal response rate for pre-post paired data. Year 3 is still underway. Years 1 and 2 data as follows: 43% (48/111) had a meaningful improvement in their post Patient Activation Measure scores indicating they were more likely to go on to self-manage their health. 87% (163/188) of people stated they had been better able to self-manage their own health after LWWBC services.70% (144/206) had a clinically significant improvement in their MYCaW main cancer related concerns, and 43% (89/205) had a clinically significant improvement in MYCaW wellbeing.There were reported improvements in: diet (87% 174/199), exercise (74% 143/193) relationship issues (63% 120/189) and use of stress management techniques (90% 179/199). Of the 199 respondents, 65% reported subsequently attending a meditation or group and 60% joined an exercise class after the LWWBC. Patterns of improvement in MYCaW cancer-related concerns and wellbeing were reported across all regions, demonstrating a successful expansion of PBUK support services across England.

**Conclusion:** PBUK’s LWWBC evaluation responses indicate a positive and potentially beneficial experience.

Disclosure: Funded by Penny Brohn UK

Corresponding author: Helen Seers

### **31. Evaluation of a Chemotherapy Closer to Home model for breast cancer patients**

#### May Teoh^1^, Nicola Daws Twilley^2^, Emma Bond^3^, Judith Dua^2^, Regina Santos^4^, Sara Wills Percy^1^, Victoria Mumford^1^, Susan Dargan^2^

##### ^*1*^*Royal Surrey County Hospital NHS Foundation Trust*, ^*2*^*Ashford and St Peter's Hospital NHS Foundation Trust*, ^*3*^*Ashford and St Peter'sHospital NHS Foundation Trust*, ^*4*^*Ashord and St Peter's Hospital NHS Foundation Trust*

**Background:** The Chemotherapy Closer to Home model is aimed at improving patient experience by facilitating more convenient access to treatment for patients closer to their homes. A joint initiative by Ashford and St Peter’s Hospital (ASPH) and Royal Surrey County Hospital (RSCH) successfully launched a chemotherapy outreach service at Ashford Hospital so patients could receive treatment locally. The service at Infusion Suite Ashford (ISA) commenced with breast cancer patients and an evaluation of the service was conducted.

**Method:** Patients who attended ISA for breast cancer treatment in 2016 were included. Data on patient demographics, treatment details and time-distance savings were analysed. A patient feedback survey was conducted to evaluate patient satisfaction and experience.

**Results:** 108 breast cancer patients received treatment at ISA in 2016 (total 883 attendances). Median age was 60 years (range 26 – 89 years). Median number of attendances per patient was 7 (range 1-20). 53.7% had stage IV disease and were receiving palliative treatment. 61% of attendances were for systemic anti-cancer therapy, 29.1% for supportive treatment (e.g. Zoledronic acid) and 9.3% for both. Average round-trip distance for patients treated at ISA was 9.8 miles per attendance, compared to 40.8 miles if treated at RSCH. Average round-trip time for patients treated at ISA was 27.4 minutes, compared to 56.7 minutes if treated at RSCH. Of survey respondents (n = 17), 100% rated their overall care as excellent or very good. Satisfaction scores were high for support and information given during chemotherapy (e.g. advice on managing side effects).

**Conclusion:** The chemotherapy outreach service at ASPH has successfully provided a high quality, convenient option for patients to receive treatment closer to home. This is especially valuable as a significant proportion of patients have advanced disease and often require multiple attendances for palliative treatment. The service is now expanding to include other tumour sites.

Disclosure: None declared

Corresponding author: May Teoh

### **32. Future of clinical trials after Brexit**

#### Zoe Martin^1^, Nerea Cuadra^2^, Peter Glenday^2^, Guy Yeomans^2^

##### ^*1*^*Cancer Research UK*, ^*2*^*School of International Futures*

**Background:** Brexit has introduced uncertainty over the future of clinical trials and the UK's ability to collaborate with the EU. There is a need for a more developed evidence base on how to ensure a successful future for clinical trials. Cancer Research UK (CRUK) commissions policy research to inform our thinking and to develop a wider evidence base for positive change.

**Method:** The School of International Futures are delivering the study using a futures approach based on horizon scanning, trend analysis and scenario planning to explore the future of clinical trials. The project had 4 phases: (1) Identification of what is shaping the future of clinical trials; Rapid evidence assessment of literature; 23 interviews with researchers, patients, clinicians, industry, government and regulators in the UK, EU and internationally. (2) Online survey assessment to gather sector views on the most important and uncertain drivers of change and to gather additional perspectives. (3) Scenario development – five potential scenarios were developed for the future of clinical trials. (4) A participative workshop with the 38 participants explored the scenarios to identify implications and develop recommendations.

**Results:** The study identifies implications of the different potential Brexit scenarios on the future of clinical trials, grouped into five themes. For each theme, there are recommendations aimed to ensure a successful future of clinical trials and a positive outcome for patients. Results will be finalised by July 2018.

**Conclusion:** Findings and recommendations will be used to inform policy makers during negotiations of the UK’s exit from the EU and to ensure the best outcome in any Brexit scenario. There is interest in the study from policy makers who were informed and involved during the study, including attending our workshop.

Disclosure: Funded by Cancer Research UK

Corresponding author: Zoe Martin

### **33. Cisplatin-induced kidney injury is transient and associated with short-term elevation of urine interleukin-18 in patients with testicular cancer**

#### Alan Cameron^1^, Kelly McMahon^2^, Paul Welsh^1^, Ashita Waterston^3^, Michael Zappitelli^4^, Jeff White^3^, Patrick Mark^1^, Rhian Touyz^1^, Ninian Lang^1^

##### ^*1*^*University of Glasgow*, ^*2*^*McGill University*, ^*3*^*Beatson West of Scotland Cancer Centre*, ^*4*^*University of Toronto*

**Background:** Cisplatin causes acute kidney injury (AKI) but changes in urinary AKI biomarkers are not well defined clinically. We investigated short- and intermediate-term AKI using novel biomarkers over 9 months in patients with testicular cancer treated with cisplatin.

**Method:** Prospective observational study of men with testicular cancer in 3 groups following orchiectomy: 1) surveillance; 2) adjuvant cisplatin-based chemotherapy (1-2 cycles); 3) metastatic cisplatin-based chemotherapy (3-4 cycles). Blood and urine was collected at 6 visits: baseline, 24h, 6 weeks, 3, 6 and 9 months for renal injury markers.

**Results:** 27 men (median age 34y [IQR 31-40y]) were recruited: surveillance (N = 10); adjuvant cisplatin (N = 7); metastatic cisplatin (N = 10). Urinary renal injury biomarkers (interleukin-18 [IL-18], neutrophil gelatinase-associated lipocalin [NGAL], vascular endothelial growth factor [VEGF], kidney injury molecule-1 [KIM-1], albumin/creatinine ratio [ACR]) and serum cystatin C (CysC) were elevated at 24h and 6 weeks (KIM-1) post-cisplatin in adjuvant and metastatic groups (all: P<0.05 vs. baseline). These normalized by 6 weeks (IL-18; NGAL; VEGF; ACR; CysC) and 3 months (KIM-1). eGFR was within normal range throughout.

**Conclusion:** Novel biomarkers indicate cisplatin nephrotoxicity is reversible over 3 months. Elevation of IL-18 supports a major inflammatory renal insult. This provides evidence to support investigation of anti-inflammatory drugs for cisplatin nephrotoxicity prevention.

Disclosure: Funded by British Heart Foundation

Corresponding author: Alan Cameron

### **34. Information giving in breast cancer: A service review of the Mid-Yorks NHS Trust**

#### Georgina Appleyard^1^, Hannah Jones^1^, Jay Naik^2^

##### ^*1*^*University of Leeds*, ^*2*^*Mid Yorks NHS*

**Background:** This study aimed to understand patient information needs and explore information giving in the Mid-Yorks Breast Cancer Service.

**Method:** A service evaluation was conducted via a questionnaire distributed to patients at outpatient-clinics across Mid-Yorks Hospitals and was completed by 53 patients.

**Results:** Patient experience was largely positive; 91% of participants reported fully understanding the explanation of their breast cancer diagnosis and 89% felt involved in treatment decisions. The majority of patients (62%) preferred verbal information, closely followed by written information (26%), email (4%) and video (2%). Verbal and written information combined was viewed as most effective, providing greatest patient satisfaction and opportunities to clarify with questions, aiding later recall. Yet, the older participants preferred written information above all else. The majority of patients (83%) wanted to be told as much as possible about their cancer; however, some only wished to know important details about their diagnosis. Patients valued their doctor and nurses equally as information-givers, due to their respective skillsets. Communication style was important, with patients requiring sympathetic, straightforward and honest healthcare professionals who are able to provide care tailored to the individual patient. Clinical Nurse Specialists were found to be vital for high patient satisfaction and a care plan useful in individualising care.

**Conclusion:** The results highlight the need for clear and honest information provision by healthcare professionals that is focused to suit the needs and preferences of the patient. Personalised care plans for each patient, along with provision of contact details for a Clinical Nurse Specialist, can help facilitate this. Future research could involve implementing an information checklist as part of breast cancer patient care to ensure all patients’ information needs are provided for. In summary, all healthcare professionals must ensure that effective tailored communication is at the heart of the care they provide to improve patient satisfaction.

Disclosure: Funded by University of Leeds

Corresponding author: Hannah Jones

### **35. “…you can feel..a little isolated if you haven’t got the contact”; qualitative analysis of an innovative service to support prostate cancer survivors with radiotherapy late effects (the EAGLE study)**

#### Stephanie Sivell^1^, Elin Baddeley^1^, John Staffurth^2^, Sophia Taylor^3^, Annmarie Nelson^1^

##### ^*1*^*Marie Curie Palliative Care Research Centre, Division of Population Medicine, Cardiff University*, ^*2*^*Division of Cancer and Genetics, School of Medicine, Cardiff University; Velindre Cancer Centre*, ^*3*^*University of Southamptom*

**Background:** Prostate cancer survivors may experience bowel gastrointestinal side effects of radical radiotherapy for prostate cancer. These symptoms can lead to severe difficulties including limiting work, travel or socialising. To improve the care for patients with bowel problems, the EAGLE study implemented an innovative gastroenterological service in three NHS centres (two led by a specialist nurse). The purpose of the qualitative analysis was to assess the acceptability of the new service and monitor the experiences of patients and professionals.

**Method:** Semi-structured interviews were held with healthcare professionals and patients at baseline, 6 months and 12 months. Interviews were audio-recorded and transcribed verbatim; data were analysed using framework analysis.

**Results:** Thirty-five healthcare professionals (Baseline n = 17; 6 months n = 13; 12 months n = 2) and 16 patients (including their companions: Baseline n=9; 6 months n = 5; 12 months n = 2) were interviewed. Four key themes emerged from the qualitative analyses: *1) Making a difference:* patients reported the information they were given to manage their symptoms to be useful and effective, making a positive difference and providing support they did not have previously; *2) capacity:* both patients and professionals were supportive of the service, hoping that this service could continue after the research study; *3) Expertise:* patients and professionals were supportive of the nurse-led model - the professionals found this freed-up consultation time; *4) Barriers and facilitators:* patients were happy to both join the service and the research study to evaluate the service, although some patients did not differentiate a difference between the two.

**Conclusion:** The new service was well received by both patients and professionals. Patients felt the new service has provided them with support, helping them to relieve and improve long-standing side effects of bowel radiotherapy. We will triangulate additional health economics and statistical data to determine the overall value of the service and explore its expansion beyond research.

Disclosure: Funded by Prostate Cancer UK

Corresponding author: Stephanie Sivell

### **36. Trends in mortality from malignant melanoma: an observational study of the World Health Organisation mortality database from 1985 to 2015**

#### Dorothy Yang^1^, Justin Salciccioli^2^, Dominic Marshall^3^, Joseph Shalhoub^4^

##### ^*1*^*Royal Free London NHS Foundation Trust*, ^*2*^*Department of Medicine, Mount Auburn Hospital*, ^*3*^*Oxford University Clinical Academic Graduate School, John Radcliffe Hospital*, ^*4*^*Academic Section of Vascular Surgery, Department of Surgery and Cancer, Imperial College London*

**Background:** Malignant melanoma (MM) has the highest mortality among skin cancers. MM incidence is reported to have increased over the past decades, particularly in regions with predominantly fair-skinned populations. We report 30-year global MM mortality trends using the World Health Organisation (WHO) mortality database.

**Method:** An observational analysis of the WHO mortality database between 1985 and 2015 was performed. ICD-9 and 10 codes for MM were used to extract age-standardised death rates (ASDRs) for all countries classified as having high usability death registration data by the WHO. Trends were described using Joinpoint regression.

**Results:** 33 countries were included in this analysis. For both sexes, 3-year average ASDRs for 2013-2015 were highest in Australia and Slovenia and lowest in Japan: rates of death were 5.72/100 000, 3.86/100 000, and 0.24/100 000 respectively in males, and 2.53/100 000, 2.58/100 000, and 0.18/100 000 respectively in females. In all countries, MM mortality remained greater in males than females across the observation period. All countries demonstrated increased mortality rates in males over the observation period except Czech Republic, which demonstrated a single decreasing trend in mortality on Joinpoint analysis (estimated annual percentage change -0.7%). More countries exhibited decreasing or stable MM mortality in females, with Israel and Czech Republic demonstrating the greatest percentage decreases in mortality rates over the observation period (−23.4% and −15.5% respectively).

**Conclusion:** There is a persisting global sex disparity in MM mortality over the past 30 years. In some regions, this is due either to greater increases in mortality rates in males compared to females, or to decreasing or stabilising mortality trends in females not paralleled in males. Future work will explore potential explanatory factors for the observed trends and sex disparity in MM mortality.

Disclosure: None declared

Corresponding author: Dorothy Yang

### **37. Phase II randomised control trial of a nutrition and physical activity intervention after radical prostatectomy for prostate cancer**

#### Lucy Hackshaw-McGeagh^1^, Chris Penfold^1^, Ellie Shingler^2^, Athene Lane^2^, Richard Martin^1^

##### ^*1*^*University of Bristol*, ^*2*^*University of bristol*

**Background:** Dietary factors and physical activity may alter prostate cancer progression. We explored the feasibility of lifestyle interventions following radical prostatectomy for localised prostate cancer.

**Method:** We recruited patients into a pre-surgical observational cohort; following their radical prostatectomy, we offered the men randomisation into a 2x3 factorial randomised controlled trial (RCT). This involved randomisation into both a modified nutrition group (either increased vegetable and fruit and reduced dairy milk; or lycopene supplementation; or control) and a physical activity group (brisk walking or control). Outcomes were collected at trial baseline, three and six months, with daily adherence reported throughout. Primary outcomes were measures of feasibility: randomisation rates and intervention adherence at six months follow-up.

**Results:** 108 men entered the pre-surgical cohort and 81 the post-surgical RCT (randomisation rate: 93.1%). Of 25 men in the nutrition intervention, 10 (40.0%; 95% CI: 23.4%-59.3%) adhered to the fruit and vegetable recommendations and 18 (72.0% 95% CI 52.4%-85.7%) to reduced dairy intake. Adherence to lycopene (n = 28), was 78.6% (95% CI: 60.5%-89.8%), whilst 21/39 adhered to the walking intervention (53.8%; 95% CI: 38.6%-68.4%). Most men were followed up at six months (75/81; 92.6%).

**Conclusion:** Interventions were deemed feasible, with high randomisation rates and good adherence.

Clinical Trial Registry: ISRCTN:99048944; http://www.isrctn.com

Disclosure: Funded by NIHR

Corresponding author: Athene Lane

### **38. Living with and beyond cancer: Self-managing chemotherapy side-effects and eating behaviours**

#### Gianina-Ioana Postavaru^1^, Hilary McDermott^2^, Fehmidah Munir^2^, Tanweer Ahmed^3^

##### ^*1*^*Bishop Grosseteste University*, ^*2*^*Loughborough University*, ^*3*^*United Lincolnshire Hospitals NHS Trust*

**Background:** With improved cancer survival, more people are experiencing short- and long-term chemotherapy side-effects, such as reduced taste and smell. These effects may be temporary or permanent and are associated with altered quality of life due to reduced food enjoyment, weight loss, changed patterns of food intake and social activities linked to eating and drinking, emotional distress and interference with daily life. Taste and smell problems are difficult to diagnose and treat in clinical oncology settings, often because of a lack of routine assessment practices. The aim of this study was to gain insight from people living with and beyond cancer about their experiences of chemotherapy side-effects and strategies used to manage their eating behaviours and diet.

**Method:** Data was collected through in-depth face to face semi-structured interviews with people living with and beyond cancer in the East Midlands of England. Data were analysed using interpretative phenomenological analysis (IPA).

**Results:** Data analysis led to a narrative organized in two parts. The first part (retrospective understanding of chemotherapy side-effects experiences) reports on the experience of temporary and permanent reduced taste and smell, and their impact on eating behaviours and quality of life, as well as feelings of desolation contrasted to exceptional received support. The second part explains strategies used by participants to manage their health, barriers to and facilitators of access to information and professional advice.

**Conclusion:** This retrospective investigation of survivors’ experienced chemotherapy side-effects gave access to aspects of their experience that often remain undiscussed with healthcare professionals. Further research is needed to develop a taxonomy of taste and smell alterations and food hedonics, which may give clinicians better diagnostic clues to the precise nature of these challenges and inform the design of interventions to ameliorate specific treatment-related side-effects.

Disclosure: Funded by Bishop Grosseteste University

Corresponding author: Gianina-Ioana Postavaru

### **39. Relapse rate and relapse patterns in patients undergoing curative resection for Pancreatic Ductal Adenocarcinoma (PDAC): Identifying high risk patients**

#### Akul Purohit^1^, Abdullah Malik^2^, Rahul Deshpande^2^, Mairead McNamara^3^, Thomas Satyadas^2^, Melissa Frizziero^4^, Saurabh Jamdar^2^, Rille Pihlak^5^, Aali Sheen^2^, Ajith Siriwardena^2^, Richard Hubner^4^, Derek O'Reilly^2^, Juan W Valle^3^, Nicola De Ligouri Carino^2^, Angela Lamarca^4^

##### ^*1*^*The Christie NHS Foundation Trust*, ^*2*^*Hepatobiliary & Pancreatic Surgical Team, Department of Surgery, Manchester Royal Infirmary, Manchester, UK*, ^*3*^*Medical Oncology Department, The Christie NHS Foundation Trust, Manchester, UK & Division of Cancer Sciences, University of Manchester, Manchester, UK*, ^*4*^*Medical Oncology Department, The Christie NHS Foundation Trust, Manchester, UK*, ^*5*^*Medical Oncology Department, The Christie NHS Foundation Trust, Manchester, UK & Division of Cancer Sciences, University of Manchester, Manchester, UK*

**Background:** PDAC has a 5-year relapse rate >80% following curative resection. Identifying patients at higher risk of relapse and factors related with specific patterns of disease spread may inform selection of candidates for closer follow-up.

**Method:** The outcome of patients with PDAC (Jan’05-Sep’17) who underwent curative resection, and with available follow-up and relapse status data, was analysed retrospectively. The aim was to identify factors associated with higher relapse risk and specific relapse patterns. Kaplan Meier, Log-rank, Chi squared and T-tests were used for analysis.

**Results:** Of 228 patients, 182 were eligible. Patient characteristics: 55.5% male, median age 65.7 years (95%CI 64.2-67.2), T3 (81.9%), lymph node (N)1 (71.4%), involved resection margin (R)1 (61.5%). Adjuvant chemotherapy was given to 113 patients (62.1%) [5-fluorouracil (5-FU)(38.9%), Gemcitabine (G) (31.9%), G+5-FU (29.2%)-based]. Median overall survival (OS) was 21.6 months (95%CI 17.9–28.9). After a median follow-up of 17.9 months, 143 patients relapsed [relapse rate 78.6%, median time-to-relapse 8.9 months (95%CI 7.7–10.1)]. Median OS was shorter (log-rank p-value <0.001) in the “relapse” group (17.5 months (95%CI 15.9-20.9) vs. “non-relapse” (not reached).

Patients with “relapse” had higher rate of N1 disease (76.2% vs 53.8%; p-value 0.0161). No other factors related with increased relapse rate were identified. Local relapse (59.4%): resection bed (54.6%) +/- regional N (16.8%). Distant relapse (69.9%): median number of organs affected was 1; liver 41.3%, lung 20.9%, peritoneum 11.9%, distant N 4.9%, bone 2.8%, others 2.1%. Three relapse patterns were identified: regional only (30.1%), distant only (40.6%) and combined (regional + distant) (29.3%). Adjuvant chemotherapy reduced the risk of combined relapse (p = 0.04).

**Conclusion:** Relapse rate following curative resection for PDAC remains high, with predominance for distant recurrence. Patients with N1 disease have the highest risk of relapse and may benefit from closer follow-up.

Disclosure: None declared

Corresponding author: Akul Purohit

### **40. Living with and beyond colorectal cancer: ColoREctal Well-being (CREW) study at five years**

#### Claire Foster^1^, Sally Wheelwright^1^, Lynn Calman^1^, Samantha Sodergren^1^, Joanne Haviland^2^, Amanda Cummings^1^, Jane Winter^4^, Amy Din^1^, Deborah Fenlon^4^, Janis Baird^1^, Jessica Corner^5^, Alison Richardson^3^, Peter W Smith^1^, Study Advisory Committee Members^1^

##### ^*1*^*University of Southampton*, ^*2*^*Institute of Cancer Research*, ^*3*^*University of Southampton, University Hospital Southampton NHS Foundation Trust*, ^*4*^*Swansea University*, ^*5*^*University of Nottingham*

**Background:** The ColoREctal Well-being (CREW) study is the first study to prospectively recruit a representative sample of colorectal cancer (CRC) patients, carry out the first assessment pre-treatment and then follow up longitudinally over five years in order to explore the impact of treatment on health and wellbeing.

**Method:** CRC patients from UK cancer centres received questionnaires at baseline (pre-surgery), 3, 9, 15, 24, 36, 48 and 60 months. Quality of life (QOL), self-efficacy (confidence to manage illness-related problems), mental health, social support, affect, socio-demographics, clinical and treatment characteristics were assessed. Data were analysed using multivariate statistical techniques.

**Results:** A representative cohort of 872 non metastatic CRC patients were recruited from 29 UK cancer centres. Most participants recovered well after curative treatment but around 30% had poor psychosocial outcomes and this persisted up to the 5 year follow-up^1^. Baseline psychosocial factors (particularly self-efficacy and depression) were more important than disease stage and location of tumour in determining who is most likely to have problems over the first 5 years^1^. Self-efficacy remained stable over time whereas social support declined for a significant number of participants. Both low self-efficacy and perceived decline in social support were associated with poorer outcomes ^1-3^. Comorbidities that limit an individual’s typical daily activities were associated with poorer health and wellbeing outcomes.

**Conclusion:** This unique study provides robust evidence that psychosocial factors, such as self-efficacy, are important predictors for the longer term outcomes of CRC patients. We call for early assessment and intervention, including assessment of depression and confidence to manage illness related problems and limiting co-morbidities, from diagnosis onwards. Early assessment would identify those most likely to need support in their recovery. Early intervention has the potential to reduce need and improve outcomes throughout treatment and beyond.

Disclosure: Funded by Macmillan

Corresponding author: Lynn Calma

### **41. CTCAE-based comprehensive toxicity assessment following radical radiotherapy for head & neck squamous cell carcinoma (HNSCC)**

#### David Noble^1^, Amy Bates^2^, Jessica Scaife^3^, Jo Gemmill^2^, Richard Benson^2^, Gill Barnett^2^, Sarah Jefferies^2^, Neil Burnet^4^, Raj Jena^1^

##### ^*1*^*Cambridge University, Department of Oncology*, ^*2*^*Cambridge University NHS Foundation Trust*, ^*3*^*Gloucestershire Hospitals NHS Foundation Trust*, ^*4*^*University of Manchester, Division of Cancer Sciences*

**Background:** Radiotherapy is an effective but toxic treatment for head and neck squamous cell carcinoma (HNSCC). Many published studies report data on a narrow range of side effects and time-points, and a more comprehensive understanding of post-radiotherapy sequelae is required.

**Method:** All HNSCC patients recruited to the consolidation cohort of the CRUK-VoxTox study were included. Patients were treated with IG-IMRT on TomoTherapy, and standard radical protocols. Clinician-assessed toxicity was prospectively recorded at baseline, months 3, 6, 12, then annually to year 5, using CTCAEv4.03.

**Results:** 188 patients were included with median follow-up 20 months. Primary sites: oropharynx 122, larynx 18, oral cavity 16, maxilla 9, CUP 7, nasopharynx 6, hypopharynx 5, skin 5. 39 underwent neck dissection before definitive radiotherapy, 149 did not. Concomitant SACT: 106 received cisplatin, 15 cetuximab, 67 none. Rates of Grade 2+ toxicity are reported in the following table.

**Conclusion:** The range of toxicities and time-points presented demonstrate that most peak 3 months after radiotherapy, and gradually improve thereafter. Dysphagia and salivary gland toxicity are commonest. Dry mouth appears to improve with time, whilst dysphagia is more persistent. Further research, treatment development, and clinical trials are all necessary to reduce the incidence of these toxicities, and improve the quality of life of patients living beyond cancer.

Disclosure: Funded by Cancer Research UK

Corresponding author: David Noble

Table 1 [Abstract 41].

**Table:** 

Gr2+ Toxicity (%)	Baseline	3m	6m	12m	24m	36m	48m
Dry mouth	2.7	78.8	64.7	58.5	45	34.6	31.6
Salivary duct Inflammation	3.1	58	48.7	36.7	28.5	25	26.3
Dysphagia	5.9	36.8	26.9	17	18.7	19.2	21.1
Nausea	0.5	2.3	2.5	0	0	0	0
Anorexia	6.4	25.3	17	12.3	9.9	5.8	5.3
Trismus	3.8	14.9	9.2	6.8	5.5	9.6	0
Hoarseness	7.5	37.9	25.6	23.8	20.9	13.4	10.5
Hearing Impaired	12.2	17.2	14	13.6	15.4	19.2	15.8
Tinnitus	1.6	4.6	3.7	4.8	4.4	3.8	0
Cataract	2.1	0	1.2	0.7	2.2	1.9	5.3

### **42. Process mining to explore variation in chemotherapy pathways for breast cancer patients**

#### Angelina Kurniati^1^, Geoff Hall^2^, David Hogg^3^, Owen Johnson^3^

##### ^*1*^*University of Leeds; Telkom University*, ^*2*^*University of Leeds; Leeds Teaching Hospital*, ^*3*^*University of Leeds*

**Background:** There is concern that standard chemotherapy pathways of six cycles scheduled every two or four weeks reflect administrative and operational needs rather than patients’ personal and biological needs. Process mining of routine data can help identify and explore common pathway variants.

**Method:** We extracted anonymised records from routine data at Leeds Cancer Centre for breast cancer patients with a first diagnosis between 2004 and 2013 with an adjuvant chemotherapy pathway (n = 738). This produced an event log data file (containing events, dates and times) which was analysed using the ProM and DISCO process mining tools. We used the Inductive Miner plug-in and constructed statistical and visual models of the clinical pathways. The data covered a ten-year period and we created multiple splits of the event log to examined statistically significant variations over time.

**Results:** Most patients varied from the expected pathway (712 variants for 738 patients). We produced a pathway model which included these variants and checked conformance. Overall fitness of data to model was high (97.1%) but we noted significant changes to the fit in 2006 (a 5.1% change) and 2011 (8.9% change) which require further investigation. In total 51% (n = 376) of patients did complete all six cycles, less than half (21% of total, n = 158) completed the cycles without an adverse event while many (30%, n = 218) experienced at least one adverse event including missed appointments, neutropenic sepsis and emergency admissions. Of the 49% (n=362) who did not complete six cycles, 28% (n=207) experienced adverse events with the remainder (21%, n=155) not completing for other reasons.

**Conclusion:** Process mining of routine data showed extensive variation from standard chemotherapy pathways including incomplete treatment and adverse events. Future work is needed to explore potential causal links and understand changes in the pathway over time.

Ethics number: IRAS206843, https://www.hra.nhs.uk/planning-and-improving-research/application-summaries/research-summaries/ .

Disclosure: Funded by ClearPath Connected Health Cities Project, the Indonesia Endowment Program for Education (LPDP)

Corresponding author: Angelina Kurniati

### **43. The Northern Oncology Trainees Collaborative for Healthcare Research (NOTCH): A new trainee-led collaborative initiative for the evaluation of non-surgical cancer outcomes**

#### Christopher Jones, Caroline Dobeson, Matthew Howell, Anna Olsson-Brown, Eileen Parkes, Abdulazeez Salawu

##### *Northern Oncology Trainees Collaborative for Healthcare Research*

**Background:** Insufficient time, competing priorities and the limitations of drawing inferences from underpowered, single-centre populations are commonly cited barriers to the effective study of real-world cancer outcomes. Doctors not in a dedicated academic post may also lack sufficient training to undertake research, despite recognition from medical bodies of the importance of research education. In a number of surgical disciplines, trainee-led research collaboratives spanning multiple institutions are now well established as a means to meeting these challenges. To our knowledge, there have been no previous attempts at establishing similar approaches in non-surgical oncology (NSO). The Northern Oncology Trainees Collaborative for Healthcare Research (NOTCH) has recently been established to promote trainee-led NSO outcomes research. We provide here an overview of this group’s aims, scope and early activities.

**Method:** Two clinical and two medical oncology trainees were invited to lead the initiative from each of six large cancer centres within the north of England and Northern Ireland. Trainees from each centre are supported by a wider advisory group consisting of senior clinicians and academics. A call for project proposals was launched between March-May 2018, with proposals submitted by trainees in partnership with senior clinicians.

**Results:** Twelve proposals were received, ten of which focussed on a specific malignancy and two of which focussed on outcomes across a number of cancers. All but one of the proposals involved retrospective data analysis. Proposed outcomes included analysing compliance with existing guidelines, the development of prognostic indices and analyses of toxicity, recurrence and survival patterns. One or more of the proposals will be selected to be undertaken by trainees from across the six centres.

**Conclusion:** NOTCH provides a novel trainee-led approach for collaborative multi-centre real-world NSO outcomes research for which there is evidence of engagement from trainees and senior clinicians alike.

Disclosure: None declared

Corresponding author: Christopher Jone

### **44. Cancer and pregnancy: A national and tertiary unit experience**

#### Arti Kara^1^, Angela Yulia^2^, Pete Wallroth^3^, Jacque Gerrard^3^, David Williams^4^

##### ^*1*^*University College London Hospitals NHS Foundation Trust & UCL*, ^*2*^*University College London Hospitals NHS Foundation Trust*, ^*3*^*Mummy’s Star*, ^*4*^*University College London Hospitals NHS Foundation Trust and UCL*

**Background:** Cancer during pregnancy affects approximately 1:1000 pregnancies, but is becoming more common. More women are becoming pregnant having survived childhood cancer. The lack of UK data on cancer and pregnancy outcomes has meant management is often idiosyncratic and not evidence-based. We aim to establish a UK Registry for pregnant women affected by cancer to improve cancer and pregnancy outcomes for affected women and children.

**Method:** We report data collected by ‘Mummy’s Star’, a UK charity dedicated to the support of women and their families affected by cancer during pregnancy and up to one-year post-partum. We also collected data on pregnancies affected by cancer at University College London Hospital (UCLH).

**Results:** Mummy’s Star reported a total 419 women of whom 257 (61%) were diagnosed with cancer during pregnancy and 162 (39%) were diagnosed postnatally. Breast cancer was the commonest malignancy affecting 180 (43%) women. Thirty two (8%) women had died and approximately one third of women had metastatic cancer.

In a preliminary data collection at UCLH we identified 30 women; 23 were cancer survivors and 7 were diagnosed with cancer during pregnancy. The majority, 17 (57%) were aged between 32 and 40 years old. Hodgkin lymphoma was the commonest malignancy in 7 (23%) patients. Chemotherapy was delayed until childbirth for 3 (10%) patients. Three (10%) patients suffered from anxiety and depression during pregnancy. All women were alive 42 days post-delivery.

**Conclusion:** Cancer during pregnancy is a heterogeneous and relatively rare condition. No single centre can gain enough experience to provide evidence-based care for optimal pregnancy outcome. A national Registry in association with Mummy’s Star will build on an established data-set to accelerate our understanding of best practise for these vulnerable women and their families.

Acknowledgement: A version of this abstract has been published previously, see https://obgyn.onlinelibrary.wiley.com/doi/abs/10.1111/1471-0528.15191 for original and CC-BY license.

Disclosure: Funded by Mummy's Star which is self funded by charitable funds

Corresponding author: Arti Kara

### **45. Updated polygenic risk score in breast cancer patients is not associated with increased radiotherapy toxicity**

#### Gillian C Barnett^1^, Leila Dorling^2^, Laura Fachal^2^, Morag Brothwell^1^, Charlotte E Coles^1^, John R Yarnold^3^, Neil G Burnet^4^, Paul DP Pharoah^2^, Catharine ML West^4^, Alison M Dunning^2^

##### ^*1*^*Cambridge University Hospitals NHS Foundation Trust*, ^*2*^*Centre for Cancer Genetic Epidemiology, University of Cambridge*, ^*3*^*Institute of Cancer Research & Royal Marsden NHS Foundation Trust*, ^*4*^*University of Manchester*

**Background:** It has been hypothesized that increased genetic predisposition to breast cancer may increase risk of toxicity following breast irradiation. Polygenic risks scores (PRS) can be generated from genotypes at breast cancer susceptibility loci by summing risk-allele dosages. A polygenic risk score including 90 genotypes previously showed no association with increased risk of early or late radiotherapy toxicity at 5 years. Recent developments in the Breast Cancer Association Consortium (BCAC) have increased the number of confirmed breast cancer risk variants to a set of 352 at all the risk loci identified to date.

**Method:** Common variant genotypes were determined in 1134 breast cancer patients from the RAPPER (Radiogenomics: Assessment of Polymorphisms for Predicting the Effects of Radiotherapy) study using the Illumina CytoSNP12 genome-wide array. A further 15,582,449 genotypes were imputed using the 1000 Genomes Project reference panel. Univariable and multivariable regression analyses were performed to assess association between both polygenic risk score and the 352 individual variants with radiotherapy toxicity at 2 years including telangiectasia, breast oedema, photographic shrinkage, induration, pigmentation, breast pain, breast sensitivity and overall toxicity.

**Results:** After correction for multiple testing, no association was found between polygenic risk score and development of late radiotherapy toxicity. On multivariable analysis of individual variants, rs138944387 was significantly associated with breast pain (beta = 1.12; 95% CI 0.62-1.61; p = 1.09 x 10^−5^) and rs17513613 was associated with risk of breast oedema (beta=-0.21; 95% CI -0.31 to -0.12; p = 2.01 x 10^−5^).

**Conclusion:** Cancer patients with a high polygenic predisposition to breast cancer calculated using an updated polygenic risk score do not have increased risk of radiotherapy toxicity up to two years following radiotherapy. The association between 2 individual variants and late toxicity require validation in independent cohorts.

Disclosure: Cancer Research UK

Corresponding author: Gillian C Barnett

### **46. Feasibility of a community pharmacy lifestyle intervention to increase physical activity and improve cardiovascular health of men after prostate cancer treatment**

#### Sara Faithfull^1^, Agnieszka Lemanska^1^, Karen Poole^1^, Bruce Griffin^1^, Ralph Manders^1^, John Marshall^2^, John Saxton^3^

##### ^*1*^*University of Surrey*, ^*2*^*Prostate Cancer UK*, ^*3*^*Northumbria University*

**Background:** Evidence that physical activity improves prostate cancer treatment outcomes is now recognised. This research explores the feasibility and acceptability of a community pharmacy lifestyle intervention for improving physical activity and health related outcomes. Pharmacy-led health assessment provide opportunities for lifestyle prescription in cancer survivors, we used a computerised algorithm to support personalisation for physical activity and diet. This was repeated at three months and supported by two phone calls and instructional materials in nine community pharmacies.

**Method:** Particpants included men with non-metastatic prostate cancer, completed cancer treatment and at least one of the three risk factors: underweight, overweight or obese; active androgen deprivation therapy; hypertension. Physical and functional fitness were measured with Siconolfi step test and physical activity was assessed objectively using accelerometry. Upper body strength (grip strength), lower body strength (sit-to-stand test) and cardiovascular health (QRISK2 algorithm including weight, body mass index, blood pressure and cholesterol) were also assessed. Feasibility and acceptability were evaluated.

**Results:** 403 men were eligible, 172 (43%) responded and 116 (29%) were subsequently recruited. We report 15% attrition as 99 (85%) men completed the pharmacy intervention. The intervention was feasible and acceptable for men. At three months compared to baseline scores, men’s moderate to vigorous physical activity (MVPA) increased by an average of 34 minutes (95% CI: 6 to 62, P = 0.018). The level of MVPA was not sustained at six months (average increase was 14 minutes, 95% CI: -27 to 54, P = 0.509). QRISK2 score was reduced at 3 months (P = 0.001).

**Conclusion:** This study demonstrates that a community pharmacy-led intervention is feasible and acceptable to non-metastatic prostate cancer patients. The increase in physical activity and improved health, support the development of this delivery approach after cancer treatment. An adequately powered randomised controlled trial is needed to assess the relative cost-efficacy of this intervention.

Disclosure: Funded by Movember Foundation and Prostate Cancer UK

Corresponding author: Sara Faithfull

### **47. Ethnicity and the tumour characteristics of breast cancer in a large nationally representative sample of women in England**

#### Toral Gathani^1^, Isobel Barnes^1^, John Broggio^2^, Gillian Reeves^1^

##### ^*1*^*University of Oxford*, ^*2*^*Public Health England*

**Background:** Some studies have suggested that ethnic minority women have more aggressive breast cancer compared to White women. However, the evidence is limited and inconsistent, and has generally not accounted for sociodemographic differences. Complete data on tumour characteristics by ethnicity are available from Public Health England for over 68000 breast cancers registered between 2006 and 2013 and are reported here.

**Method:** The data analysed includes patient characteristics (age, deprivation and ethnicity) and tumour characteristics (size, grade, nodal status and receptor profile). Each tumour characteristic was dichotomised and treated as the outcome variable in a logistic regression model yielding odds ratio (OR) for each characteristic by ethnicity adjusted for age, region, deprivation, and all other tumour characteristics.

**Results:** There were 66,192 breast cancers in White women, 1233 in South Asian women and 641 in Black women. The mean age at diagnosis was five years younger in South Asians and Blacks compared to Whites (55 versus 60 years). In unadjusted analyses, both South Asian and Black women were more likely than White women to have higher risks of more biologically aggressive tumour factors including higher grade, larger size, ER negativity and node positive tumours. However, after adjustment for age in particular, and other factors, these differences between the ethnic groups were reduced substantially. For example, compared to White women, the unadjusted and adjusted OR for grade 3 tumours was 1.49 (95%CI 1.33-1.67) and 1.20 (95%CI 1.05-1.37) for South Asian women, and 1.94 (95%CI 1.66-2.27) and 1.44 (95%CI 1.20-1.72) for Black women. Similarly, compared to White women, the unadjusted and adjusted OR for node positive cancer was 1.32 (95%CI 1.18-1.48) and 1.16 (95%CI 1.03-1.31) for South Asian women and 1.60 (95%CI 1.37-1.87) to 1.20 (95%CI 1.02-1.41) for Black women.

**Conclusion:** Much of the apparent differences in tumour characteristics by ethnicity are due to differences in age at presentation.

Acknowledgement: A version of this abstract has been published previously, see http://www.iarc-conference2016.com/build-preview.php?emailUser=toral.gathani%40ceu.ox.ac.uk&idUser=1317638&paramProjet=56329&type=projet for original and CC-BY license.

Disclosure: Funded by Macmillan Cancer Support

Corresponding author: Toral Gathani

## Cancer discovery and underpinning research

### **48. BAP1 loss induces BRCA1 dependent and independent defective spindle organization, checkpoint dysfunction and genomic instability**

#### Anita Singh^*1*^

##### ^*1*^*University of Leicester, UK*

**Background:** BRCA1 associated protein 1 (BAP1) is a tumour suppressor that is commonly inactivated in the majority of mesotheliomas. We have previously reported that loss of BRCA1 expression in mesothelioma is a common event, and is associated with resistance to spindle checkpoint activator vinorelbine, a drug with relevance to treatment of mesothelioma. However, 1. The mechanism of BRCA1 loss is unknown, and 2) the potential functional interaction between BRCA1 and BAP1 linked to spindle checkpoint is unknown. The aim of this study was to assess the functional relationship between BAP1 and BRCA1 and examine their role in genome stability in mesothelioma cells.

**Method:** We conducted functional genetic analysis of BAP1 and BRCA1 in two MPM cell lines, MSTO and H2452, the latter carrying an inactivating A95D mutation in the UCH domain of BAP1. BAP1 knockdown was achieved by siRNA transfection, while BRCA1 knockdown was achieved by doxycycline induction of an integrated shRNA.

**Results:** Loss of BAP1 expression led to reduced expression of BRCA1. Treatment with the proteasome inhibitor, MG132, restored BRCA1 expression in the absence of BAP1 indicating that BAP1contributes to post-translational stabilization of BRCA1 protein. Consistent with previous data, knockdown of BAP1 induced SAC deficiency and vinorelbine resistance concurrent with reduced expression of BRCA1. Loss of BAP1 and BRCA1 also led to an increased frequency of amplified centrosomes. Unexpectedly though, additional defects were observed in mitotic spindle architecture in response to BAP1 loss that were not seen upon loss of BRCA1.

**Conclusion:** Our data demonstrate that BAP1 controls BRCA1 expression through regulating its protein stability. They also demonstrate both BRCA1-dependent and independent roles for BAP1 in mitotic progression. These findings suggest that BAP1 loss may disrupt spindle checkpoint function and predict resistance to agents such as vinorelbine, a hypothesis that we will test in our randomised trial VIM.

Disclosure: Funded by University of Leicester

Corresponding author: Anita Singh

### **49. Glycolysis supports EGFR-mutant Lung Adenocarcinoma Cell Survival by blocking Autophagy-mediated EGFR Degradation**

#### Wonjun Ji^*1*^, Jin Kyung Rho^*1*^, Jaekyoung Son^*1*^, Jae Cheol Lee^*1*^, Chang-Min Choi^*1*^

##### ^*1*^*Asan Medical Center*

**Background:** Oncogenic epidermal growth factor receptor (EGFR) is essential for the development and growth of non-small cell lung cancer (NSCLC), but the precise roles of EGFR in lung cancer metabolism remain unclear.

**Method:** We studied the effect of EGFR on metabolism via targeted liquid chromatography-tandem mass spectrometry (LC-MS/MS) metabolomic analysis, glucose consumption and lactate production assay, and extracellular acidification and oxygen consumption assay in NSCLCs. Mechanisms regulating EGFR stability were investigated using RNA interference/pharmacological inhibitors followed by immunoblotting, cell death assay, and functional assays. Therapeutic potential of JNK activator was determined by monitoring cell growth and death and in mouse xenograft models. Immunohistochemistry was used to analyze JNK phosphorylayion and EGFR expression in NSCLC tissues (n = 244). All statistical tests were two-sided.

**Results:** EGFR knockdown in EGFR-mutant NSCLC significantly decreased the levels of glycolytic pathway intermediates (*p* < 0.05) via transcriptional regulation of glycolytic genes. EGFR-mutant NSCLCs exhibited significantly elevated glucose uptake and lactate production compared with EGFR-WT NSCLCs. Glucose deprivation markedly triggered EGFR-mutant NSCLC cell death through robust reduction of EGFR levels, but had no significant effects on EGFR-WT NSCLCs. EGFR-mediated enhanced glycolysis was a major source of carbon for TCA cycle in EGFR-mutant NSCLC, which is essential for maintaining EGFR levels. Glucose deprivation-mediated mitochondrial ATP depletion enhanced reactive oxygen species accumulation and subsequent JNK-mediated autophagy activation, which in turn induced EGFR degradation. The expression of phosphorylated JNK was significantly decreased in patients with EGFR-mutant NSCLCs (7.94%) compared with WT EGFR (23.28%) (*p* = 0.001). A reverse correlation between phosphorylated JNK and EGFR expression was observed in all tissues regardless of EGFR mutation (*p* = 0.0394).

**Conclusion:** These data suggest that enhanced glycolysis by EGFR mutation is required for maintaining EGFR levels through inhibition of JNK-induced autophagy, providing a promising rational for exploring JNK activators for patient bearing EGFR-mutation NSCLC.

Disclosure: None declared

Corresponding author: Wonjun Ji

### **50. Understanding the role of gamma-delta (γδ) T cells in pancreatic cancer**

#### Mark Lawrence^1^, Saadia Karim^1^, Jennifer P. Morton^*1*^, Seth B. Coffelt^1^

##### ^*1*^*Cancer Research UK Beatson Institute*

**Background:** Pancreatic ductal adenocarcinoma (PDAC) is a lethal malignancy with a 5-year survival rate of around 5%, where 80% of patients present with metastases on diagnosis. Metastasis is driven by inflammation, which potentiates the invasive and migratory behaviour of pancreatic cancer cells. Our lab is interested in a rare subset of immune cells known as gamma delta (gd) T cells that have pro-metastatic properties in breast cancer mouse models. Pro-metastatic gdT cells are defined by their ability to produce IL-17 and by the lack of expression of the costimulatory molecule CD27. IL-17-producing (CD27—) gdT cells promote breast cancer metastasis by expanding and polarising the neutrophils to an immunosuppressive phenotype, which inhibits anti-tumour CD8+ T cells. Additionally, recent reports have shown that gdT cells are crucial for pancreatic cancer progression in transplantable mouse models, and they constitute a large proportion of tumour-infiltrating lymphocytes within human PDAC. However, the mechanisms by which IL-17-producing (CD27—) gdT cells function are largely unknown.

**Method:** To investigate the mechanisms of gdT cell function, we use the Kras^G12D^;Trp53^R172H^;Pdx1-Cre (KPC) mouse model, which develops PDAC and liver metastasis at around 150 days of age.

**Results:** We have found that gdT cells are absent from normal pancreas, but relatively abundant in tumours from KPC mice. gdT cells are also reduced in livers from KPC mice when compared with livers from wild-type control mice.

**Conclusion:** Current efforts are underway to determine whether PDAC progression and metastasis are affected in gdT cell-deficient mice and to examine the potential crosstalk between gdT cells and neutrophils. These studies may uncover specific immunotherapeutic strategies to counteract metastatic pancreatic cancer.

Disclosure: Funded by Pancreatic Cancer UK Future Leaders Academy

Corresponding author: Mark Lawrence

### **51. Wnt/beta-catenin synergises with FOXG1 to drive exit from quiescence in neural stem cells, including glioblastoma stem cells**

#### Faye Robertson, Harry Bulstrode, Maria-Angeles Marques Torrejon, Eoghan O'Duibhir, Steve Pollard

##### *University of Edinburgh*

**Background:** Glioblastoma is a malignant brain tumour which is universally fatal. Stem cells within the tumour exist in a quiescent state, evade destruction and reactivate, causing relapse. These stem cells are known to overexpress the transcription factor FOXG1.

**Method:** We used an in vitro model of quiescence in mouse neural stem cells, incorporating a conditional human FOXG1 overexpression cassette, to identify, through high content pharmacological screening, a synergistic relationship between high FOXG1 expression and inhibition of glycogen synthase kinase 3 (GSK3) in driving cells into an active, proliferative state. We quantified this effect using EdU incorporation and colony forming assays. Wnt inhibitors were used to abrogate the effect and a genetic approach, using a constitutively active beta-catenin cassette, was used to elucidate the nature of the synergy. Patient-derived human glioblastoma stem cells (GSCs), with or without CRISPR-Cas9 excision of FOXG1, were used to confirm the relevance of the effect.

**Results:** EdU incorporation, following a 2 hour pulse, was increased from 4% in growth factors alone, 5.9% with FOXG1 overexpression, 5.5% with GSK3 inhibition, to 31.3% with FOXG1 overexpression and GSK3 inhibition combined. Colony forming assays confirm high efficiency cell cycle re-entry in cells with FOXG1 overexpression treated with a GSK3 inhibitor.

We subsequently show that this effect is present in patient-derived human GSCs and is abolished by excision of FOXG1.

The effect of GSK3 inhibition can be phenocopied both by a ligand of the canonical Wnt signalling pathway and by inducible constitutively active beta-catenin, suggesting that the synergy is effected through beta-catenin, the key downstream effector of canonical Wnt signalling.

Furthermore, the combined effect of FOXG1 overexpression and GSK3 inhibition on exit from quiescence can be abrogated by Wnt inhibitors.

**Conclusion:** Targeting the synergistic relationship between FOXG1 and beta-catenin may provide an exciting therapeutic opportunity in preventing relapse and improving the prognosis of glioblastoma.

Disclosure: Funded by Cancer Research UK

Corresponding author: Faye Robertson

### **52. Investigating Aspirin and Ticagrelor for the prevention of tumour cell-induced platelet aggregation**

#### Meera Chauhan, David Adlam, Anne Thomas, Alison Goodall, Joy Wright

##### *University of Leicester*

**Background:** Tumour cell induced platelet aggregation (TCIPA) may affect the metastatic potential of cancer. Mechanisms include protection of circulating tumour cells from immune destruction, interaction of platelet receptors with tumour ligands to facilitate adhesion, and enrichment of the tumour microenvironment, promoting extravasation and proliferation. Reducing these interactions using anti-platelet agents could alter metastatic progression. This study investigated the effects of Ticagrelor and Aspirin as monotherapy and dual therapy on TCIPA in metastatic breast cancer patients compared to healthy controls.

**Method:** Participants recruited to this randomised, crossover study received Aspirin or Ticagrelor for 2 weeks, followed by a 2-week washout and crossover to the other monotherapy, before completing 2 weeks of dual therapy. Platelet rich plasma was prepared from blood samples taken at baseline and the end of each treatment. Flow cytometry measured platelet activation markers (P-selectin expression, fibrinogen and Annexin-V binding) at rest and after agonist stimulation of the platelets in vitro.

**Results:** 20 healthy and 10 breast cancer participants completed the study. Fibrinogen binding was higher on resting platelets from breast cancer patients compared to healthy participants (49.5±7.8% vs 31.4±4.2%; p = 0.03). Ticagrelor reduced fibrinogen binding to platelets in healthy subjects to 17.9±3.6%(p = 0.008) and 26.7±5.2%(p = 0.048) in patients. Dual therapy reduced fibrinogen binding in breast cancer platelets to 31.3±6.8%(p = 0.008). Ticagrelor and dual therapy inhibited the response to platelet ADP stimulation in both populations, with reduced fibrinogen binding in comparison to untreated, stimulated platelets. (Healthy: Untreated 83.5±2.31% vs Ticagrelor 46.6±4.1%; p<0.0001, dual 48.4±4.5%; p<0.0001. Breast Cancer: Untreated 88.1±2.3% vs Ticagrelor 43.4±4.9%; p<0.0001, dual 58.1±7.9%; p = 0.0096)

**Conclusion:** Platelets from breast cancer patients are activated compared to healthy subjects when comparing levels of fibrinogen binding. Ticagrelor as monotherapy and dual therapy significantly reduces platelet activation in vivo and inhibits the platelet response to ADP. The pilot study indicates Ticagrelor may reduce TCIPA in breast cancer patients.

Disclosure: Funded by Astra Zeneca, ECMC, Hope Against Cancer

Corresponding author: Meera Chauhan

### **53. Regulation of IL-17-producing γδ T cells in breast cancer metastasis**

#### Sarah C. Edwards^1^, Anna Kilbey^2^, Robert Wiesheu^2^, Erin R. Morris^3^, Liam Hayman^2^, Damiano Rami^2^, Seth B. Coffelt^2^

##### ^*1*^*Beatson Institute for Cancer Research*, ^*2*^*Beatson Institute for Cancer Research. Institute of Cancer Sciences, University of Glasgow*, ^*3*^*Baker University, Baldwin City, KS, USA*

**Background:** In breast cancer, patients who develop stage IV metastatic disease have a median survival rate of 15%. Understanding the cellular mechanisms underpinning the progression of metastatic disease may present novel therapeutic interventions. In cancer, subsets of γδ T cells have been shown to present as anti-tumorigenic and pro-tumorigenic. In the *K14-Cre;Cdh1*^*F/F*^*;Trp53*^*F/F*^ (KEP) model of breast cancer, IL-17-producing γδ T cells promote lung metastasis by expanding neutrophils to suppress anti-tumorigenic CD8^+^ T cells. Selectively targeting IL-17-secreting γδ T cells in breast cancer metastasis may have therapeutic potential.

**Method:** To investigate which molecules govern the activation of pro-tumorigenic γδ T cells, we measured the expression of various activating receptors on CD27^+^ and CD27^—^ γδ T cells from tumour naïve or tumour-bearing mice, as CD27 stratifies IFNγ-producing (CD27^+^) from IL-17-producing (CD27^—^) γδ T cells.

**Results:** Surprisingly, we found that expression of NKG2D, a receptor involved in recognition of stressed or malignantly transformed cells is higher on CD27^—^ γδ T cells compared with CD27^+^ cells. This increased expression of NKG2D on CD27^—^ γδ T cells was specific, as other cytotoxic receptors were elevated on CD27^+^ γδ T cells. Furthermore, in the *K14-Cre;Brca1*^*F/F*^*;Trp53*^*F/F*^ (KB1P) model of breast cancer, we found that NKG2D ligands are upregulated in the myeloid compartment at metastatic sites, such as the lung of tumour-bearing mice.

**Conclusion:** We are currently investigating the interplay between these NKG2D ligand-expressing myeloid cells and IL-17-producing γδ T cells to determine whether the NKG2D axis plays a role in breast cancer metastasis.

Disclosure: Funded by Beatson Institute for Cancer Research

Corresponding author: Sarah C. Edwards

### **54. Specific features of DNA methylome in Glioblastoma Multiforme**

#### Maria Eleftheriou^1^

##### ^1^*University of Nottingham*

**Background:** DNA methylation (5-methylcytosine, 5mC) is the major epigenetic modification involved in transcriptional regulation during the early stages of development in eukaryotes. The patterns of 5mC are frequently altered in cancer. TET proteins can enzymatically oxidize 5mC producing 5-hydroxymethylcytosine (5hmC), 5-formylcytosine (5fC) and 5-carboxylcytosine (5caC). Although the exact biological roles of these oxidized forms of 5mC in cancer pathogenesis are still unknown, there are indications that they might contribute to malignant transformation. According to several reports, 5hmC levels are reduced in human tumours; however, the distribution of 5fC and 5caC in cancers is poorly studied. Although our previous studies showed that 5caC is surprisingly enriched in a proportion of breast cancers and pediatric brain tumors, the distribution and the biological role of this mark in glioblastoma multiforme (GBM) has not been systematically assessed.

**Method:** Here, using mass spectrometry and immunofluorescence, we examine the global levels of 5hmC and 5caC in four human GBM cell lines (LN18, LN228, U251 and U87MG).

**Results:** We show that while the GBM cell lines exhibit low levels of 5hmC, they are, rather unexpectedly, characterized by relatively high immunochemistry and mass-spec detectable 5caC levels paralleled by the absence of 5fC. Remarkably, 5caC content in GBM does not correlate with 5hmC levels but corresponds to elevated levels of TET2 transcript in these cancers where its transient siRNA mediated knockdown leads to a dramatic decrease in 5caC levels.

**Conclusion:** Our data reveals the unique epigenetic signature of GBM contributing to potential development of novel approaches for diagnosis and therapy of these tumours.

Disclosure: Funded by University of Nottingham

Corresponding author: Maria Eleftheriou

### **55. Investigating the Interplay between Wilms' Tumour 1 Protein and DNA Demethylation in Brain Tumours**

#### Ashley Ramsawhook^1^

##### ^1^*University of Nottingham*

**Background:** 5-methylcytosine (5mC) is an epigenetic modification usually associated with transcriptional repression. The Ten–Eleven Translocase proteins (Tet1/2/3) can oxidise 5mC (oximC) to 5-hydroxymethylcytosine (5hmC), 5-formylcytosie (5fC) and 5-carboxylcytosine (5caC) in vertebrate DNA. These oxidised forms of 5-methylcytosine exhibit distinct genomic distributions in different biological systems. Whilst embryonic stem cells exhibit elevated levels of oxi-mC modifications, adult somatic tissue genomes display depletion for 5fC and 5caC modifications. Curiously, neoplasms of the central nervous system, particularly ependymoma, medulloblastoma and glioblastoma possess significant enrichment of these oxidised forms, however the precise functional roles of these modifications are not fully understood. Transcription factor and tumour suppressor/oncogenic protein Wilm’s Tumour 1 (*WT1*) possesses preferential binding affinity for 5mC and 5caC over unmodified cytosines, 5hmC and 5fC in vitro and exhibits elevated expression in multiple cancers. Expression of WT1 in embryonic neural epithelial progenitors during lineage commitment but absence in terminally differentiated neurons and astrocytes may be indicative of aberrant developmental signalling resulting in malignant neoplasm formation.

**Method:** By employing mass spectrometry-validated immunohistochemistry and confocal microscopic quantification, we demonstrate that high levels of both 5caC and WT1 are characteristics of brain tumour cell lines. Moreover, 5caC is present on promoters of core brain tumour signalling pathway genes. Furthermore, we endeavour to perform CRISPR cas9 facilitated *WT1* targeted knockout and subsequently implement next generation sequencing technologies to globally map 5caC genomic distribution and influence on brain tumour transcriptomes.

**Results:** Ultimately this will facilitate the elucidation of a mechanistic relationship between WT1 and 5caC which may aetiologically induce or functionally perpetuate clinical aspects of brain tumour pathogenicity.

**Conclusion:** Our findings may gain novel insights on the biological roles of oxidised forms of 5mC and, potentially, highlight deleterious developmental origins of lethal brain tumours.

Disclosure: Funded by The Biotechnology and Biological Sciences Research Council

Corresponding author: Ashley Ramsawhook

### **56. Molecular mechanisms underlying phenotypic plasticity in malignant glioma**

#### Costanza Lo Cascio^1^, Ernesto Luna, Rohit Khurana^1^, Roberto Fiorelli, Shwetal Mehta^1^

##### ^1^*Barrow Neurological Institute*

**Background:** Glioblastoma (GBM) is characterized by rapidly proliferating and invasive cells that infiltrate normal brain regions. Following exposure to aggressive treatment regimens, GBMs frequently shift their biological features upon recurrence, acquiring a more resistant phenotype. However, the dynamics and molecular mechanisms that facilitate GBM recurrence are still poorly understood. Considering the unchanged dismal prognosis for GBM patients, there is a need to understand, at a systems level, how plastic processes (molecular switches) in glioma stem-like cells (GSCs) may drive tumor maintenance and cancer cell adaptability in GBM. The objective our study was to determine how GSCs temporally adjust their expression profile and phenotype in response to ionizing radiation in vitro and in vivo using patient-derived xenograft (PDX) models of GBM.

**Method:** We established PDX GBM models by intracranially implanting two patient-derived GSC lines belonging to different GBM molecular subgroups into immunocompromised mice. The tumor-bearing mice were treated with single doses of ionizing radiation to assess acute responses to treatment. Mice from each cohort were be sacrificed at multiple distinct time points following treatment. Using immunohistochemical methods, we assessed changes in the expression of GBM subclass markers, stemness and differentiation markers, and DNA damage/repair proteins across the entire tumor population over time. To understand how GSCs respond to radiation at a molecular level, we employed mass cytometry (CyTOF) and RNA-seq to determine how important cellular signaling pathways and transcriptional programs necessary for GSC self-renewal, invasion and growth are altered at various time points post-treatment.

**Results:** We demonstrate that GSCs, both in vitro and in vivo, undergo an immediate response following exposure to radiation that results in a global modulation of the expression of key stemness and proliferation genes under adverse conditions.

**Conclusion:** Our results suggest that this acute response allows GSCs to enter a transient semi-differentiated state that favors GSC adaptability and resistance to therapy

Disclosure: Funded by National Institutes of Health

Corresponding author: Costanza Lo Cascio

### **57. Identification of C1orf106 as a TGF-β target gene which promotes clonogenicity and anchorage-independent growth**

#### Lauren Strathearn^1^, Susan Mason^2^, Lindsay Spender^1^, Karen Blyth^2^, Gareth Inman^1^

##### ^*1*^*University of Dundee*, ^*2*^*Beatson Institute for Cancer Research*

**Background:** The dual and opposing roles of Transforming Growth Factor-β signalling (TGF-β) in tumorigenesis are well recognized. However the exact mechanisms behind this switch in biology from tumour suppressive to tumour promoting are complex and lesser understood. Microarray analysis identified the uncharacterised open reading frame C1orf106 as a novel TGF-β target gene required for TGF-β induced anchorage-independent growth of vulval squamous cell carcinoma cells. Here we further investigate TGF-β regulation of C1orf106 and its potential role in tumorigenesis.

**Method:** C1orf106 gene and protein expression was assayed by qRT-PCR and western blotting following exogenous stimulation or inhibition of TGF-β signalling. The role of the canonical TGF-β pathway in regulating C1orf106 transcriptional induction was explored by siRNA-mediated gene silencing. C1orf106 mRNA expression levels and influence on clinical outcomes were assessed in the Oncomine™ and KM Plotter platforms. The phenotypic effects of stable overexpression or shRNA mediated knockdown of C1orf106 expression was investigated in breast cancer cell lines. Anchorage-independent growth and colony-forming assays, and a limiting dilution xenograft were carried out to assess tumourigenicity in vitro and in vivo, respectively.

**Results:** C1orf106 mRNA and protein induction in response to TGF-β stimulation was observed in a plethora of cell types and determined to be SMAD3-dependent. Analysis of publically available datasets indicated that elevated C1orf106 mRNA expression is associated with poor clinical outcomes in breast cancer. C1orf106 expression correlated with metastatic progression in the 4T1 murine mammary carcinoma model. High C1orf106 was associated with enhanced anchorage-independent growth ability, colony forming capacity and clonogenicity in vitro in this model system and trended towards increased tumour initiation frequency in vivo.

**Conclusion:** We have identified C1orf106 as a novel and robust TGF-β target gene correlating with poor prognosis in breast cancer and shown it to enhance tumourigenicity in a series of breast cancer cell lines.

Disclosure: Funded by Cancer Research UK

Corresponding author: Lauren Strathearn

### **58. Defining microRNA mediated regulation of CD157 involved in colon cancer progression**

#### Mahnaz Darvish Damavandi^1^, George Vlachogiannis^2^, Gift Nyamundanda^2^, Andrea Lampis^2^, Harold Parkes^3^, Somaieh Hedayat^2^, Jens Hahne^2^, Matteo Fassan^4^, Anguraj Sadanandam^2^, Owen Sansom^5^, Nicola Valeri^2^

##### ^*1*^*Division of Molecular Pathology, The Institute of Cancer Research, London*, ^*2*^*The Institute of Cancer Research*, ^*3*^*Division of Radiotherapy and Imaging, The Institute of Cancer Research, London*, ^*4*^*Department of Medicine DIMED, University of Padova, Padova*, ^*5*^*Cancer Research UK Beatson Institute, Glasgow*

**Background:** Progressive accumulation of mutations in oncogenic and tumour suppressor pathways (e.g., KRAS and p53) and also microRNA (miR) deregulation are associated with colorectal cancer (CRC) development. Very little has been done to dissect specific CRC pathways involved in miR regulation in response to stress and metabolic changes. Therefore, we investigated the interaction between miR-mediated regulation of bone marrow stromal cell antigen 1 (BST1) or CD157, a metabolic enzyme involved in the conversion of nicotinamide adenine dinuclease (NAD) to paracrine factor cyclic ADP ribose (cADPR), following the acquisition of KRAS mutation in metastatic CRC.

**Method:** A combination of array analysis data, in-silico prediction tools and nuclear magnetic resonance (NMR) system were used to analyse gene (mRNA) and miR expression, as well as metabolic changes associated with different rounds of knock in/out mutations in Apc, Kras, and p53 CRC mouse models and their tumour-derived organoids (TDOs). Subsequently, we genetically modified human CRC cell lines and patients KRAS mutant (mut) TDOs from CRC metastases to modulate the candidate gene and miR. Organoids formation, growth rate and viability were measured with Live-Cell imaging systems.

**Results:** We identified mRNA/miR networks involved in cancer metabolic pathways and assessed the most significant candidates. Further target validation and analysis of human tissues microarrays showed an association between KRAS mutations, miR-203 down-regulation and over-expression of BST1, which was identified as a direct target of miR-203 regulation in our studies. Repressing BST1 and over-expressing miR-203 had a significant effect on the proliferation and migration abilities of organoids in 3D culture in normal and calorie-deprived conditions.

**Conclusion:** This project aimed to find a promising candidate as a therapeutic target in KRAS mut CRC. Although we showed KRAS mut CRC cells lost a growth advantage with miR-203 and BST1 deregulation ex vivo, work is on going to confirm the consequence of their interaction in vivo.

Acknowledgement: A version of this abstract has been published previously, see PO346 https://esmoopen.bmj.com/content/3/Suppl_2/A364.1 For original and CC-BY license.

Disclosure: Funded by The Institute of Cancer Research

Corresponding author: Nicola Valeri

### **59. Phenotypic subtyping of matched primary colonic tumours and liver metastases**

#### Kathryn Pennel^1^, Arfon Powell^2^, Donald McMillan^1^, Paul Horgan^1^, Antonia Roseweir^1^, Joanne Edwards^1^

##### ^*1*^*University of Glasgow*, ^*2*^*Cardif University*

**Background:** Colorectal cancer (CRC) is a heterogeneous group of malignancies that arise in the same organ. The 5-year survival rate for CRC is 60% and this is significantly reduced in stage four metastatic disease. A common site of metastasis is the liver. In 2017, phenotypic subtypes for CRC were developed in an effort to move towards precision medicine. The phenotypic subtypes (immune, canonical, latent and stromal) are derived from three features; inflammation, stromal invasion and proliferation. This study aimed to investigate the relationship between primary tumour and liver metastases phenotypic subtypes and association with clinicopathological outcomes.

**Method:**  Matched patient-derived colonic primary tumours and liver metastases were stained for Ki67 proliferation index and an H&E analysed for Klintrup-Makinen grade and tumour-stroma-percentage to determine phenotypic subtype. The relationship between the primary tumour subtype (PS-primary) and liver metastases (PS-met) was investigated using bivariate correlations and paired sample T-tests. The relationship between PS-primary/PS-met and clinicopathological features was analysed using Chi-squared tests.

**Results:** Phenotypic subtype was observed to correlate between the primary tumour and metastasis (n = 42, p = 0.001). In the primary tumours, immune subtype associated with age (p = 0.047) and nuclear HIF1a (p = 0.043); canonical subtype associated with cytoplasmic MMP9 expression (p = 0.015); latent subtype associated with marginal involvement (p = 0.013); and stromal subtype associated with modified Glasgow Prognostic Score (p = 0.044) and cytoplasmic HIF1a expression (p = 0.012). In the metastases, immune subtype associated with age (p = 0.01) and albumin levels (p = 0.043); latent subtype associated with cytoplasmic MMP9 expression (p = 0.031); and stromal subtype associated with cytoplasmic CRP expression (p = 0.032). However, the canonical subtype showed no significant association.

**Conclusion:**  The results suggest that PS-primary is predictive of PS-met, and that each phenotypic subtype associates with different specific clinicopathological features differing between primary or metastatic lesions. This may provide a step towards the development of precision medicine for CRC.

Disclosure: Funded by Medical Research Council

Corresponding author: Kathryn Pennel

### **60. A novel cis-acting lncRNA controls HMGA1 expression and is deregulated in non-small cell lung cancer**

#### Greg Stewart, Adam Sage, Katey Enfield, Erin Marshall, Victor Martinez, Wan Lam

##### *BC Cancer Research Centre*

**Background:** High mobility group A1 (HMGA1) is aberrantly expressed in several aggressive cancer types, including non-small cell lung cancer (NSCLC), where high HMGA1 expression has been associated with poor survival and chemotherapy resistance. While HMGA1 is known to be deregulated in lung cancer, the mechanisms that mediate its expression remain unknown. Since their discovery, long non-coding RNAs (lncRNAs) have been increasingly implicated in cancer-associated phenotypes. Recently, some lncRNAs have been shown to regulate the expression of neighbouring protein-coding genes, including oncogenes and tumour suppressor genes. These lncRNAs, known as *cis*-acting, may represent undiscovered therapeutic action points in cancer driving pathways. Here we investigate the deregulation of a putative *cis*-acting lncRNA in NSCLC, and its relationship with the oncogene HMGA1.

**Method:** LncRNA expression was generated from RNA-sequencing data from 36 microdissected tumour and matched non-malignant tissues. Normalized sequence read counts were used to identify transcripts with significantly deregulated expression (Wilcoxon Signed-Rank Test, BH-p<0.05). Validation was performed in sequencing data obtained from The Cancer Genome Atlas (TCGA). SiRNA-mediated knockdown of lncRNA candidates was performed in a non-malignant epithelial lung cell line (BEAS-2B). Quantitative real-time PCR was used to observe the effects of lncRNA knockdown on the expression of neighbouring protein-coding genes.

**Results:** Our analyses identified *RP11.513I15.6*, an undescribed lncRNA neighbouring *HMGA1*, to be significantly downregulated in 2 cohorts of LUAD samples. *HMGA1* expression was found to be anticorrelated with *RP11.513I15.6*, as tumours with downregulated *RP11.513I15.6* displayed significant overexpression of *HMGA1*. In vitro experiments demonstrated siRNA-mediated inhibition of *RP11.513I15.6* in immortalized lung epithelial cells resulted in a significant increase in HMGA1 expression.

**Conclusion:** Our results suggest that *RP11.513I15.6* is a novel *cis*-acting lncRNA that negatively regulates HMGA1, and may contribute mechanistically to the maintenance of lung cancer phenotypes. Further characterization of this oncogenic regulatory mechanism may uncover a novel therapeutic intervention point for tumours driven by HMGA1.

Disclosure: Funded by Canadian Institutes for Health Research

Corresponding author: Greg Stewart

### **61. Regulatory heterogeneity in glioblastoma multiforme informs novel drug target discovery**

#### Yunpeng Liu^1^, Ning Shi^2^, Shan He^2^, Michael Hemann^1^, Aviv Regev^1^

##### ^*1*^*Massachusetts Institute of Technology*, ^*2*^*University of Birmingham*

**Background:** Glioblastoma multiforme (GBM) is one of the most malignant forms of cancer. Bulk and single-cell transcriptome profiling have revealed high levels of both inter- and intratumour heterogeneity in GBM. The disease has been stratified into four molecular subtypes according to gene expression - Classical, Neural, Proneural and Mesenchymal, each of which exhibiting distinct mutational signatures and therapeutic responses. However, the underlying regulatory circuitry that gave rise to such heterogeneity and its implications for rational design of therapy are unclear.

**Method:** We have developed a nonlinear regression model to understand key regulatory networks across the four GBM subtypes. We first constructed a backbone network of transcription factor (TF) - target gene pairs inferred from chromatin landscape data and TF binding motifs, and applied nonlinear regression using expression profiles of each subtype to derive subtype-specific regulatory parameters for each TF-gene pair. Next, we mined for co-regulatory TF pairs using correlation analysis. Mechanisms responsible for subtype-specific behaviour of TFs were then inferred from expression, regulatory and co-regulatory signatures. Finally, we simulated the effects of perturbing druggable signature TFs and their partners by propagating changes in expression to the corresponding target genes and then to the protein signaling layer using a random walk-based algorithm.

**Results:** We show that subtype-specific repurposing of TFs explains a significant proportion of subtype-specific transcription landscapes. At least two mechanisms for TF repurposing are implied - differential expression of the TF itself and differential partnering with co-regulatory TFs. Using effectors of the apoptosis pathway as readout in our in silico perturbation analysis, we show that targeting a subset of druggable signature TFs and/or their partners may be specifically beneficial for treating the corresponding subtypes of GBM.

**Conclusion:**  Data-driven modeling of transcription regulation in GBM is capable of gaining new biological insight into the molecular underpinnings of its heterogeneity and aids rational design of subtype-specific targeted therapy.

Disclosure: Funded by Ludwig Cancer Research

Corresponding author: Yunpeng Liu

### **62. An Effective Pipeline for Whole Genome Sequencing for Research in a Tertiary Cancer Centre**

#### George Morrissey^1^, Alison Berner^2^, Tracy Odigie^1^, Alice Rendall^1^, Keith Rogerson^1^, Rahul Kurup^1^, Glen Brice^1^, Helen Hanson^1^, John Short^1^, Katie Snape^1^, Ruth Pettengel^1^, Nirupa Murugaesu^2^

##### ^*1*^*St George's University Hospital NHS Trust*, ^*2*^*Genomics England*

**Background:** The 100,000 Genomes Project is performing whole genome sequencing (WGS) on paired tumour and germline DNA from patients with cancer. It aims to provide genomic data to inform patient management, contribute to a database for future research, and establish new diagnostic pathways in histopathology and genetics. We describe the experience of St Georges University Hospital, part of South London Genomic Medicine Centre.

**Method:** Patients are identified as eligible by screening of biopsy and theatre lists and multidisciplinary team meetings (MDTs). After liaising with the clinical team and histopathology, a fresh tumour sample is obtained. Patients are approached prospectively or retrospectively, for consent and blood or saliva for germline testing. If adequate DNA is extracted, it is sent to Illumina for sequencing and Genomics England (GEL) for annotation. Resulting genomic reports provide detailed information on somatic and germline single nucleotide variants, copy number and mutational signatures. These are discussed at a Genomic MDT consisting of Consultant Oncologist, Consultant Clinical Geneticist and Consultant Clinical Scientist. A summary report of actionable or potentially actionable findings is produced and sent to the referring clinician.

**Results:** From 1st June 2016 to 11th May 2018, we collected 873 samples, 324 biopsies and 549 resections. 1060 patients were consented, and 556 patients had DNA extracted. To date, 492 cases have been submitted to GEL, of which 121 have been discussed at MDT and had results returned.

57 patients (47%) had clinically actionable or potentially actionable mutations. The most frequently occurring were mutations in KRAS and PIK3CA, each found in 16.5% of patients. Two patients had germline findings, one in BRCA2 and one in MSH2.

**Conclusion:** We demonstrate an effective pipeline for tumour WGS and review of results. Our data suggests that WGS has the potential to affect patient management and increase recruitment to trials.

Disclosure: Funded by Genomics England, St George's University Hospital NHS Trust

Corresponding author: George Morrissey

### **63. eIF4A2 is a regulator of hypoxic translation and colorectal tumour cell survival**

#### Hannah Bolland^1^, Alan McIntyre^1^, Abdol Shams-Nateri^1^, Cleo Bishop^2^, Andrew Silver^2^

##### ^*1*^*University of Nottingham*, ^*2*^*Blizzard Institute Barts and the London School of Medicine and Dentistry*

**Background:**  Colorectal cancer kills more than half a million people a year worldwide. Hypoxia (low oxygen) is associated with increased chemotherapy and radiotherapy resistance. 1 in 3 colorectal tumours contain regions of low oxygen. Under hypoxic conditions components of the protein synthesis machinery undergo an adaptive change as do the translational efficiencies of a subset of pro-survival mRNAs. eIF4A2, an RNA helicase is differentially expressed in a variety of cancers. We hypothesised that eIF4A2 played a role in normoxic and hypoxic colon tumour cell survival through its regulation of proteins required for hypoxic survival.

**Method:** Using a panel of colorectal cancer cell lines we assessed hypoxic regulation of eIF4A2 and its role in survival and cell viability in 2D and 3D spheroid cultures and in on-going in vivo experiments using inducible shRNA eIF4A2 knockdowns. RNA-seq was used to identify eIF4A2 regulated transcripts.

**Results:** eIF4A2 was significantly upregulated by hypoxia at the protein and RNA level under control of HIF1α. Knockdown of eIF4A2 significantly reduced cell survival and proliferation in 2D and 3D spheroid cultures, and we identified increased apoptosis and necrosis in the hypoxic core of the spheroids. RIP-seq identified a number of key hypoxia pro-survival proteins are eIF4A2 dependently translated.

**Conclusion:** eIF4A2 is required for hypoxic tumour cell survival in colorectal cancer. This highlights the value of the development of small molecule inhibitors that specifically target eIF4A2 to target the therapy resistant hypoxic tumour microenvironment.

Disclosure: Funded by Bowel and Cancer Research

Corresponding author: Hannah Bolland

### **64. Long non-coding RNA LINC00973 is a putative biomarker of colon cancer relapse**

#### Nikolai Lisitsyn, Olga Zinovieva, Evgenia Grineva, Maria Prokofjeva, Dmitry Karpov, Andrei Zheltukhin, George Krasnov, Anastasiya Snezhkina, Anna Kudryavtseva, Peter Chumakov, Tamara Mashkova, Vladimir Prassolov

##### *Engelhardt Institute of Molecular Biology*

**Background:** Early prediction of tumor relapse depends on identification of novel prognostic cancer biomarkers, which are suitable for monitoring of the response of various cancer types to the action of chemotherapeutic drugs.

**Method:** RNA-sequencing (RNA-Seq), RT-qPCR, and bioinformatics, combined with the study utilizing murine tumour xenograft model.

**Results:** We have found most significant (up to 100-fold) and consistent changes in the abundance of LINC00973 upon treatment of HT-29 and HCT-116 colon cancer cells with 5-fluorouracil, oxaliplatin, and irinotecan at different doses and durations, both in vitro and in vivo. Though the function of LINC00973 has not yet been determined, the RAID v2.0 database suggests that this RNA may form a complex with lncRNA CRNDE that is a part of the Polycomb repressive complex 2. This complex epigenetically silences DUSP/CDKN1A expression, promoting proliferation and suppressing apoptosis of colon cancer cells. Using CRISPR/Cas9 system we knocked out the LINC00973 encoding gene in HT-29 cells and found significant decrease in their proliferation and activation of apoptosis, as compared to intact controls. Besides, LINC00973 KO cells were much less resistant to chemotherapeutic drugs, which imply that overexpression of this RNA is a key event in the acquisition of chemoresistance by colon cancer cells. In accordance with these data, bioinformatics search revealed that expression of LINC00973 is drastically reduced in most types of carcinomas, but even small increase in LINC00973 abundance in most aggressive subtypes of colon cancer results in sharp increase in the probability of tumour relapse (hazard rate=1.34). Prognostic value of LINC00973 was also demonstrated for kidney and head and neck cancers.

**Conclusion:** LINC00973 is a perspective biomarker for prognosis of several cancer types. Further clinical studies are necessary in order to determine, if measurement of the LINC00973 abundance throughout the course of neoadjuvant therapy might be useful for the reliable prediction of colon cancer relapse.

Disclosure: The Russian Science Foundation

Corresponding author: Nikolai Lisitsyn

### **65. Nuclear morphometry features distinguish cell types and outcome groupings in lung cancer**

#### Katey Enfield^1^, Spencer Martin^1^, Erin Marshall^1^, Zhaolin Xu^2^, Martial Guillaud^1^, Calum MacAulay^1^, Wan Lam^1^

##### ^*1*^*British Columbia Cancer Research Centre*, ^*2*^*Dalhousie University*

**Background:** The shape and organization of tumour cell nuclei has been shown to be associated with aggressiveness in several cancers, including prostate and cervical cancer. Fortunately, this data can be obtained using a single quantitative nuclear stain on FFPE tissues. We sought to determine whether nuclear features could be utilized to distinguish cell types within the lung tumour microenvironment and to classify outcome groupings.

**Method:** A tissue microarray of non-small cell lung cancer samples was stained with the quantitative nuclear dye, Feulgen/thionin. The stained slides were scanned using a hyperspectral imaging platform, images were spectrally unmixed, and cell nuclei were identified using a segmentation algorithm. Nuclei were then analyzed to identify 245 nuclear morphometry features. Nuclear features were assessed in order to dichotomize good outcome (alive at 5 years) and bad outcome (survival of 3 years or less) patients, as well as several cell types within the tumour microenvironment. Finally, we assessed the spatial organization of these different classes of nuclei in good and poor outcome cases.

**Results:** Nuclear features derived from thionin stain classified epithelial, stromal, and immune cells. Of the 245 nuclear features assessed, 87 differed significantly between good and poor outcome groups. While many distinguishing features were identified in tumour cell nuclei, we were also able to identify distinguishing features of non-tumour cell nuclei. Using pairs of features, good and poor outcome cases were stratified with up to 69% accuracy. However, the addition of spatial information to our classifier resulted in the improvement of sample stratification to 86% accuracy.

**Conclusion:** Using a single nuclear stain on FFPE lung cancer histology specimens, we were not only able to sub-classify cell types, but also to distinguish between good and poor outcome cases. The spatial organization of these cell types greatly improved our classifier, indicating the potential of this approach for prognostic purposes.

Disclosure: Funded by NSA, CIHR

Corresponding author: Katey Enfield

### **66. Identification and targeting of metabolic vulnerabilities in leukaemic stem cells using integrated omic approach**

#### Vignir Helgason^1^, Zuzana Brabcova^1^, Kevin Rattigan^1^, Lisa Hopcroft^1^, Eyal Gottlieb^2^, Alexei Vazquez^3^

##### ^*1*^*University of Glasgow*, ^*2*^*Technion Integrated Cancer Center*, ^*3*^*Cancer Research UK, Beatson Institute*

**Background:** Leukaemia progression and relapse are fuelled by leukaemic stem cells (LSCs) that are resistant to current treatments. Therefore, identification of targetable pathways that selectively maintain LSC survival is critical. Metabolic reprogramming is a core feature of cancer cells making them susceptible to manipulation in a selective manner. We aimed in this study is to identify and target metabolic dependencies in LSCs, using primitive primary cells isolated from individuals with chronic myeloid leukaemia (CML) and healthy donors.

**Method:** We have recently developed improved protocols for metabolic flux assays in patient-derived LSCs, where heavy isotope-labelled carbons (^13^C), are used to detect different isotopes of intracellular metabolites over time, using a state-of-the-art liquid chromatography-mass spectrometry system (Kuntz *et al*. Nat Med, 2017). To fully understand the metabolic properties of LSCs, we have extended our work and combined functional studies with integrative-omics and data-driven computational approach to assess the metabolic changes from transcriptomic and metabolomics datasets.

**Results:** We show that LSCs display an increase in fatty acid oxidation (FAO) and flux of glucose carbons through the tricarboxylic acid (TCA) cycle, along with an elevated mitochondrial respiration, when compared with normal hematopoietic stem cells (HSCs). Integrative omic approach confirmed upregulation of FAO and the TCA cycle in LSCs. Additionally, increased activity of pyruvate carboxylase, together with increase in ^13^C enrichment in TCA cycle metabolites, indicate a significance of pyruvate anaplerosis and reductive carboxylation, when comparing LSCs to HSCs. This suggests that LSCs may be selectively sensitive to inhibition of mitochondrial oxidative phosphorylation (OXPHOS). Of clinical significance, we show that inhibition of mitochondrial translation reduces this aberrant oxidative metabolism and induces death in LSCs in vitro and in a robust pre-clinical patient-derived xenograft model.

**Conclusion:** We conclude that LSCs are dependent on anaplerosis and mitochondrial OXPHOS for their survival and inhibition of mitochondrial metabolism selectively targets drug-resistant CML LSCs.

Disclosure: Funded by Cancer Research UK; KKLF; Leuka; The Howat Foundation

Corresponding author: Vignir Helgason

### **67. Identifying breast-to-brain metastasis-associated gene mutations by whole exome sequencing**

#### Mark Morris^1^, Ivonne Olivares^1^, Timothy Dawson^2^, Kate Ashton^2^, Charles Davis^2^, Michael Jenkinson^3^, Andrew Brodbelt^3^, Angel Armesilla^1^, Tracy Warr^1^

##### ^*1*^*University of Wolverhampton*, ^*2*^*Royal Preston Hospital*, ^*3*^*The Walton centre*

**Background:** Over recent years breast cancer survival rates have improved. However, even after many years of apparent disease-free health, cancer can recur. Many of these tumours occur specifically in the brain and metastatic brain tumours have very poor prognoses. Identifying genomic alterations that occur in breast primary tumours that eventually metastesise to the brain will provide new opportunities for treatment and prognosis.

**Method:** Whole-exome sequencing (WES) was carried out in 18 brain metastasis samples that originated from breast tumours. Each sample was sequenced to a depth >100X. Bioinformatic analysis was carried out to identify common recurrent mutations. We are in the process of validating the candidate mutations by Sanger sequencing and screening a larger cohort of Breast to Brain Metastasis (BBMs) and non-metastatic primary breast tumours to confirm metastasis-associated alterations.

**Results:** Each of the 18 BBMs analysed by WES contain >7000 non-synonymous variants. All variants were screened for their consequence on the protein product (via Polyphen and the Exome Aggregation Consortium(ExAc)). Those variants with high scores relating to pathogenicity were retained. Following this filtering, potential germline polymorphisms were excluded by removing those variants with a minor allele frequency (MAF) of >0.1%. This screening has generated a long list of 300 variants found across all 18 tumours analysed. A final screen identified genes that contained pathogenic variant in more than 2/18 tumours and had been described in any other cancer type (via the catalogue of somatic mutations (COSMIC)).

Via this stringent screen, we have identified 22 candidate metastasis-associated genes. we are currently screening non-metastatic primary breast tumours and BBM tumours to determine how frequently these occur. The genes identified have varied cellular roles, including cell surface proteins, migration and gene regulation.

**Conclusion:** We expect that this analysis will identify genes that are frequently mutated in BBMs, but infrequently in non-metastatic breast tumours.

Disclosure: Funded by University of Wolverhampton

Corresponding author: Mark Morris

### **68. The role of translational elongation loss in colorectal cancer**

#### Nikola Vlahov^1^, John Knight^1^, Owen Sansom^1^

##### ^*1*^*The Beatson Institute for Cancer Research*

**Background:** The elongation phase of mRNA translation is the most energy and amino acid consuming part of protein synthesis. This process is controlled by the Eukaryotic elongation factor 2 kinase (eEF2K), an atypical calmodulin-dependent protein kinase that slows down translation elongation by phosphorylating eEF2. eEF2K functions as a negative regulator of protein synthesis and cell growth. Cancer cells may possess mechanisms to inhibit eEF2K. We have previously shown that in colorectal cancer Rapamycin-induced reduction of elongation suppresses intestinal regeneration and tumourigenesis following APC loss. This process was shown to be controlled by eEF2K.

**Method:** We generated in vivo mouse models with acute deletion of either a single or both copies of *Apc* with either conditional expression of eEF2K kinase dead allele (D273A) or conditional deletion of eEF2K in the mouse intestine. We followed the rates of intestinal proliferation and tumourigenesis and the response to various drug therapies.

**Results:** In both of these models we observed loss of the inhibitory phosphorylation of eEF2. Intestinal crypt organoids from these mice showed increased ribosome run-off rate associated with inhibition of elongation control due to the loss of eEF2K function. We have also demonstrated that loss of eEF2K function or total eEF2K levels, following *Apc* loss, led to significant increase in survival compared to mice with *Apc* deletion alone. Deletion of eEF2K in addition to Apc and *Kras*, one of the most frequently altered genes following *Apc*, did not have an effect on survival.

**Conclusion:** We found that eEF2K deletion increases survival in mice that have lost *Apc* and we have explored the possibility to combine eEF2K inhibition with conventional drug treatments for colorectal cancer.

Disclosure: Funded by Wellcome Trust

Corresponding author: Nikola Vlahov

### **69. Single cell copy number variation analysis (CNV) of circulating tumour cells (CTCs) in neuroendocrine tumour (NET) patients**

#### Alexa Childs^1^, Clare Vesely^1^, Francesca Rizzo^1^, Leah Ensell^1^, Helen Lowe^1^, Christina Thirlwell^1^, Christos Toumpanakis^2^, Martyn Caplin^2^, John Hartley^1^, Tim Meyer^1^

##### ^*1*^*University College London Cancer Institute*, ^*2*^*Royal Free Hospital, London*

**Background:** The identification and characterisation of CTCs as part of a minimally invasive “liquid biopsy” provides an opportunity to explore NET biology, identify therapeutic targets and investigate tumour heterogeneity. Here we report the first genomic analysis of NET CTCs at the single cell level.

**Method:** Peripheral blood samples from metastatic neuroendocrine patients were enriched for CTCs using the EpCAM-dependent CellSearch and EpCAM-independent, size-based Parsortix platforms. Enriched samples were fluorescently labelled with DAPI, CK, +/- EpCAM and CD45 antibodies to enable identification and recovery of single CTCs and white blood cells (WBC) using DEPArray technology. Whole genome amplification was performed on individual cells, followed by quality-control PCR to assess amplified DNA quality. Low pass whole genome sequencing was undertaken and the CNV profile of CTCs analysed to identify chromosomal gains and losses. WBC were used as controls.

**Results:** CNV profile analysis revealed copy number alterations among CTCs and flat profiles for WBC. CTCs derived from a midgut patient showed distinct CNV profiles in keeping with genomic heterogeneity, despite conserved gains on chromosome 7 and 16, corresponding to gains in CDK6, MET and BRAF. Hierarchical clustering of CTCs derived from a renal NET patient demonstrated a conserved CNV profile characterised by losses in Chr 3, 10, 11 & 13. All cells demonstrated loss of the VHL gene and a single cell also had unique gains in Chr 2 & 7 not seen in other cells, giving insight into clonal evolution in this patient. Potentially druggable targets including CDK6 and CDK4 were consistently present in CTC populations from single patients.

**Conclusion:** CNV analysis is feasible in CTCs derived from patients with NET and demonstrates both conserved and heterogeneous alterations. This type of analysis may allow personalised therapy and real time monitoring of tumour evolution.

Acknowledgement: A similar version of this abstract has been published previously, see https://www.karger.com/Article/Abstract/487699 for original and CC-BY license.

Disclosure: Funded by CCIC, ENETS

Corresponding author: Alexa Childs

### **70. Antibiotic use and efficacy of small molecule inhibitors in patients with advanced cancer**

#### Grace Tan^1^, Nadina Tinsley^2^, Cong Zhou^1^, Shaun Villa^2^, Paul Lorigan^2^, Fiona Blackhall^2^, Matthew Krebs^2^, Louise Carter^2^, Fiona Thistlethwaite^2^, Andrew Hughes^1^, Natalie Cook^2^

##### ^*1*^*University of Manchester*, ^*2*^*Christie NHS Foundation Trust*

**Background:** Antibiotic use (ABX) has been shown to reduce the efficacy of immune checkpoint inhibitors (CKIs) in the treatment of cancer by eradication of the gut microbiome. The gut microbiome is known to be involved in the regulation of anti-tumour responses. Here, we determine if the a similar relationship exists between ABX use and small molecule inhibitors (SMIs) in patients with advanced cancer.

**Method:** Retrospective data analysis was performed on metastatic cancer patients treated with SMIs at the Christie NHS Foundation Trust between January 2015 and March 2017. Patients with melanoma, renal cell carcinoma (RCC) and non-small cell lung cancer (NSCLC) patients were included. Patient demographics, prior treatments, extent of disease, SMI agent and use of antibiotics (route, duration, multiple ABX/successive courses) were collected. Progression free survival (PFS) and overall survival (OS) were compared between ABX+ (patients treated with ABX either 2 weeks before SMI initiation, or 6 weeks after) and ABX- groups (patients with no ABX within the specified timeframe). Statistical analyses were carried out with univariate and multivariate models.

**Results:** Of 261 patients, 69 (26%) received ABX of which the commonest were beta-lactams and quinolones. Multivariate analyses of ABX+ and ABX- groups found that no correlation exists between ABX use and PFS but ABX use was linked to shorter OS (median 490 days (ABX-) vs 309 days (ABX+)).

**Conclusion:** This is the first analysis investigating the impact of ABX on SMI therapeutic outcomes. The data demonstrated evidence of a link between ABX and shortened OS therefore further studies are warranted to fully evaluate the potential causative role of the gut microbiome in reduced treatment outcomes of SMIs as other forms of anti-cancer therapies.

Disclosure: None declared

Corresponding author: Grace Tan

### **71. MCL-1 regulates breast cancer stem cells**

#### Kirsteen Campbell^1^, Karen Blyth^2^, Stephen Tait^1^

##### ^*1*^*Glasgow University/Beatson Institute for Cancer Research*, ^*2*^*Beatson Institute for Cancer Research*

**Background:** MCL-1 is a pro-survival member of the BCL-2 protein family that has been shown to be up-regulated in a range of cancers. We have shown that high levels of MCL-1 predict poor prognosis in breast cancer. This is particularly relevant in triple negative breast cancer where treatment resistance and disease recurrence remain a major challenge. A new class of drugs specifically targeting MCL-1 have been developed and are currently in clinical trials for haematopoietic malignancies. We are investigating the therapeutic potential of targeting MCL-1 in breast cancer and the role of MCL-1 in breast cancer stem cells.

**Method:** We have used a combination of approaches to systematically evaluate the role of MCL-1 in breast cancer. This includes the use of small molecule MCL-1 inhibitors and knock-down/knock-out of MCL-1 in vitro and in vivo xenograft/genetically engineered mouse models to investigate the therapeutic potential of targeting MCL-1 in breast cancer.

**Results:** We find that in addition to the poor prognosis indicated by high levels of MCL-1, breast cancer cells are highly dependent on MCL-1. Therapeutic targeting or genetic deletion of MCL-1 is sufficient to delay tumour development and regress established tumours in vivo. We find that MCL-1 is important in breast cancer stem cells and targeting MCL-1 could be particularly important in the context of treatment resistance and recurrence.

**Conclusion:**  New targeted therapeutic approaches are required in both triple negative breast cancer and treatment resistant/recurrent disease. Our evidence suggests that targeting MCL-1 may offer a new therapeutic axis in breast cancer.

Disclosure: None declared

Corresponding author: Kirsteen Campbell

### **72. Amino acid transporter SLC7A5 is required for growth of Kras-mutant colorectal cancer in vivo**

#### Arafath Najumudeen, Fatih Ceteci, Owen Sansom

##### *Beatson Institute for Cancer Research*

**Background:** Colorectal Cancer (CRC) is the third most common cancer worldwide and Ras/MAPK pathway deregulation is strongly associated with the development of CRC, often through activating KRAS mutations (40%) and inactivation of APC (80%). Recent studies from our laboratory and others showed that expression of oncogenic Kras dramatically increases intestinal tumorigenesis initiated by the inactivation of *APC* in mice. However, the role of Kras mutation in metabolic rewiring of intestinal tumours is not yet fully understood.

**Method:** We use extensive combinations of genetically engineered mouse (GEM) models of intestinal cancer, targeted liquid chromatography/Mass spectrometry (LC/MS) metabolomics and transcriptomic analysis on intestinal organoids.

**Results:** Here we demonstrate in GEM models of intestinal cancer that Wnt activation (by Apc loss) and Kras mutation co-ordinately rewire intestinal metabolism in vivo and in vitro by increasing both glucose and glutamine consumption to support anabolic processes that fuel cell proliferation. From this analysis, we identified Solute carrier member 7a5 (Slc7a5) to be significantly upregulated in both mouse and human CRC to meet the increased nutrient demand of the cancer cells. This work aims to determine the mechanism by which Slc7a5 contributes to CRC. Using genetic deletion of Slc7a5 in murine models of CRC we show that Slc7a5 is functionally required for Kras driven (not wildtype) epithelial cell proliferation and tumorigenesis. Mechanistically, Slc7a5 is needed for Kras mediated nutrient sensing and subsequent mTOR1 signaling downstream. Importantly, Slc7a5 deletion sensitises Kras tumours to rapamycin (further suppressing mTORC1 signalling) causing tumour cells to undergo growth arrest.

**Conclusion:**  In conclusion, our results reveal a Kras mediated metabolic rewiring mechanism that couples amino acid transport by Slc7a5 and mTOR1 activation with control of intestinal tumorigenesis and further suggest that altering Slc7a5 activity may provide a therapeutic opportunity for colorectal cancer.

Disclosure: Funded by Beatson Institute for Cancer Research

Corresponding author: Arafath Najumudeen

### **73. The role of ELTD1/ADGRL4 in tumour angiogenesis**

#### Koon Hwee Ang

##### *University of Oxford*

**Background:**  ELTD1/ADGRL4, is an orphan adhesion GPCR. ELTD1 is expressed in endothelial and vascular smooth muscle cells but its expression in the tumour vasculature is significantly increased. Our aims were to analyse ELTD1’s function in endothelial cells and breast cancer to explore its potential as an anti-cancer therapeutic target.

**Method:** Primary human breast cancers (n = 245) and matched primary & nodal secondary breast cancers (n = 79) were stained for ELTD1. Staining intensity was scored and compared with survival. ELTD1 was expressed in breast cancer cell lines to assess the effect on tumour growth in xenograft experiments and knockout mice were generated with 1) tamoxifen-inducible knockout of Eltd1 in all tissues 2) constitutive Eltd1 knockout in vessels or 3) inducible Eltd1 knockout in vessels. Extracellular vesicles (EV’s) were harvested from ELTD1 expressing cells and their effects assessed *in vitro* and in vivo.

**Results:** Human breast cancer staining revealed a higher intensity vascular ELTD1 staining within the tumour stroma compared to normal stroma and ~15% of the tumours had ELTD1 expression within tumour cells. Higher ELTD1 expression in both the tumour stroma vasculature (n = 241; HR = 0.68; p = 0.04) and within the subset of tumour positive cases (n = 24; HR = 0.3; p = 0.02) correlated with improved relapse free survival (RFS). ELTD1 expression in human breast cancer cell lines did not affect proliferation or spheroid growth, but reduction in tumour growth was seen in xenograft models. Tumours grown in the constitutive and inducible Eltd1 knockout mice showed no difference in growth but marked changes in vasculature and necrosis. ELTD1 is incorporated into EV’s. Vesicles isolated from ELTD1 overexpressing breast cancer cell lines and HUVECs were pro-angiogenic in vitro and reduced tumour growth when injected into xenografts.

**Conclusion:** Eltd1 knockdown affects tumour vasculature, particularly in inducible models suggesting that monoclonal antibodies targeted the calcium binding EGF domain should be considered for further therapeutic development.

Disclosure: Funded by Cancer Research UK

Corresponding author: Koon Hwee Ang

## Early detection, diagnosis and prognosis

### **74. Neutrophil counts and cancer prognosis: an umbrella review of systematic reviews and meta-analyses of observational studies**

#### Meghan Cupp^1^, Margarita Cariolou^1^, Ioanna Tzoulaki^1^, Evangelou Evangelos^1^, Antonio Berlanga-Taylor^1^

##### ^1^*Imperial College London*

**Background:** Neutrophil counts have been linked to the progression of cancer due to their tumourigenic role in the cancer microenvironment. Numerous meta-analyses and individual studies explore the association between neutrophil counts and cancer prognosis, contributing to a large body of evidence with variable strength and validity. Uncertainty exists around the association between neutrophils and cancer outcomes depending on the site, outcome and treatments considered.

**Method:** For this umbrella review we searched Medline, EMBASE, and the Cochrane Database of Systematic Reviews for meta-analyses of observational studies evaluating the association between neutrophil to lymphocyte ratio (NLR) or tumour associated neutrophils (TAN) and specific cancer outcomes related to prognosis. The available evidence was graded as strong, highly suggestive, suggestive, or weak through the application of pre-set grading criteria. For each included meta-analysis, the grading criteria considered the significance of the random effects estimate, the significance of the largest included study, the number of studies and individuals included, the heterogeneity between included studies, the 95% prediction intervals, presence of small study effects, excess significance and credibility ceilings.

**Results:** Ultimately, 81 meta-analyses from 36 studies met the criteria for inclusion. All meta-analyses suggested a hazard ratio in the same direction of effect (HR>1). When assessed for significance and bias related to heterogeneity and small study effects, only three (4%) associations between NLR and overall survival and progression-free survival in gastrointestinal and nasopharyngeal cancers were supported by strong evidence.

**Conclusion:** Despite many publications exploring the association between NLR and cancer prognosis, the evidence is limited by significant heterogeneity and small study effects. There is a lack of evidence on the association between TAN and cancer prognosis, with all nine meta-analyses identified arising from the same study. Further research is required to provide strong evidence for associations between both TAN and NLR and poor cancer prognosis.

Disclosure: Funded by Medical Research Council

Corresponding author: Meghan Cupp

### **75. Salivary melatonin and squamous cell carcinoma antigen 1 levels in patients with oral squamous cell carcinoma**

#### Ivan Salarić^1^, Ivana Karmelić^2^, Jasna Lovrić^2^, Marko Rožman^3^, Davor Brajdić^1^, Ivan Zajc^1^, Igor Čvrljević^4^, Ksenija Baždarić^5^, Darko Macan^1^

##### ^*1*^*Department of Oral and Maxillofacial Surgery, University of Zagreb School of Dental Medicine, University Hospital Dubrava, Zagreb, Croatia*, ^*2*^*Department of Chemistry and Biochemistry, University of Zagreb School of Medicine, Zagreb, Croatia*, ^*3*^*Division of Physical Chemistry, Ruđer Bošković Institute, Zagreb, Croatia*, ^*4*^*Department of Oral and Maxillofacial Surgery, University Hospital Dubrava, Zagreb, Croatia*, ^*5*^*Department of Medical Informatics, Rijeka University School of Medicine, Zagreb, Croatia*

**Background:** Oral squamous cell carcinoma (OC) is one of the ten most frequent cancers in the world, with a 5-year survival rate between 30-60%. Melatonin (MLT) has anti-oxidant, anti-inflammatory and immunomodulating properties and is considered to contribute in protecting the oral cavity from tissue damage caused by endogenic and exogenic factors. Squamous cell carcinoma antigen (SCCA) has been recognized as a potential serum marker for various squamous cell carcinomas and other diseases. However, most of the studies on serum SCCA and OC have not distinguished SCCA1 and SCCA2, due to their similar chemical structure. It is important to mention that the two molecules play different roles in the organism. Aim of the proposed study is to determine salivary MLT and SCCA1 concentrations in patients with OC.

**Method:** Unstimulated whole saliva (UWS) and stimulated whole saliva (SWS) was sampled from 29 patients with OC (21 male, 8 female) and 29 age and sex matched healthy subjects (22 male, 7 female). Only patients with histologically diagnosed oral squamous cell carcinoma were included in the study. Sandwich human SCCA1 ELISA kits (My BioSource, San Diego, USA) and Melatonin direct saliva ELISA kits (IBL International GmbH, Hamburg, Germany) were used. This research has been funded by the Croatian Science Foundation (IP-09-2014-9376).

**Results:** Melatonin UWS levels were higher in the OC than in the control group (p=0.045; median OC: 2.18 pg/mL(95% CI:1.37-2.88), median control group: 0.84 pg/mL(95% CI: 0.50-1.71)). SCCA1 levels were higher in the OC group both in WUS (p=0.030; median OC: 244,26 pg/mL(95% CI:133,00 to 513,27), median control group: 2pg/mL(95% CI: 2,00-321,47)) and WSS (p=0.014; median OC: 478,03 pg/mL(95% CI:125,16-618,74), median control group: 2,00(95% CI:2,00-207,46)).

**Conclusion:** Salivary MLT and SCCA1 could serve as satisfactory biomarkers for OC. To our knowledge, MLT and SCCA1 have not yet been measured in the saliva of OC patients.

Disclosure: Funded by Croatian Science Foundation (IP-09-2014-9376)

Corresponding author: Ivan Salarić

### **76. Exploring experiences of referral for diagnostic investigations and attitudes towards the new Faster Diagnostic Standard**

#### Marianne Coleman^1^, Georgia Black^2^, Dorothee Amelung^1^, Emily Power^3^, Katriina Whitaker^1^

##### ^*1*^*University of Surrey*, ^*2*^*University College London*, ^*3*^*Cancer Research UK*

**Background:** The Faster Diagnosis Standard (introduced in England from 2020) is a new patient-centred policy stipulating that patients must have cancer ruled out or diagnosed within 28 days of referral for diagnostic testing. We explored public attitudes towards this new standard, within the context of their own recent referral experiences.

**Method:** Four 90-minute focus groups were conducted (2 Guildford, 2 Bradford, n=29), recruiting men and women aged 50+ years without a current cancer diagnosis, who had completed certain diagnostic tests (e.g. ultrasound) and received results within the last 6 months. Age, education and gender were evenly distributed across groups through purposive sampling. Topic guide was developed with input from people with cancer, Cancer Research UK, and the public.

**Results:** The biggest cause of concern to patients was the process of waiting for and obtaining test results. Most had experienced swift referral/testing, and it was difficult for participants to understand how the new standard could impact upon time spent progressing through the system. Responsibility for meeting the standard was also a concern: patients did not see their own behaviours such as accepting a cancellation appointment as a form of involvement in the standard being met. The GP’s role was conceptualised as communicating with the patient about their referral, establishing their preferences for information and continued involvement at each stage of the referral process. The standard legitimised chasing for test results, but 28 days was considered too long.

**Conclusion:** Patients should be asked what they would like to know about their referral. Where appropriate, GPs should be more transparent about the referral process and the potential for lack of clarity around next steps, timescales and outcomes. Patients should know that it’s ok to make use of opportunities perceived as ‘manipulating the system’ but this needs to be balanced against adding to existing patient burden.

Disclosure: Funded by Cancer Research UK

Corresponding author: Katriina Whitaker

### **77. Correlation between clinic-pathological features, MSI, PD-L1 and survival in resected gastric cancer: looking for prognostic biomarkers**

#### Margherita Ratti^1^, Nicola Valeri^2^, Jens Claus Hahne^2^, Andrea Lampis^2^, Michele Ghidini^1^, Gianluca Tomasello^1^, Giulia Tanzi^1^, Matteo Fassan^3^, Rodolfo Passalacqua^1^

##### ^*1*^*Cremona Hospital Institutes, Italy*, ^*2*^*Institute of Cancer Research*, ^*3*^*University of Padua, Italy*

**Background:** The identification of prognostic biomarkers for gastric cancer patient selection is compelling to improve survival outcomes. Microsatellite instability (MSI) is related with a positive prognostic effect in resected gastric cancer, whereas perioperative chemotherapy is detrimental. In metastatic MSI gastric cancer, immunotherapy with anti-PD1/PD-L1 drugs has shown promising results. Nevertheless, in early stages, data on the relation between MSI, clinic-pathological features, PD-L1 expression and overall survival (OS) remains sparse, especially in Western population. In this study, the prognostic role of MSI status, clinic-pathological features and PD-L1 expression in a large cohort of Italian gastric cancer patients was examined.

**Method:** Clinic-pathological data of 148 consecutive stage I-III gastric cancer patients resected in Cremona Institute between 2010 and 2014 (mostly chemo and/or radio-naive) were collected. MSI analysis was performed on tissue samples for all cases, by polymerase chain reaction. PD-L1 expression, evaluated by immunoistochemistry, was assessed in MSI patients. Differences between subgroups were evaluated with Chi-square test; Kaplan-Meier method and Long Rank test were used to calculate OS.

**Results:** Female sex (p=0.012), earlier TNM stages (p=0.011) and limited nodal involvement (p=0.029) significantly correlated with MSI status. MSI is significantly associated with prolonged survival (p<0.001), with an advantage of 28.6 months in OS compared with the microsatellite stable (MSS) group. Most MSI patients (71%) expressed PD-L1. Altough not statistically significant, MSI patients without PD-L1 expression showed a better trend in OS compared with MSI gastric cancer patients expressing PD-L1 and with MSS group.

**Conclusion:** MSI is an independent prognostic biomarker in gastric cancer and identifies a subset of patients with better OS and specific clinic-pathological features, including high expression of PD-L1. MSI could be a promising biomarker to select patients for chemotherapy versus immunotherapy in resectable gastric cancer.

Disclosure: Funded by Institute of Cancer Research

Corresponding author: Margherita Ratti

### **78. Focal Asymmetry and Laterality of Cancer**

#### Faraz Janan^1^, Michael Brady^2^

##### ^*1*^*University of Lincoln*, ^*2*^*University of Oxford*

**Background:** The BIRADS score for mammographic density does not currently indicate which breast has the higher density, and may be at higher risk of developing (or having) cancer. A focal density (‘developing’) is a region that is apparent on a recent mammogram, is present in both CC and MLO views, but can only be seen in one breast. Using focal density (FD) quantification, we assign a density score to each breast separately in order to assess breast asymmetry.

**Method:** Our method, which substantially improves our previous method (RICE) [1], suppresses normal breast parenchyma in order to highlight ROIs (see Figure 1). It embodies the reasonable assumption that the neighborhood typically has a similar tissue density to that of the tissue encompassing the candidate masked tumor. In this preliminary study, the method was applied to 11 patient cases of very dense mammograms, each of which contained at least one cancer. Our method produces a density score for each individual breast and determines asymmetry as a percentage score.

**Results:** Table 1 summaries our results. Except for a single instance (CL0081 – a marginal case), all asymmetric FD scores indicate the laterality of the cancer. All mammograms were pre-processed with Volpara breast density quantification software [2]. We note that in some cases (i.e. CL0037, CL0077, CL0090) where the volumetric density grade percentage VGD% is very similar for both the left and right breast, our method effectively produces distinct FD scores confirming to the laterality of cancer.

**Conclusion:** We assessed asymmetric FD in bilateral mammograms using a method based on the actual composition of the breast. In initial experiments, our method shows a promising potential for quantifying breast asymmetry with respect to the laterality of cancer. It works very well in all BIRADS classes, including BIRADS-D.

Disclosure: Funded by Matakin UK/ASSURE Project (2014-15)

Corresponding author: Faraz Janan

### **79. Improving the intra-operative diagnosis of high-grade glioma using a fluorescence biomarker – result of the UK NCRI GALA-BIDD study**

#### Colin Watts^1^, Keyoumars Ashkan^2^, Michael Jenkinson^3^, Kathreena Kurian^4^, Wendi Qian^5^, Stephen Price^5^, Tomasz Matys^6^, Gail Doughton^5^, Andrea Machin^5^, Josephine Jung^2^, Ibrahim Jalloh^3^, Chloe Harman^5^, Katrina Gatley^5^, Gemma Young^5^, Richard Hardy^5^, Alimu Dayimu^7^

##### ^*1*^*University of Birmingham*, ^*2*^*Kings College Hospital*, ^*3*^*University of Liverpool*, ^*4*^*Southmead Hospital*, ^*5*^*Cambridge University Hospitals NHS Foundation Trust*, ^*6*^*University of Cambridge*, ^*7*^*Shandong University*

**Background:** Correctly distinguishing gliomas as low or high grade (LGG or HGG) during surgery can influence the surgical procedure, enhancing resection and improving survival. The GALA-BIDD study was designed to prospectively investigate whether the presence of visible fluorescence is a pragmatic intra-operative diagnostic surgical biomarker of high grade disease within a tumour mass in real time during surgery.

**Method:** Patients with suspected intrinsic glioma discussed at neuro-oncology Multidisciplinary Team meetings and suitable for fluorescence guided cytoreductive surgery were eligible. 5-aminolevulinic acid (5-ALA) was used to generate visible fluorescence. Tissue samples were sent for peri-operative histopathological analysis to establish an intra-operative diagnosis of LGG or HGG. Presence of visible fluorescence was collected. These data were compared with the final central pathological diagnosis.

**Results:** From Feb 2015 to March 2017 in the UK, 106 patients were recruited: median age 59 (range 23–77); 59% male; 25% WHO radiological grade II transforming to a higher grade and 55% grade IV. 5-ALA were given for 103 patients with a median dose of 1500mg (range 960–2200mg). 67% of patients classified as HGG at local per-operative diagnosis were confirmed by the central review (weighted Kappa 0.37 (95%CI = 0.21–0.54)). 88 patients were evaluable for the primary endpoint: 81 had visible fluorescence of the tumour with central histopathology diagnosis as 1 LGG, 78 HGG (a 99% concordance in HGG classification with the 99%CI = 91%-99.9%) and 2 not assessed; 7 patients had no visible fluorescence and were diagnosed as 6 LGG and 1 HGG.

**Conclusion:** There is an urgent need to improve the local peri-operative diagnosis. The presence of visible fluorescence can be used as an additional pragmatic intra-operative diagnostic surgical biomarker of high-grade disease within a tumour mass. Use for assessment of low-grade disease needs further investigation.

Disclosure: Funded by Cancer Research UK

Corresponding author: Kathreena Kurian

### **80. Comparison of performance of mammogram readers with breast MRI readers at an abbreviated breast MRI interpretation task: Results from a single centre multireader study using an enhanced data-set**

#### Lyn Jones^1^, Sam Harding^1^, Rebecca Geach^1^, Chris Foy^2^, Victoria Taylor^1^, Andrea Marshall^3^, Janet Dunn*^3^

##### ^*1*^*North Bristol NHS Trust*, ^*2*^*Research Design Service, South West*, ^*3*^*Warwick Clinical Trials Unit, University of Warwick*

**Background:** FAST MRI has been proposed as a screening tool for a wider group of women than those currently offered screening with breast MRI. Its shorter acquisition and reading times promise potential cost effectiveness. However, any proposed change in screening modality from mammograms to FAST MRI has workforce implications. We need to know whether NHSBSP mammogram readers, already adept at mammogram interpretation but without previous experience of interpreting breast MRI, could quickly learn to read FAST MRI with brief additional training.

**Method:** 8 Readers (4 NHSBSP breast MRI and mammogram readers (Group 1) and 4 NHSBSP mammogram readers who do not read breast MRI (Group 2)) were trained using a standardized training package. All Readers completed a test set of 125 anonymised FAST MRI examinations (250 breasts: 194 normal and 56 cancer) blinded to the other Readers’ opinions; providing a total of 2000 interpretations.

**Results:** Overall concordance with the true result was 1745 (87%) when identifying MRI classification 4 or 5 as cancers (898/1000 (90%) for Group 1 Readers; 847/1000 (85%) Group 2 Readers). The agreement between all readers and the true value, kappa, was 0.69 (95% ci 0.65–0.72).

When identifying MRI 3, 4 or 5 as cancers, overall concordance was 1550 (78%) for all readers (777 (78%) Group 1; 773 (77%) Group 2) and kappa = 0.51 (95% ci 0.47-0.54).

**Conclusion:** These results suggest that NHSBSP image readers could adapt to reading FAST MRI with brief additional training.

Ethics: Integrated Research Application System (IRAS), Health Research Authority (HRA) and Research Ethics Committee (REC) approval obtained (IRAS: 219332, REC: 17/SW/0142, EDGE: 4002). – https://www.hra.nhs.uk/planning-and-improving-research/application-summaries/research-summaries/

Disclosure: Funded by North Bristol Hospital Trust Research Capability Funding

Corresponding author: Lyn Jones

### **81. Patterns in haemoglobin levels over 10 years to predict diagnosis of colorectal cancer by Duke’s staging: preliminary findings using UK primary care routine blood test data**

#### Pradeep S. Virdee^1^, Tim Holt^2^, Julietta Patnick^3^, Jacqueline Birks^1^

##### ^*1*^*Centre for Statistics in Medicine, University of Oxford*, ^*2*^*Nuffield Department of Primary Care Health Sciences, University of Oxford*, ^*3*^*Nuffield Department of Population Health, University of Oxford*

**Background:** Stage at diagnosis of colorectal cancer influences 5-year survival: 94% at the earliest stage, but 7% at the latest. Tumour growth causes subtle changes in levels of blood components, such as haemoglobin, which may go unnoticed. Such changes have not been explored. We report patterns in haemoglobin levels up to 10 years before a diagnosis of colorectal cancer, by Duke’s tumour staging.

**Method:** This retropspective study used data from the Clinical Practice Research Datalink linked to the UK Cancer Registry. Patients with a colorectal cancer diagnosis or no diagnosis of any cancer, and at least one blood test result were included. The yearly rate of change in haemoglobin levels was calculated from longitudinal patient blood test data.

**Results:** 798 of the 68,301 patients included had a diagnosis of colorectal cancer: 76 at stage A, 180 at B, 193 at C, 57 at D, and 292 with missing staging. Median age at diagnosis was 73.1 years and 52.8% were males. On average, the rate of decline in haemaglobin levels was higher from 3 years before diagnosis compared to earlier, but remained constant among those not diagnosed. At stage A, average haemoglobin levels declined at a rate of 1.10g/dL from 1 year before diagnosis, but by 1.31g/dL at stage D, observed from 2 to 1 year before diagnosis. A decline in average haemoglobin levels commenced 1 year before diagnosis of stage A tumours, but at 3 years of stage D tumours.

Results from age- and gender-stratified analyses will be presented.

**Conclusion:** Compared to those without cancer, those with colorectal cancer had an increased rate of decline, detected earlier in those with Dukes D. Subtle decreases in haemoglobin levels over time may identify colorectal cancer in the early stages. Patterns in other blood components, such as platelets, will be explored.

Disclosure: Funded by NIHR Oxford Biomedical Research Centre

Corresponding author: Pradeep S. Virdee

### **82. Radiology review of MRI screening used to detect rSCC (radiological spinal cord/compression) in a randomised trial of asymptomatic castrate resistant prostate cancer patients with spinal metastasis**

#### Aslam Sohaib^1^, Keeley Tomkinson^2^, Philip Rich^3^, Graeme Houston^4^, Clare Cruickshank^2^, Clare Griffin^2^, Shama Hassan^2^, Laura Wiley^2^, Stephanie Gibbs^5^, Ann Henry^6^, Gail Horan^7^, Heather Payne^8^, Ian Pedley^9^, Narayanan Nair Srihari^10^, Emma Hall^2^, David Dearnaley^1^

##### ^*1*^*Royal Marsden Hospital*, ^*2*^*Institute of Cancer Research*, ^*3*^*St Georges Hospital London*, ^*4*^*University of Dundee*, ^*5*^*Queen's Hospital Romford*, ^*6*^*Leeds Teaching Hospital*, ^*7*^*Addenbrooke's Hospital*, ^*8*^*University College Hospital*, ^*9*^*Newcastle Hospital*, ^*10*^*Royal Shrewsbury Hospital*

**Background:** PROMPTS (CRUK/11/053) is a phase III randomised controlled trial which investigates whether detection of radiological spinal cord/canal compression (rSCC) by screening MRI of the spine reduces the incidence of clinical spinal cord/canal compromise or compression (cSCC) in asymptomatic castrate resistant prostate cancer (CRPC) patients with spinal metastasis.

**Method:** 420 CRPC patients from 53 UK centres, recruited between 21/01/2013 and 28/04/2017, were randomised to a screening MRI (n = 210, experimental) or standard follow-up (n = 210, control). All MRI scans were reported by a named radiologist at the local centre and a sample were reviewed centrally by a panel of radiology specialists to ensure consistency and accuracy of rSCC detection. The modified Bilsky Spinal Cord Compression Scale was used to assign the degree to which SCC had occurred in each vertebra; a 7-point scale ranging from 0 ‘metastatic bone disease without epidural impingement’ to 3 ‘spinal cord compression, no CSF visible around the cord’, 9 was assigned if no metastasis were present. Patients with screen-detected rSCC (locally assessed Bilsky score 1a,1b,1c,2,3) were offered pre-emptive treatment and 6-monthly follow-up MRI scans. Local and central assessments of the screening scans were compared at the vertebra level with a discrepancy of +/− 1 Bilsky score and +/− 1 vertebral level deemed acceptable (pragmatic agreement).

**Results:** 2060 vertebrae were locally and centrally assessed; the reviewed scans included both positive (rSCC identified) and negative (no rSCC identified) scans. On central review, the median number of vertebra with metastatses per spine was 1 (range 0-10) and the most common classification of rSCC was Bilsky grade 1a. Pragmatic agreement was 98.8%. Complete agreement was 92.4%.

**Conclusion:** The high level of agreement seen suggests that the Bilsky scoring system is robust and reproducible in assessing the presence and degree of rSCC in patients with CRPC.

Disclosure: Funded by Cancer Research UK

Corresponding author: Aslam Sohaib

### **83. Metabolic Pathways and Cell Proliferation in the Advancing Front of Oropharyngeal Squamous Cell Carcinoma**

#### Khaled Ben Salah^1^, Asterios Triantafyllou^2^, Andrew Schache^1^, Richard Shaw^1^, Janet Risk^1^

##### ^*1*^*Department of Molecular & Clinical Cancer Medicine, University of Liverpool, UK*, ^*2*^*Department pf Pathology, Liverpool Clinical Laboratories and School of Dentistry, University of Liverpool, Liverpool, UK*

**Background:** Events related to progression of tumours are centred on their advancing front. Metabolism likely influences cell proliferation and a three compartment metabolic model in head and neck cancer has been proposed. Little attention, however, has been paid to the relationship between metabolism and proliferation in oropharyngeal squamous cell carcinoma (OPSCC). We investigated these properties at the advancing front of OPSCC in relation to HPV status.

**Method:** Tissue microarrays were constructed from 45 HPV(+) and 63 HPV(-) OPSCCs and examined by IHC for mitochondrial (TOMM20), metabolic pathways (monocarboxylate transporter 1/4; MCT1/4) and cell proliferation (Ki67) markers. Immunoreactivity was recorded at the tumour advancing front and in adjacent stroma.

**Results:** Cytoplasmic, granular TOMM20 and nuclear Ki67 were highly expressed at the advancing front of OPSCCs, independent of HPV status. Co-expression of membranous MCT1 and MCT4 at the advancing front was observed in 84% and 30% of the HPV(+) and HPV(-) tumours, respectively (p=0.001). While all HPV(+) tumours showed MCT4(+) stromal spindled cells, regarded as myofibroblasts or cancer-associated fibroblasts, adjacent to the advancing front, only 70% of the HPV(-) tumours did so (p=0.002).

**Conclusion:** Tumour cells at the advancing front of both HPV(+) and HPV(-) OPSCC are rich in mitochondria, show similar proliferative activity, and consume mitochondrial fuel (lactate), imported via MCT1 expression. The different frequencies of co-expression of MCT1 and MCT4 in HPV(+) and HPV(-) OPSCC indicate that the three compartment model is more compatible with data from HPV(-) OPSCC. The metabolism of the stromal component differs between HPV(+) and HPV(-) tumours, but these differences are not reflected in the proliferative activity of the tumour cells in the advancing front. We conclude that tumour metabolism in OPSCC may be related to HPV status, but is complex and requires further examination.

Disclosure: Funded by University of Liverpool

Corresponding author: Khaled Ben Salah

### **84. Attitudes towards faecal immunochemical testing in patients at increased risk of colorectal cancer in primary care: a survey of English General Practitioners**

#### Christian von Wagner^1^, Sandro Stoffel^1^, Helga Laszlo^1^, Brian Nicholson^2^, Jessica Sheringham^1^, Dorothy Szinay^1^, Yasemin Hirst^1^

##### ^*1*^*University College London*, ^*2*^*Nuffield Department of Primary Care*

**Background:** There is increasing interest in using quantitative Faecal Immunochemical Test (FIT) to rule-out colorectal cancer (CRC) among patients with high-risk symptoms in primary care. The study aimed to investigate general practitioners’ (GP) attitudes and willingness to use FIT over urgent two-week wait (2WW) referral.

**Method:** As part of the UCLH Cancer Collaborative ('Q-Fit pilot research study'), we conducted a cross-sectional online survey with 1024GPs based in England. Data were analyzed using Logistic regression models were used to explore their likelihood of using FIT instead of 2WW, and reported using odds ratios and confidence intervals.

**Results:** Just over a third of GPs (n=365) preferred to use FIT as a rule-out test over 2WW. GPs were more willing if they were aged 36-45 (1.59 [1.04-2.43]) and 46-55 (1.99 [1.14-3.47]), thought FIT was highly accurate (1.63 [1.16-2.29]), thought patients will benefit compared to a colonoscopy (2.02 [1.46-2.79]) and were highly confident in discussing the benefits of FIT (2.14 [1.46-3.16]). GPs were less willing if they had had more than 10 urgent referrals in the last year (0.62 [0.40 - 0.94]) and thought that longer consultations will be needed (0.61 [0.44 - 0.83]).

**Conclusion:** Our findings suggest that the acceptability of FIT as a rule-out test in primary care is currently low with less than half of GPs who perceived FIT to be accurate preferring it over colonoscopy. Any potential guideline changes recommending FIT in high-risk patients instead of urgent referral to rule-out CRC are likely to require intensive supporting educational outreach to increase GP confidence in the accuracy and application of FIT in this context.

Disclosure: Funded by NHS Cancer Vanguard Programme and delivered by UCLH Cancer Collaborative

Corresponding author: Christian von Wagner

### **85. Characterising the metabolic fate of (2S, 4R)-4[19F]-fluoroglutamine in cancer cells using 19F-MRS**

#### Nhat Nguyen, Andreas Doepner, Graham Smith, Yuen-Li Chung

##### *The Institute of Cancer Research*

**Background:** The (2S, 4R)-4-[^18^F]-fluoroglutamine (“[^18^F]-fluoroglutamine”) Positron Emission Tomography (PET) radiotracer is potentially useful for imaging glutamine metabolism in cancer cells. A first in man clinical trial of [^18^F]-Fluoroglutamine has been completed in adult glioma patients and imaging was shown to differentiate between stable and progressive disease. Despite these promising results, further metabolic characterisation of this probe is required to fully understand the metabolic fate of [^18^F]-fluoroglutamine in cancer cells. The aim of this study is to evaluate the metabolic pathways of the [^18^F]-Fluoroglutamine PET tracer by using [^19^F]-Magnetic Resonance Spectroscopy (MRS).

**Method:** The metabolic pathways of the [^18^F]-Fluoroglutamine PET tracer were evaluated by using the stable isotope version of this radiotracer in [^19^F]-MRS. [^1^H]-MRS study was also performed as a comparison method to confirm the consistency of the metabolism of [^19^F]-fluoroglutamine with normal L-glutamine. Colorectal cancer models (HCT116 WT and HCT116 Bax-ko) were treated with glutaminase inhibitor CB-839 and alanine aminotransferase inhibitor L-cycloserine in order to determine the possible involvement of these enzymes in the metabolism of [^19^F]-fluoroglutamine.

**Results:** Following treatment with glutaminase inhibitor CB-839, a significant increase in cellular [^19^F]-fluoroglutamine was found in the treated group when compared with control (p<0.01). A corresponding decrease in cellular [^19^F]-fluoroglutamate was observed following the CB-839 treatment (p<0.01). Following treatment with alanine aminotransferase inhibitor L-cycloserine, the cellular [^19^F]-fluoroglutamate increased significantly (p<0.001). A drop in free fluoride secretion was seen following L-cycloserine treatment (p<0.001). Similar changes in L-glutamine metabolism were also observed by 1H-MRS following treatment with these two inhibitors.

**Conclusion:** This study found that similar to L-glutamine, [^19^F]-fluoroglutamine can be hydrolysed by the glutaminase enzyme to yield [^19^F]-fluoroglutamate which can subsequently be metabolised by the alanine aminotransferase enzyme to form α-ketoglutarate, L-alanine and release free fluoride. Therefore, this study infers that the metabolism of the [^18^F]-Fluoroglutamine PET tracer follows the same pathway as L-glutamine in cancer cells.

Disclosure: Funded by Cancer Research UK and The Institute of Cancer Research

Corresponding author: Nhat Nguyen

### **86. A novel blood-based biomarker for OAC with the potential for use as an early treatment response predictor**

#### Rachel Lawrence^1^, Lucy Swithenbank^2^, Hasan Haboubi^2^, Lisa Williams^3^, Sarah Gwynne^3^, Shareen Doak^4^, Gareth Jenkins^2^

##### ^*1*^*Swansea University Medical school*, ^*2*^*Swansea University medical school*, ^*3*^*ABMU NHS Trust*, ^*4*^*Swnasea University medical school*

**Background:** Oesophageal adenocarcinoma (OAC) is the 13th most common cancer in the UK with 9 211 new cases diagnosed annually. Due to the late stage in which OAC is commonly diagnosed, treatment options are usually limited to chemo/radiotherapy with limited success, meaning the 5-year survival rate remains poor at 15%.

Here we describe a novel blood-based biomarker for OAC based on the PIG-A mutation assay, which has the further potential of predicting early treatment response. We hypothesise that cancer patients will have a higher level of this circulating mutation compared to controls. Furthermore, will patients undergoing chemo/radio therapy who display an increase in mutation frequency during their treatment respond better to such therapies?

**Method:** Blood samples were obtained from consenting patients attending endoscopy including patients with reflux disease, Barrett’s metaplasia (BM) and newly diagnosed OAC. Multiple blood samples were obtained from patients with OAC throughout their treatment course. Ten microliters of whole blood was stained with antibodies against the erythrocyte marker CD235a and two GPI-linked proteins (CD55 and CD59) and analysed using flow cytometry.

**Results:** With over 300 participants currently recruited to the study including healthy volunteers, data shows that treatment naïve OAC patients had a 3-fold increase in PIG-A mutant frequency compared to reflux patients (p<0.001). Reflux (n=75), Barrett’s (n=63) and treatment naïve cancer patients (n=38) had a median PIG-A mutant frequency of 3.2 (95% CI: 1.51-5.43), 4.52 (95% CI: 2.53-6.09) and 9.75 (95% CI: 3.76-17.52) respectively.

OAC patients undergoing treatment (n=13) had intra-individual variation of 1 to 8 fold increase in mutation frequency compared to their pre-treatment levels, with mutant frequencies in the range of 1.0-29.8 mutant RBC’s/million. Correlation with therapeutic response is on-going.

**Conclusion:** The PIG-A mutation assay has potential as a less-invasive biomarker for OAC as well as a treatment-response tool to identify patients with limited response to current therapies.

Disclosure: Funded by Cancer Research Wales and Health and Care Research Wales

Corresponding author: Rachel Lawrence

### **87. Image fusion targeted prostate biopsy in 771 men at risk: a multi-centre evaluation showing low diagnostic yield of significant cancer in non-targeted biopsies**

#### Saiful Miah^1^, Feargus Hoskings-Jervis^2^, David Eldred-Evans^2^, Taimur Shah^2^, Mark Laniado^3^, Richard Hindley^3^, Alan Doherty^3^, Andrew Sinclair^3^, Daniel Burke^3^, Jeetesh Bhardwa^3^, Omer Karim^3^, Bruce Montgomery^3^, Simon Bott^3^, Neil Barber^3^, Raj Nigam^3^, Manit Arya^4^, Mathias Winkler^1^, Clare Allen^4^, Hashim Ahmed^2^

##### ^*1*^*Imperial College NHS Healthcare*, ^*2*^*Imperial College London*, ^*3*^*Nuada Urology London*, ^*4*^*University College London Hospital*

**Background:** MRI image guided prostate biopsy is an increasingly utilized method of procuring tissue from men with suspected prostate cancer. We sought to report the largest prospective series of image-fusion transperineal prostate biopsies and compare the diagnostic yield of clinically-significant prostate cancer (csPCa) between targeted and non-targeted biopsies.

**Method:** 771 men had transperineal image-fusion targeted biopsy (MIM-Symphony-DX^TM^) (April 2014-June 2017) by 13 urologists.

**Results:** Mean age, median PSA and median prostate volume were 63.58 years, of 6.3ng/ml and 43.5cc respectively. Overall, 363 (47.08%) were diagnosed with csPCa (Gleason >/=4+3 or any grade >/=6mm) from the 771 men undergoing biopsy. The csPCa detection rate from the target biopsies alone occurred in 341 men (44.23%). Of those 409 men who underwent random biopsies, only 14 men (3.42%) were discovered to have csPCa exclusively in the non-targeted cores alone where the targeted cores demonstrated no evidence of disease. None of these men harboured high-grade disease and 11 out of the 14 (78.57%) had primary pattern 3 disease. The presence of clinically insignificant disease in the random targets occurred in 66 of the 409 (16.12%) men underdoing going this type of biopsy format. Insignificant cancer exclusively occurred in non-targeted prostate cores in 16 out of 409 men who underwent these random biopsies (3.91%) where there was no evidence of disease in the target cores.

**Conclusion:** In this large multi-centre series, the added benefit of non-targeted cores is low. An image-fusion targeted-biopsy only has high detection for csPCa and reduced over-detection of insignificant cancers.

Acknowledgement: A similar version of this abstract has been published previously, see http://journals.sagepub.com/doi/10.1177/2051415818773021 for original and CC-BY license.

Disclosure: None declared

Corresponding author: Saiful Miah

### **88. Investigation of transglutaminase 2 as a biomarker of invasive breast cancer that is likely to metastasise**

#### Fiona Blows^1^, Elena Provenzano^2^, Raza Ali^3^, Wei Cope^4^, Paul Pharoah^4^, Claire Pike^5^, Peter Coussons^5^

##### ^*1*^*Anglia Ruskin University and University of Cambridge*, ^*2*^*Addenbrooke's Hospital*, ^*3*^*University of Cambridge and University of Zurich*, ^*4*^*University of Cambridge*, ^*5*^*Anglia Ruskin University*

**Background:** Breast cancer, a heterogeneous disease, may be subtyped using immunohistochemistry (e.g. estrogen receptor (ER) and human epidermal growth factor receptor 2 expression). Differences in survival occur between apparently similar cases. Thus, better resolution of the disease into clinically meaningful subtypes is needed. Breast cancer lacks a biomarker for invasive disease that will metastasise and the discovery of such a biomarker would represent a significant step forward. Transglutaminase 2 (TG2), a multifunctional protein implicated in cell-cell adhesion, apoptosis and metastatsis, warrants investigation. Levels have been reported to vary with tumour progression.

**Method:** We used tissue-micro-arrays (TMAs) to evaluate TG2 expression in the tumours of 1,942 patients for whom we have associated data for survival time (72% alive; 28% dead), and tumour grade and stage. 1,917 samples also have hormone status data (79%, positive; 21%, negative). Hormone status was derived from ER and progesterone receptor (PR) expression data (designated ‘positive’ if either was positive, ‘negative’ if both were negative and where only the status of ER or PR was available this was assigned individually as the hormone status). TMAs were scored manually, and tumours expressing TG2 in more than 10 per cent of cells were categorised as ‘TG2 positive’.

**Results:** AlthoughTG2 was expressed in 37% of tumours it was not associated with outcome in hormone receptor positive disease. This was not the case in hormone receptor negative disease where its expression was associated with decreased breast cancer specific mortality (HR, 0.57; 95% CI, 0.37 − 0.89; p value, 0.012).

**Conclusion:** These results need confirming in a larger study. Nevertheless, the molecular basis of this finding if true would be intriguing, given the established links between TG2 and tumourigenesis. TG2 might be a novel drug target. Furthermore, given the prognostic effect-size, TG2 could prove a useful marker for identifying patients most likely to benefit from adjuvant chemotherapy.

Disclosure: Funded by Cancer Research UK; Horizon 20/20

Corresponding author: Fiona Blows

### **89. Cholangiocarcinoma survival prediction using machine learning algorithms**

#### Mohamed Osman^1^

##### ^*1*^*Faculty of Medicine, Zagazig University, Egypt*

**Background:** Cholangiocarcinoma is the most common malignancy of biliary tract and the second most common primary hepatic malignancy. The incidence and mortality of cholangiocarcinoma are increasing worldwide. Predicting cholangiocarcinoma survival is difficult due to different primary sites, treatments and undefined risk factors. Reliable predictions can help in personalizing care and good treatment and management. Here, we test the ability of machine learning to predict survival of cholangiocarcinoma.

**Method:** Patients with cholangiocarcinoma were identified through the Surveillance, Epidemiology and End Results database from 2010-2013. Patients’ data were extracted including: age, sex, race, primary site, TNM stage, grade, size, extension, lymph nodes, metastasis, cancer sequence number, surgery, radiotherapy, chemotherapy, radiation sequence with surgery, state, county and survival months. Data were randomly divided into; a training set (80%) and a validation set (20%). Machine learning algorithms were used to predict survival.

**Results:** A total number of 1,095 patients were identified with a median survival of 15 months. The most common primary tumor sites were intrahepatic bile duct (52.6%), extrahepatic bile duct (27.7%), and liver (16.3%). Random Forests algorithm achieved better results compared to other tested models. For evaluating model performance, the Area Under the Receiver Operating Characteristic Curve (AUC) was calculated. Random Forests yielded AUCs of 81.5% at 6 months, 87.1% at 12 months and 80.6% at 24 months. Sensitivity of the trained model were 83%, 80% and 76% at 6, 12 and 24 months, respectively. The model achieved an average accuracy of 82.6%, 80.4% and 75.8% at 6, 12 and 24 months, respectively. The most important characteristics which influenced the model performance were: surgery, age, and tumor size.

**Conclusion:** Supervised machine learning algorithms achieved a good performance of predicting survival of patients with cholangiocarcinoma. Improved performance in survival prediction can help in making better treatment decisions and planning social and care needs.

Disclosure: None declared

Corresponding author: Mohamed Osman

### **90. Investigation of exosomal miRNA as biomarker for prostate cancer castration resistance development**

#### Tianyu Guo^1^, Yang Wang^1^, Xueying Mao^1^, Lei Xu^1^, Jacek Marzec^1^, Edwina Burke^1^, Greg Shaw^2^, John Hines^2^, Prabhakar Rajan^1^, Karen Tipples^3^, Daniel Berny^1^, Jonathan Shamash^3^, Yong-jie Lu^1^

##### ^*1*^*Barts Cancer Institute, Queen Mary University of London*, ^*2*^*Department of Urology, Barts Health NHS, London, UK*, ^*3*^*Department of Medical Oncology, Barts Health NHS, London, UK*

**Background:** Prostate cancer (PCa) remains the most frequently diagnosed cancer and the third-leading cause of oncological mortality in western men. Androgen deprivation therapy (ADT) has been the standard care for initial management of locally advanced or metastatic PCa, however progression to castration-resistant prostate cancer (CRPC) would inevitably occur. Studies have shown that miRNAs play a role in CRPC and miRNAs in plasma exosomes may serve as biomarkers in cancers.

**Method:** Whole miRNA sequencing was performed to identify candidate exosomal miRNAs in a screening cohort of 24 treatment-naive PCa patients and 24 CPRC patients. Quantitative real-time polymerase chain reaction (qRT-PCR) was performed to further validate candidate miRNAs in a set of 108 treatment-naive PCa patients and 42 CRPC patients. An additional group of 43 non-CRPC patients under hormone therapy was also evaluated.

**Results:** In the screening cohort, miRNA sequencing generated an average of approximately 5-million reads per sample and identified differentially expressed miRNA. Six miRNAs were selected for further validation by qRT-PCR, which confirmed four out of the six candidate miRNAs comparing treatment-naïve PCa and CRPC plasma exosomes (*p*-value<0.05), while the expression of one miRNA was too low to be detected by our qRT-PCR methods. QRT-PCR results also showed that the expression of these four miRNAs were differently expression in non-CRPC treatment patients compared to CRPC patients (*p*-value<0.0001). When a receiver operating characteristic curve (ROC) was applied to distinguish CRPC from treated non-CRPC treatment patients, the area under the ROC curve (AUC) of the four miRNAs were 0.895(95% CI: 0.824–0.966), 0.807(95% CI: 0.712–0.902), 0.818(95% CI: 0.721–0.914) and 0.776(95% CI: 0.675–0.876), respectively. When combining the four miRNAs using a binary logistic regression model, the AUC achieved 0.945(95%CI: 0.893–0.998).

**Conclusion:** This study demonstrated that plasma exosomal miRNAs may serve as biomarkers for prostate cancer castration resistance development and clinically for CRPC occurrence prediction.

Disclosure: Orchid, Chinese Scholarship Council

Corresponding author: Tianyu Guo

### **91. Role of staging bone scans in the patients with newly diagnosed prostate cancer. Are the current guidelines acceptable?**

#### Mohanarangam Thangavelu^1^, Ahmed Kotb^2^, Chrysovalantis Gkekas^2^, Mohamed Yehia^3^, Jason Walker^1^

##### ^*1*^*Ysbyty Gwynedd*, ^*2*^*Bangor Hospital*, ^*3*^*Wrexham Hospital*,

**Background:** Most guidelines recommend bone scan to newly diagnosed patients with prostate cancer presenting with high risk criteria. The aim of our study is to investigate whether these recommendations are practically acceptable without missing significant bone metastasis in various risk groups of prostate cancer patients.

**Method:** Retrospective data collection, for all patients who had PSA, physical examination of prostate(DRE), prostate biopsy(Gleasons Group) and bone scan done, as a part of investigations to patients with newly diagnosed prostate cancer. Data was collected from a single Hospital for a period of 6 years.

**Results:** Total - 669 patients. 520 / 669 patients had all three criteria PSA, DRE and Histology. 149 / 669 patients were excluded because Histology was not available. 94 / 520 (18 %) had Positive and 426 / 520 (82%) had Negative bone scan.. The mean age is 71 ± 7.8 years. The range of PSA in the study is 4439 ng/ml. The NPV of bone scan in patients with PSA ≤ 20,Gleason group ≤ 2 and DRE (T1/T2) were 91.7%,90.8% and 88.2 % respectively. The PPV of bone scan in patients with PSA > 20, GG ≥ 3 and DRE (T3/T4) were 29.2%,25.8% and 36.9 % respectively.

In PSA ≤ 20, 23 patients had positive BS. Out of 23 patients, 5 patients had Gleason Group ≤ 7 with DRE T1/T2. So 5/94 (5.3 %) patients with bone metastasis could have been missed in the absence of high risk criteria mentioned in the guidelines.

**Conclusion:** The current guidelines are fairly accurate. However according to our study there is 5.3 % chance of having bone metastasis in the newly diagnosed prostate cancer patients, who lack significant risk factors for bone metastasis.This should be discussed with the patients.Large multi-centre studies are required to review the risk criteria for bone metastasis in prostate cancer.

Disclosure: None declared

Corresponding author: Mohanarangam Thangavelu

### **92. Pre-diagnostic BMI and ovarian cancer survival in the Million Women Study**

#### Kezia Gaitskell^1^, Carol Hermon^1^, Sarah Floud^1^, Kirstin Pirie^1^, Isobel Barnes^1^, Jane Green^1^, Gillian K Reeves^1^, Valerie Beral^1^

##### ^*1*^*University of Oxford*

**Background:** It is known that higher body mass index (BMI) is associated with increased risk of ovarian cancer, with some heterogeneity by tumour histotype. Ovarian cancer survival may also vary with pre-diagnostic BMI, but evidence is mixed, and studies recording BMI retrospectively may not have accounted fully for changes in weight preceding cancer diagnosis. We explored the association between prospectively-recorded BMI and ovarian cancer survival in a cohort of UK women.

**Method:** Study participants completed a questionnaire on reproductive, anthropometric, and lifestyle factors, including height and weight, at recruitment in 1996-2001, and were followed for cancer and death via national registries. We used Cox regression models to estimate adjusted relative risks (RRs) of death attributed to ovarian cancer associated with BMI, overall and by histotype.

**Results:** Among 9324 women diagnosed with ovarian cancer, 5352 had died with cause of death attributed to ovarian cancer after 4.4 years (SD 4.6) mean follow-up, with 43% 5-year survival. Average time between recording of BMI and diagnosis of ovarian cancer was 8.77 years (SD 4.93). Deaths were rare for serous and mucinous borderline tumours. Further analysis was restricted to the 4333 women with malignant ovarian cancer who had information on stage at diagnosis. After adjustment for age at diagnosis, stage, and tumour histotype, obese women (BMI 30+ kg/m^2^) had worse survival than women with normal BMI (<25 kg/m^2^) (HR = 1.11, 95% CI: 1.00-1.24), but adjustment for reproductive, anthropometric and lifestyle characteristics weakened the association (HR = 1.08, 0.97-1.21, p = 0.18). There was no significant variation by tumour histological type: serous carcinoma RR = 1.04, 0.90-1.21; mucinous carcinoma RR = 1.56, 0.86-2.84; endometrioid RR = 1.10, 0.66-1.81; clear cell RR = 1.41, 0.81-2.45; other/ unspecified RR = 1.07, 0.88-1.29 (heterogeneity: p = 0.2).

**Conclusion:** After adjustment for stage and other confounders, there was no significant association between pre-diagnostic BMI and ovarian cancer survival, and this did not vary significantly by histotype.

Disclosure: Funded by Cancer Research UK, the Medical Research Council, and the National Institute for Health Research.

Corresponding author: Kezia Gaitskell

## Prevention

### **93. Communicating messages about alcohol-related BC risk: the Alcohol and Breast Cancer (ABC) study**

#### Emma Miller^1^, Paul Ward^1^, Ian Olver^2^, Samantha Meyer^3^, Darlene McNaughton^1^, Lillian Mwanri^1^, Kristen Foley^1^, Sara Macdonald^4^

##### ^*1*^*Flinders University*, ^*2*^*University of South Australia*, ^*3*^*University of Waterloo*, ^*4*^*University of Glasgow*

**Background:** Breast cancer (BC) incidence in ‘middle-aged’ women (45-64 years) is increasing in industrialised countries, despite ongoing warnings about modifiable risk factors such as alcohol, the consumption levels of which have also increased in middle-aged women. Alcohol use, entrenched in Australian society, is directly linked to BC yet is seldom proposed as a causal mechanism by women at risk.

We investigated the place of alcohol in the lives of South Australian women at various levels of perceived risk for BC – exploring the ways in which women think about BC in the context of alcohol consumption as a socially-entrenched practice.

**Method:** In 2017, 122 women completed a brief online purposive sampling survey. After cross-characterising responses according to perceived cancer risk, level of alcohol consumption and education level, 35 women were interviewed. The audio-recorded interviews were transcribed, coded and thematically analysed.

**Results:** Socialising, relaxing and coping were the main purposes of alcohol consumption in this group. Women with higher socio-economic status (SES) tended to talk about alcohol as a pleasurable social lubricant, while lower SES women tended to talk about alcohol as a mechanism to cope and manage stress. The principal marker of problematic alcohol use overall was ‘functionality’ in work, care-giving and social roles.

BC candidacy was determined by family history with BC development generally seen as unpreventable and framed as ‘bad luck. Where a role for alcohol in BC was accepted, the risk was weighed against the important social function of alcohol.

**Conclusion:** In this group, most aspects of alcohol use were framed as social phenomena firmly outside of which context was the concept of alcohol-related BC risk. Prevention activities will need to respond to the social functions of alcohol, and appropriately target their SES differences, if they are to enact meaningful behaviour change to reduce BC risk.

Disclosure: Funded by Flinders Foundation

Corresponding author: Emma Miller

### **94. European Cancer Incidence is Significantly Reduced in Huntington’s Disease Patients – Unravelling it’s Protective Mechanisms**

#### Paul McNulty^1^, Raviram Ramesh^1^, Richard Pilcher^1^, Lesley Jones^1^, Alys Hughes^1^, Daniel Farewell^1^, Timothy Stone^1^, Peter Holmes^1^, Renata Neciunaite^1^

##### ^1^Cardiff University

**Background:** Huntington's disease (HD) is a neurodegenerative genetic disorder caused by the trinucleotide expansions of CAG that results in the formation of elongated polyQtracts. It is hypothesised that these polyQtracts accumulate intracellularly causing cellular apoptosis prior to tumour-induced cell division.^1^ Thus, expanded polyQtracts may be a potential protective mechanism against cancer. This theory is based on evidence from studies in Denmark and Sweden who both report a reduction of cancer incidence in HD patients. However, to date, no study has explored the relationship between CAG length and cancer incidence.^2,3^

**Method:** 6540 HD patients were identified using data from the European Huntington’s Disease Registry. The age-standardised incidence ratio (SIR) for specific types of cancer was calculated by comparing risks to general population data from the WHO.^4^

**Results:** 173 of 6540 patients reported a previous history of cancer. The overall SIR for all cancers was 0.26 (0.22-0.30). Each individual type of cancer had a SIR below 1 with statistically significant confidence limits. The average CAG repeat length in cancer patients was 42.06 in comparison to non-cancer patients (44.07). Non-cancer patients were also found to have an earlier average age at HD diagnosis (45.71) in comparison to cancer patients (57.97).

**Conclusion:** This cross-national study provides further evidence to suggest a possible link between HD and a reduction of cancer incidence.^2,3^

Individuals with early-onset HD have a lower cancer incidence in comparison to late-onset HD patients as they experience its protective effects much sooner. Non-cancer patients have longer polyQtracts that protect against cancer by accumulating intracellularly, restricting cellular proliferation and increasing apoptosis.Furthermore, other polyQ diseases such as hereditary ataxia showed a similar reduction in cancer incidence, albeit to a lesser extent.^5^Further research is warranted to investigate these exact mechanisms in the hope to establish the next break-through in cancer research.

Disclosure: The European Huntington's Disease Network is funded by the CHDI Foundation, Inc.: this project was EHDN project 478, and this work was supported by the MRC (MR/L010305/1).

Corresponding author: Paul McNulty

### **95. Mediterranean diet adherence and risk of pancreatic cancer: a pooled analysis of two Dutch cohorts**

#### Maya Schulpen^1^, Petra H. Peeters^2^, Piet A. van den Brandt^1^

##### ^*1*^*Maastricht University*, ^*2*^*University Medical Centre Utrecht*

**Background:** Studies investigating the association of Mediterranean diet (MD) adherence with pancreatic cancer risk are limited and had inconsistent results. We examined the association between MD adherence and pancreatic cancer incidence by pooling individual subject data from two Dutch cohorts.

**Method:** The Netherlands Cohort Study (NLCS, 120852 subjects) and the Dutch cohort of the European

Prospective Investigation into Cancer and Nutrition (EPIC-NL, 40011 subjects) were included in this pooled analysis. MD adherence was assessed using alternate and modified Mediterranean diet scores (aMED and mMED, respectively), including and excluding alcohol. After median follow-ups of 20.3 (NLCS) and 19.2 (EPIC-NL) years, 449 cases of microscopically confirmed pancreatic cancer (MCPC) were included in study-specific multivariable Cox proportional hazards models. Study-specific estimates were pooled using a random-effects model.

**Results:** MD adherence was not significantly associated with MCPC risk in pooled and study-specific analyses, regardless of sex and MD score. Pooled hazard ratios (95% confidence interval) for high (6-8) compared to low (0-3) values of mMED excluding alcohol were 0.66 (0.40 – 1.10) in men and 0.94 (0.63 – 1.40) in women. A two-point increment in mMED excluding alcohol was borderline significantly associated with a reduced MCPC risk in never smokers (p = 0.07), but not in ever smokers (pheterogeneity=0.03). Hazard ratios were consistent across strata of other potential effect modifiers. Considering MD scores excluding alcohol, mMED-containing models generally fitted better than aMED-containing models, particularly in men. Although associations somewhat differed when all pancreatic cancers were considered instead of MCPC, the overall conclusion was similar.

**Conclusion:** MD adherence was not associated with pancreatic cancer risk according to a pooled analysis of two Dutch cohorts.

Disclosure: Funded by Wereld Kanker Onderzoek Fonds Nederland (WCRF-NL); World Cancer Research Fund (WCRF); the Dutch Ministry of Public Health, Welfare and Sports (VWS); the Dutch Prevention Funds; the Dutch ZON (Zorg Onderzoek Nederland)

Corresponding author: Maya Schulpen

### **96. A cluster randomised feasibility study of an adolescent incentive intervention to increase uptake of HPV vaccination**

#### Alice Forster^1^, Victoria Cornelius^2^, Lauren Rockliffe^1^, Laura Marlow^1^, Helen Bedford^1^, Jo Waller^1^

##### ^*1*^*University College London*, ^*2*^*Imperial College London*

**Background:** In England, uptake of the human papillomavirus (HPV) vaccine for the prevention of HPV-related cancers is suboptimal among girls from ethnic minority backgrounds and in some regions, particularly London. As part of the school-based HPV immunisation programme, a consent form with parental signature has to be returned regardless of whether consent for vaccination is given or withheld. Lack of a signed consent form is the main reason for adolescent girls not receiving the vaccine in the UK. We aimed to determine the feasibility of undertaking a cluster randomised controlled trial (RCT) of incentivising consent form return to improve HPV vaccine uptake.

**Method:** An equal-allocation, two-arm cluster RCT design was used. We invited 60 London schools to participate. Those agreeing were randomised to either a standard invitation or incentive intervention arm, in which Year 8 girls had the chance to win a £50 shopping voucher if they returned a vaccination consent form, regardless of whether consent was provided. We collected data on school and parent participation rates, questionnaire response rates, consent form return and vaccine uptake. Analyses were descriptive.

**Results:** Six schools completed the trial and only 3% of parents opted out. The response rate was 70% for the girls’ questionnaire and 17% for the parents’ questionnaire. In the intervention arm, 87% of girls returned a consent form compared to 67% in the standard invitation arm. The proportion of girls whose parents gave consent for vaccination was higher in the intervention arm (76%) than the standard invitation arm (61%).

**Conclusion:** A RCT of an incentive intervention is feasible. This incentive intervention has the potential to substantially improve HPV vaccination uptake, which should reduce HPV-related cancer incidence, with minimal work from immunisation providers, but a fully-powered RCT is needed.

A version of this abstract has been published previously, see 10.1038/bjc.2017.284 for original and CC-BY licence.

Disclosure: Funded by Cancer Research UK

Corresponding author: Alice Forster

### **97. Beliefs about medication and uptake of preventive therapy in women at increased risk of breast cancer: Results from a multi-centre prospective study**

#### Rachael Thorneloe^1^, Rob Horne^2^, Lucy Side^3^, Michael Wolf^4^, Samuel Smith^1^

##### ^*1*^*Leeds Institute of Health Sciences, University of Leeds*, ^*2*^*Centre for Behavioural Medicine, School of Pharmacy, University College London*, ^*3*^*Wessex Clinical Genetics Service, University Hospitals Southampton*, ^*4*^*Division of General Internal Medicine and Geriatrics, Northwestern University*

**Background:** The effectiveness of preventive therapy for breast cancer depends on adequate uptake, but initiation rates remain low. Little is known about factors influencing the decision to use chemoprevention. We examined whether women at increased risk of breast cancer can be categorised into groups with similar medication beliefs and evaluated whether belief group membership was associated with uptake.

**Method:** Women (n = 732) attending an appointment at one of 20 centres in England to discuss breast cancer risk were approached; 55.7% (408/732) completed a survey containing the Beliefs about Medicines Questionnaire (BMQ) and the Perceived Sensitivity to Medicines (PSM) scale. Self-reported uptake of tamoxifen at 3-month follow-up was reported in 258 (63.2%). The optimal number of medication belief groups was identified using Latent Profile Analysis (LPA).

**Results:** Among baseline respondents (mean age = 45.3 years, SD = 7.8), 59.6% were at moderately high risk of breast cancer (17-30% lifetime risk) and 39.0% were at high risk of breast cancer (≥30% lifetime risk). Uptake of tamoxifen was 14.7% (38/258). The LPA model supported a 2-group model. Both groups held weak beliefs about their perceived need for tamoxifen. Group 2 (38% of the sample) reported stronger concerns about tamoxifen and medicines in general, and stronger perceived sensitivity to the negative effects of medicines compared with Group 1 (62%). Women with low necessity and lower concerns (Group 1) were more likely to initiate tamoxifen (18.3%; 33) than those with low necessity and higher concerns (Group 2) (6.4%; 5). After adjusting for demographic and clinical factors, the OR was 3.37 (95% confidence interval: 1.08 – 10.51, *p* = .036).

**Conclusion:** Uptake of breast cancer preventive therapy was low. An important sub-group of women reported low need for preventive therapy and strong medication concerns. These women were less likely to initiate tamoxifen. Medication beliefs are modifiable targets to support informed treatment decision-making.

Disclosure: SGS was supported by a Cancer Research UK postdoctoral fellowship (C42785/A17965) during the collection of these data. He also acknowledges funding support from a Yorkshire Cancer Research University Academic Fellowship.

Corresponding author: Samuel Smith

### **98. A novel application of two-step Mendelian randomization: applying the results of small feasibility studies of interventions to infer causal effects on clinical endpoints**

#### Meda R. Sandu^1^, Rhona Beynon^1^, Rebecca Richmond^1^, Diana L. Santos Ferreira^1^, J Athene Lane^1^, Richard M. Martin^1^

##### ^*1*^*University of Bristol*

**Background:** Feasibility trials are preliminary trials that assess the viability and acceptability of intervention studies and the effects of the intervention on intermediate endpoints. Due to their short duration, they are unable to establish the effects of the intervention on long-term clinical outcomes. We propose a novel method that could transform the interpretation of feasibility trials using modified two-stage randomization analyses.

**Method:** In this two-stage process, we explored the effects of a 6-month feasibility factorial randomised controlled trial (RCT) of lycopene and green tea dietary interventions (ProDiet) on 159 serum metabolic traits in 133 men with raised PSA levels but prostate cancer (PCA) free. In the first stage, we conducted an intention-to-treat analysis, using linear regression to examine the effects of the interventions on metabolic traits, compared to the placebo group. In the second stage, we used a two-sample Mendelian Randomization (MR) approach to assess the causal effect of metabolic traits altered by the interventions, on PCA risk, using summary statistics data from an international PCA consortium of 44,825 cancer cases and 27,904 controls.

**Results:** The systemic effects of lycopene and green tea supplementation on serum metabolic profile were comparable to the effects of the respective dietary advice interventions (R2 = 0.65 and 0.76 for lycopene and green tea respectively). Metabolites which were altered in response to lycopene supplementation were acetate (standard deviation difference versus placebo (β)): 0.69; 95% CI = 0.24, 1.15; p = 0.003), valine (β: -0.62; -1.03, -0.02; p=0.004), pyruvate (β: -0.56; -0.95, -0.16; p = 0.006), and docosahexaenoic acid (β: -0.50; -085, -0.14; p = 0.006). Using MR, a genetically instrumented SD increase in pyruvate increased the odds of PCA by 1.29 (1.03, 1.62; p = 0.027).

**Conclusion:** Using a two-stage randomization analysis in a feasibility RCT, we found that lycopene lowered levels of pyruvate, which our Mendelian randomization analysis suggests may be causally related to reduced PCA risk.

Disclosure: Funded by NIHR, Wellcome Trust, Cancer Research UK

Corresponding author: Meda R. Sandu

### **99. Investigating causal relationships between sleep characteristics and risk of breast cancer: a Mendelian randomization study**

#### Rebecca Richmond^1^, Emma Anderson^1^, Hassan Dashti^2^, Samuel Jones^3^, Jacqueline Lane^2^, Caroline Relton^1^, Marcus Munafò^4^, Debbie Lawlor^1^, Martin Rutter^5^, Richa Saxena^2^, Mike Weedon^3^, Richard Martin^1^, George Davey Smith^1^

##### ^*1*^*University of Bristol*, ^*2*^*Massachusetts General Hospital, Boston*, ^*3*^*University of Exeter*, ^*4*^*University of Bristol, UK*, ^*5*^*University of Manchester*

**Background:** Previous observational studies have identified associations between sleep characteristics and breast cancer. The carcinogenic effect of sleep patterns has also been investigated among night shift workers, with inconsistent findings. Here we conducted a Mendelian randomization (MR) analysis using genetic variants robustly associated with chronotype (morning/evening preference), insomnia and sleep duration to investigate causal links with breast cancer.

**Method:** Observational associations between sleep characteristics and breast cancer in UK Biobank (9,599 cases, 170,616 controls) were first assessed using logistic regression with adjustment for age, assessment centre, ancestry and several socio-economic, lifestyle and reproductive factors. For the MR analysis, breast cancer status was regressed against predicted values of sleep characteristics based on genotype in a logistic regression model, with adjustment for age, assessment centre, ancestry and genotyping chip. Two-sample MR was also conducted using summary data from a large genome-wide association study of breast cancer (122,977 cases, 105,974 controls) conducted by the Breast Cancer Association Consortium (BCAC).

**Results:** In observational analysis, morning preference was inversely associated with breast cancer risk (OR:0.89; 95%CI:0.85, 0.93), insomnia was positively associated (OR:1.21;1.14,1.29) and there was little evidence for an association with sleep duration (OR:1.01;0.99,1.03). Using 351 genetic variants associated with chronotype, 57 with insomnia and 91 with sleep duration, in one-sample MR analysis there was some evidence for a causal effect of morning preference (OR 0.77;0.56,1.05) and weaker evidence for insomnia (OR:1.11;0.70,1.76) and sleep duration (OR:1.14;0.91,1.43). Findings for a protective effect of morning preference (OR 0.93;0.89,0.96) and adverse effect of sleep duration (OR:1.20;1.01,1.41) were supported by two-sample MR using data from BCAC, while there was limited evidence for insomnia (OR:0.97;0.84,1.11).

**Conclusion:** Consistent findings for a protective effect of morning preference on breast cancer risk in both observational and Mendelian randomization analysis support hypotheses around carcinogenic light-at-night, while evidence for a causal effect of sleep disruption is more limited.

Disclosure: Funded by Cancer Research UK, Medical Research Council

Corresponding author: Rebecca Richmond

### **100. Cancer, risk and decision making in vulnerable women: An exploratory study**

#### Sarah Hanson^1^, Duncan Gilbert^2^, Rebecca Landy^3^, Grace Okoli^4^, Cornelia Guell^5^

##### ^1^University of East Anglia, ^2^Brighton and Sussex University Hospitals, ^3^National Cancer Institute, ^4^Queen Mary University London, ^5^European Centre for Environment and Human Health, University of Exeter Medical School, Truro

**Background:** Cancer is associated with socio-economic disadvantage. Yet many interventions designed to reduce risk and improve health fail to reach those with the greatest needs and the most vulnerable. Disadvantaged women, including those who have suffered domestic abuse or who are within the judicial system, represent a group that is particularly poorly accessed in prevention strategies and in research. Our study focused on such disadvantaged women, at two women’s centres that provide support and training.

**Method:** This qualitative study involved thirty participants (23 women and seven staff) in individual interviews and two focus groups. It sought to understand perceptions of, and vulnerability to, cancer; decision making (including screening); cancer symptom awareness and views on health promotion within the context of the women’s daily lives. Verbatim transcripts were analysed thematically.

**Results:** Mental distress dominated our findings. Risk factors of alcohol use, smoking, physical inactivity and unhealthy eating were common but reported within the context of distressing experiences of mental ill-heath, poverty, addition and abuse. Walking, for example, was reported due to lost driving licences or a symptom of anxiety; smoking was reported as part of other additive behaviours such as alcohol abuse. Women’s views of themselves such as self-worth were often negative, shaped by experiences of neglect and abuse. Health-seeking behaviours such as accessing screening services or being aware and presenting with symptoms needed to be understood in the context of highly complex and difficult to navigate, and sometimes even obstructive, health services.

**Conclusion:** Women in this study were at high risk of chronic diseases, including cancer. Their experiences of social disadvantage and lack of control profoundly shaped their practices, aspirations and attitudes towards risk, health and healthcare. Our findings will inform the design of a feasibility study to test a cancer prevention strategy co-designed by and tailored to vulnerable women.

Disclosure: Funded by Cancer Research UK

Corresponding author: Sarah Hanson

### **101. Investigating the effects of dietary and physical activity interventions on the metabolome of men with prostate cancer: The PrEvENT randomised controlled trial**

#### Meda R. Sandu^1^, Diana L. Santos Ferreira^1^, Rebecca Richmond^1^, Lucy Hackshaw-McGeagh^1^, Rhona Beynon^1^, Richard M. Martin^1^, J Athene Lane^1^

##### ^*1*^*University of Bristol*

**Background:** Lycopene, plant-based diets (PBD) and physical activity (PA) have been previously associated with reduced risk and slower progression of prostate cancer (PCA), however, the potential mechanisms are not completely understood.

**Method:** We explored the effects of the PrEvENT randomised controlled trial (RCT) with a 6-month dietary (lycopene supplementation and PBD advice) and brisk walking(BW) intervention on 155 serum metabolites in 74 men with PCA who had undergone prostatectomy, using linear regression and instrumental variable (IV) analysis. One-stage-individual-participant meta-analysis was performed using a subset of data from ProDiet, an RCT of men with raised PSA levels but PCA free who were randomised to lycopene supplements (n=85). The causal effect of the metabolic traits on PCA was assessed by Mendelian Randomization (MR) on 44,825 cancer cases and 27,904 controls in the PRACTICAL consortium.

**Results:** The effects of lycopene supplementation and PBD advice on the serum metabolic profile were comparable (R^2^=.64). There were no strong differences in metabolite levels in either the BW or the dietary intervention. After adjustment for baseline metabolites, there was evidence for decreases of triglycerides in intermediate-density lipoproteins, large, medium and small low-density lipoproteins and saturated fatty acids (p<0.00385) in the BW arm. When accounting for the effect of the dietary intervention on serum lycopene (IV analysis), pyruvate decreased, and acetate increased (p-value<0.05). After pooled meta-analysis with ProDiet, there was strong evidence (p-value<0.004) of decreased pyruvate and alanine, and increased acetate levels, in the lycopene arm. Using genetic instruments, the MR analysis showed evidence for a causal effect of pyruvate on PCA (OR 1.29, 95%CI 1.03-1.62).

**Conclusion:** The interventions to increase lycopene, PBD and PA altered the serum metabolome of men with PCA. BW improved the cardiometabolic profile; lycopene and PBD advice lowered pyruvate, which is known to be involved in cancer mechanisms and may be causally linked to PCA risk.

Disclosure: Funded by NIHR, Wellcome Trust, Cancer Research UK

Corresponding author: Meda R. Sandu

### **102. Genetic determinants underlying the variation in response to cancer chemoprevention agents**

#### Salwa Almayouf^1^, David B.H. Barton^1^, Yue Hu^1^, Edward J. Louis^1^, Steven S. Foster^1^

##### ^*1*^*University of Leicester*

**Background:** Cancer chemoprevention is the use of natural or chemical agents to prevent or delay the development of cancer. However, there is variation in response to chemopreventive agents amongst individuals because it is a complex trait controlled by multiple genes and environmental factors.

Here, we aimed to decipher the genetic factors controlling the variation in response to aspirin, metformin, curcumin and eicosapentaenoic acid using a yeast-based genetic screen before translating findings to humans. This approach was feasible due to the conservation of genes between these two organisms.

**Method:** A multiparent quantitative trait loci (QTL) mapping approach was applied. A panel of 111 F12 meiotic segregants generated from a cross of four S. cerevisiae wildtype isolates were genotyped by whole genome sequencing. Segregants were phenotyped using an automated pipeline which measured yeast growth in different chemopreventive agent treatments. Subsequently, linkage-based fine QTL mapping strategies were performed to locate regions of the genome correlating to the observed phenotype and the identification of causative genes.

**Results:** Linkage analysis has mapped hundreds of genetic loci in the yeast genome responsible for the variation in response to the agents tested. Conserved homologues to human genes with DNA damage repair, histone modification and kinase functions were identified. Some hits have been previously supported in the literature such as the effect of aspirin and metformin on MTOR thus validating this screening approach. Novel genes identified revealed different pathways by which these agents may exhibit their anti-cancer properties.

**Conclusion:** Detection of genetic variants influencing the differences in drug response could help identify individuals at risk or benefit of using chemoprevention agents. Current work includes validating the alleles of causative genes in human cells to assess their biological relevance. This study could aid the development of biomarkers for drug response and validate the repurposing of drugs for prevention of cancer and cancer recurrence.

Disclosure: Funded by Cancer Research UK and Minsitry of Health Kuwait

Corresponding author: Salwa Almayouf

### **103. Effective chemoprevention strategies in APC driven mouse models of intestinal tumourigenesis**

#### Michael Hodder^1^, Patrizia Cammareri^1^, Dennis Timmerman^1^, Owen Sansom^1^

##### ^1^*Beatson Institute for Cancer Research*

**Background:** Truncation of the negative regulator of the Wnt signalling pathway, Adenomatous Polyposis Coli (APC), represents one of the earliest commonly occurring events in Colorectal Cancer (CRC) progression. In addition, a number of factors substantially increase the risk of developing CRC, including inherited mutations in APC, such as Familial Adenomatous Polyposis (FAP). Here we exploit vulnerabilities in APC mutant stem cells and use chemo-preventative strategies to influence tumour initiation in the mouse intestinal epithelium.

**Method:** Using in vivo mouse models we mutate APC specifically in the Lgr5+ve stem cell population of the intestine. We subsequently challenge these models with short term therapeutic strategies to influence early lesions, and assess the ability of chemotherapy to influence mutant clone establishment, as well as tumour formation and overall survival.

**Results:** We demonstrate that APC deficient intestinal stem cells are more sensitive to chemotherapy when compared with wild type stem cells. In addition, we establish that short term therapeutic interventions, including those which cause DNA damage and those which specifically target apoptotic machinery, are able to influence tumour initiation, extend survival and reduce tumour numbers in mouse models. Furthermore, we identify that timing is crucial for therapeutic efficacy, as treatment of established tumours has no significant impact on tumour progression.

**Conclusion:** Overall we provide evidence that chemoprevention is a tenable approach to combat CRC, and could be of significant benefit to high risk patients, such as those with FAP. Similar to our mouse models, chemoprevention could offer more tangible patient benefit than treatment of established tumours. This work highlights that early lesions are exquisitely sensitive to a variety of therapies and that mutant clones can be eliminated and readily replaced with healthy stem cells prior to tumour formation.

Disclosure: Funded by Cancer Research UK and Medical Research Council

Corresponding author: Michael Hodder

## Treatment

### **104. Real-world evidence of the effectiveness of adjuvant chemotherapy for early stage breast cancer from Scottish routine data**

#### Ewan Gray^1^, Joachim Marti^2^, David Brewster^1^, Jeremy Wyatt^3^, Peter Hall^1^

##### ^*1*^*University of Edinburgh*, ^*2*^*University of Lausanne*, ^*3*^*University of Southampton*

**Background:** Real world evidence (RWE) has been proposed as a means to address the ‘efficacy-effectiveness gap’ in evidence-based oncology. A major barrier to use of RWE is the perceived lack of internal validity arising from the increased potential for bias compared to evidence generated from randomised trials. This study tested the internal validity of RWE methods applied to estimating to effectiveness of adjuvant chemotherapy for early breast cancer.

**Method:** Data were obtained from the Scottish cancer registry. All cases of primary breast cancer from 2001 to 2015 were extracted for analysis. Follow-up of vital status until April 2016 was provided by linkage of death records to the cancer registry. Cases meeting the eligibility criteria for adjuvant chemotherapy trials were selected based on age, co-morbidity and prognostic factors. Hazard ratios (HRs) estimating the effectiveness of chemotherapy for preventing breast cancer specific and all-cause mortality, in this trial-eligible group, were calculated using Cox regression on a propensity score matched sample. The HRs were compared with estimates from a published individual patient data meta-analysis of all adjuvant therapy randomised trials.

**Results:** 13,560 trial eligible cases were selected from 56,565 individual records. The primary estimate of the HR for adjuvant chemotherapy for breast cancer specific mortality was 0.679 (95%CI: 0.678, 0.681). In comparison, the meta-analysis reported a HR of 0.64 (95%CI: 0.585, 0.700) for the chemotherapy regimens most commonly used in Scotland during this period. The HR varied between trial-ineligible subgroups.

**Conclusion:** The RWE and meta-analysis of randomised trials provide results that are consistent within an acceptable degree of precision. This suggests RWE estimates of effectiveness have internal validity in the adjuvant therapy setting for early breast cancer. Future studies should consider RWE methods in this setting to address specific gaps in the evidence base provided by clinical trials

Acknowledgement: A version of this abstract has been published previously, see http://abstracts.asco.org/214/AbstView_214_226519.html for original and CC-BY license.

Disclosure: Funded by Chief Scientist Office

Corresponding author: Peter Hall

### **105. Radiotherapy (RT) to the primary tumour for men with newly-diagnosed metastatic prostate cancer (PCa): Survival results from STAMPEDE (NCT00268476)**

#### Christopher C Parker^1^, Nicholas D James^2^, Chris Brawley^3^, Noel W Clarke^4^, Gerhardt Attard^5^, Simon Chowdhury^6^, Bill Cross^7^, Clare Gilson^3^, Robert J Jones^8^, Robin Millman^9^, David P Dearnaley^10^, Malcolm D Mason^11^, Alastair Ritchie^9^, J Martin Russell^12^, Syed Adnan Ali^13^, Chinnamani Eswar^14^, Joanna Gale^15^, Alex Hoyle^13^, Jason Lester^16^, Denise Sheehan^17^, Anna Tran^13^, Silke Gillessen^18^, Claire Amos^3^, Mahesh KB Parmar^3^, Matthew R Sydes^3^ on behalf of The STAMPEDE investigators working group

##### ^*1*^*Institute of Cancer Research, Sutton; Royal Marsden Hospital, Sutton*, ^*2*^*Institute of Cancer and Genomic Sciences, University of Birmingham, Edgbaston, Birmingham*, ^*3*^*Medical Research Council Clinical Trials Unit at University College London*, ^*4*^*Christie and Royal Salford Hospital, Manchester*, ^*5*^*University College London Cancer Institute*, ^*6*^*Guy's and St Thomas' NHS Foundation Trust*, ^*7*^*St James University Hospital*, ^*8*^*Institute of Cancer Sciences, University of Glasgow, Glasgow; Beatson West of Scotland Cancer Centre, University of Glasgow, Glasgow*, ^*9*^*c/o Medical Research Council Clinical Trials Unit at University College London*, ^*10*^*Institute of Cancer Research, Sutton*, ^*11*^*University of Cardiff*, ^*12*^*Institute of Cancer Sciences, Beatson West of Scotland Cancer Centre, University of Glasgow, UK*, ^*13*^*Christie Hospital*, ^*14*^*Royal Liverpool University Hospital*, ^*15*^*Queen Alexandra Hospital*, ^*16*^*Weston Park Hospital*, ^*17*^*Royal Devon and Exeter NHS Foundation Trust*, ^*18*^*Division of Oncology and Hematology, Kantonsspital St. Gallen*

**Background:** Primary tumours in men with metastatic PCa could contribute to overall disease progression and shorter survival. To test this hypothesis, we evaluated whether RT to the prostate improves overall survival in men presenting with metastatic PCa.

**Method:** STAMPEDE is a multi-arm multi-stage platform protocol which included a randomised phase III comparison to test the above hypothesis. Standard-of-care (SOC) was lifelong ADT, with 6*3-weekly docetaxel permitted from 2016. Stratified randomisation allocated pts 1:1 to SOC or SOC+RT. Pts allocated to RT received daily (55Gy/20f over 4 weeks) or weekly (36Gy/6f over 6 weeks) RT according to investigators’ pre-specified (before randomisation) choice. Randomisation was ≤12 weeks on ADT; RT started ≤8 weeks after randomisation or docetaxel. The primary outcome measure (OM) was death from any cause; secondary efficacy OMs included failure-free survival (FFS) & symptomatic local event-free survival (SLEFS). Comparison to control for survival had 90% power at 2.5% 1-sided alpha for hazard ratio (HR) of 0.75, requiring ~267 control arm deaths, accounting for 3 intermediate lack-of-benefit analyses on FFS. Analyses used Cox proportional hazards & flexible parametric models, adjusted for stratification factors. Subgroup analyses will show effects by disease volume at entry and planned RT schedule.

**Results:** 2061 pts from UK & Switzerland with newly-diagnosed M1 PCa were contemporaneously randomised to the 2 arms (Jan2013 – Sep2016). Randomised groups were well balanced: median age 68yrs; median PSA 97ng/ml; 18% early docetaxel; pre-specified RT schedule: 52% daily, 48% weekly; volume 39% low, 54% high, 8% pending/NK.

**Conclusion:** The pending results from this large randomised comparison will define the role of RT to the primary tumour in men with newly-diagnosed M1 PCa.

(CTU follows a controlled access approach for data sharing. The data are available after independent review to bona fide researchers following a formal application. They are not freely available because we have sensitive personal information. A data release request can be initiated by submitting an email to mrcctu.stampede@ucl.ac.uk or the CTU Enquiries address.)

Disclosure: Funded by Cancer Research UK -- dedicated grant; Medical Research Council -- core trials unit funding; Contribution to the platform protocol from Astellas, Clovis, Janssen, Pfizer and Sanofi-Aventis.

Corresponding author: Christopher C Parker

### **106. A Phase I dose-finding and safety study of AZD8931 with Oxaliplatin and Capecitabine chemotherapy in patients with Oesophago-gastric adenocarcinoma: the randomised expansion phase of the DEBIOC trial**

#### Anne Thomas^1^, Pradeep S. Virdee^2^, Martin Eatock^3^, Simon Lord^4^, Stephen Falk^5^, D. Alan Anthoney^6^, Richard Turkington^7^, Matthew Goff^8^, Leena Elhussein^2^, Linda Collins^8^, Joanna Moschandreas^2^, Mark Middleton^4^

##### ^*1*^*University of Leicester*, ^*2*^*Centre for Statistics in Medicine, University of Oxford*, ^*3*^*Belfast City Hospital*, ^*4*^*University of Oxford*, ^*5*^*Bristol Haematology & Oncology Centre*, ^*6*^*St. James University Hospital*, ^*7*^*Centre for Cancer Research and Cell Biology, Queens University Belfast*, ^*8*^*Oncology Clinical Trials Office, University of Oxford*

**Background:** AZD8931 is a novel small-molecule inhibitor with equipotent activity against EGFR, erbB2 (HER2), and erbB3 signalling. The recommended Phase 2 dose (RP2D) of AZD8931 with Oxaliplatin and Capecitabine (XELOX), determined in the DEBIOC escalation phase, is 20mg twice-daily, 4 days on, 3 days off, in a 3-week cycle. The dose expansion phase aims to give a preliminary efficacy assessment and further investigate the safety and tolerability of AZD8931+XELOX.

**Method:** Following written informed consent, thirty patients with operable oesophago-gastric adenocarcinoma (OGC) were randomised 2:1 to receive XELOX with AZD8931 (n = 20) at the RP2D or XELOX only (n = 10) for two 3-week cycles, with radical oesophago-gastric surgical resection 4-6 weeks later. Patients on neo-adjuvant AZD8931 could have maintenance AZD8931 6-12 weeks after complete (R0) or margin positive (R1) resection. Expansion phase outcomes analysed by intention-to-treat included 6-month progression-free survival (PFS), R0 rate, and overall survival.

**Results:** The median age was 64 (range 25-78) years and the median follow-up 26.8 (range 4.5-32.5) months. Six-month PFS was 85% (90% CI 66%-94%) in the AZD8931+XELOX and 100% in the XELOX-only group. R0 rate was 45% in the AZD8931+XELOX and 90% in the XELOX-only group (p=0.024). Seven disease-related deaths (35%) occurred in the AZD8931+XELOX group, one (10%) in the XELOX-only group.

In the neo-adjuvant period, 10% of AZD8931+XELOX and 50% of XELOX-only patients had ≥1 grade 3-4 adverse events (AE). Thirteen serious AEs (SAEs; 7 AZD8931+XELOX, 6 XELOX-only) were reported. Two SUSARs were reported: thoracotomy wound dehiscence (AZD8931+XELOX) and hypophosphataemia (XELOX-only). During maintenance, two patients (18%) had a grade 3 AE; there was one non-treatment related SAE.

**Conclusion:** AZD8931+XELOX has an acceptable safety profile but no early signal of superior activity compared to standard treatment in operable OGC patients was demonstrated.

Clinical Trials Registry: 12/SC/0090- http://www.isrctn.com/search?q=12%2FSC%2F0090

Disclosure: University of Oxford served as sponsor and AstraZeneca and CRUK (an ECMC/AstraZeneca alliance trial) funded the work.

Corresponding author: Leena Elhussein

### **107. SCALOP-2: A randomised trial of induction GEMABX followed by chemoradiation (high/standard dose radiation) +/-nelfinavir for locally advanced pancreatic cancer: results of the dose-finding component**

#### Somnath Mukherjee^1^, Pradeep S. Virdee^2^, Rachel Shaw^3^, John Bridgewater^4^, Ganesh Radhakrishna^5^, Stephen Falk^6^, Martin Scott-Brown^7^, Victoria Y. Strauss^2^, Claire Brookes^3^, Roopinder Gillmore^8^, Neel Patel^9^, Bethan Tranter^10^, Phil Parsons^11^, David Sebag-Montefiore^12^, Maria Hawkins^1^, Pippa Corrie^13^, Tim Maughan^1^

##### ^*1*^*Department of Oncology, University of Oxford, Oxford, UK*, ^*2*^*Centre for Statistics in Medicine, University of Oxford, Oxford, UK*, ^*3*^*Oncology Clinical Trials Office, University of Oxford, Oxford, UK*, ^*4*^*Department of Oncology, University College London Hospitals, London, UK*, ^*5*^*Oncology Department, The Christie NHS Foundation Trust, Manchester, UK*, ^*6*^*Bristol Haematology and Cancer Centre, Bristol University Hospitals NHS Foundation Trust, Bristol, UK*, ^*7*^*Arden Cancer Centre, University Hospitals Coventry & Warwickshire, Coventry, UK*, ^*8*^*Academic Oncology, Royal Free Hospital NHS Foundation Trust, London, UK*, ^*9*^*Department of Radiology, Churchill Hospital, Oxford, UK*, ^*10*^*Pharmacy Department, Velindre Cancer Centre, Velindre NHS Trust, Cardiff, UK*, ^*11*^*Cardiff NCRI RTTQA group, Department of Medical Physics, Velindre Cancer Centre, Cardiff, UK*, ^*12*^*University of Leeds, Leeds Cancer Centre, St James's University Hospital, Leeds UK*, ^*13*^*Oncology Centre, Addenbrooke's Hospital, Cambridge, UK*

**Background:** The anti-retroviral agent, nelfinavir, demonstrates radiosensitising effects in pre-clinical models of pancreatic cancer. The primary objective of Stage 1 was to establish the maximum tolerated dose (MTD) of nelfinavir combined with capecitabine-chemoradiation (CRT) after gemcitabine+nab-paclitaxel (GEMABX) induction chemotherapy. Other outcomes included overall survival and progression-free survival.

**Method:** Patients with inoperable, histologically/cytologically proven locally advanced pancreatic cancer (LAPC) and WHO performance status 0-1 were eligible for this rolling-six dose-escalation stage. After 3 cycles of induction GEMABX (28-day cycle of nab-paclitaxel 125mg/m^2^ and gemcitabine 1000mg/m^2^ on days 1, 8, and 15), patients with non-progressive disease had 1 further cycle followed by CRT (50.4Gy/28 fractions, capecitabine 830mg/m^2^ bd on radiotherapy days) and 1000mg or 1250mg nelfinavir bd continuously during CRT.

**Results:** 27 patients were recruited from 8 UK centres (March 2016-June 2017). Median age was 62 years, 30% were male, 78% had head tumours, and 30% had biliary stents. Baseline median tumour diameter was 36mm.

67% commenced CRT. 11 patients received 1000mg and there was one dose-limiting toxicity (DLT) in this group: grade 3 acute coronary syndrome. The nelfinavir dose was escalated as per the rolling-six design. 7 patients received 1250mg nelfinavir and no DLTs were observed.

During GEMABX, common grade ≥3 toxicities among participants were neutropenia (30%), fatigue (22%), and diarrhoea (15%). During CRT, grade ≥3 toxicities included fatigue (6%) and anorexia (6%). No grade 5 adverse events were reported in Stage 1.

Survival analysis will be presented.

**Conclusion:** 1250mg nelfinavir was recommended for combining with capecitabine-CRT in the ongoing randomised component of the trial (Stage 2).

Disclosure: Funded by Cancer Research UK and Celgene Limited

Corresponding author: Pradeep S. Virdee

### **108. Molecular characterisation of lung adenocarcinoma using a 22-gene panel: A United Kingdom tertiary cancer centre experience**

#### Kam Zaki^1^, Doraid Alrifai^1^, Danny Ulahannan^1^, Tanya Ahmad^1^, Dionysis Papadatos-Pastos^1^, Siow Ming Lee^1^, Charles Swanton^1^, Phil Bennett^2^, Mary Falzon^1^, Elaine Borg^1^, Neal Navani^1^, Sam Janes^1^, Martin Forster^1^

##### ^*1*^*University College London Hospitals NHS Foundations Trust*, ^*2*^*Sarah Cannon Molecular Diagnostics*

**Background:** Several licenced therapies targeting somatic driver aberrations in lung adenocarcinoma have demonstrated superior outcomes compared to chemotherapy. Incorporating molecular panels into diagnostic services is imperative to identify targetable driver events and improve patient access to these agents. The Thoracic Oncology Unit at University College London Hospitals (UCLH) utilised a 22-gene next generation sequencing (NGS) panel in addition to other standardised diagnostic tools such as immunohistochemistry (IHC) and fluorescence in situ hybridisation (FISH).

**Method:** Between September 2014 to April 2016, 228 lung cancer patient tumour samples were subjected to 22-gene panel sequencing at UCL Advanced Diagnostics. Samples also underwent IHC staining for ALK translocations, with equivocal IHC ALK results confirmed by FISH. Clinical data were collected from UCLH electronic records system e-CareLogic (CDR).

**Results:** NGS results for 190 patients with lung adenocarcinoma were available, of which 164 patients harboured somatic variants. 136 unique variants of 20 genes were identified. The most common variants detected were TP53, EGFR and KRAS in 128 (67.3%), 43 (22.6%) and 32 (16.8%) patients respectively. MET amplifications were detected in 2 patients. 24 unique EGFR variants were detected of which 15 were known sensitising variants. 39 patients harboured an EGFR sensitising variant, including 24 metastatic patients.

Patients with sensitising EGFR variants who received first line EGFR TKIs (tyrosine kinase inhibitors) have a median overall survival (OS) of 21 months compared to 6.5 months for those who did not . ALK translocation was detected in 3 (1.7%) patients. The two patients who received ALK TKI are still alive (OS 28 and 32 months respectively). 5 (2.6%) patients went on to participate in targeted therapy clinical trials.

**Conclusion:** Defining the genomic landscape of lung adenocarcinoma personalises treatment options and improves patient outcomes. Newly developed diagnostic platforms such as circulating free DNA are under continuous evaluation and incorporated into standard clinical practice.

Disclosure: None declared

Corresponding author: Kam Zaki

### **109. Optimising the use of platinum chemotherapies in combination with the ATR inhibitor, AZD6738, in breast cancer**

#### Charlie Huins^1^, Sally Hall^2^, Christopher Ottley^3^, Gareth Veal^2^, Yvette Drew^2^

##### ^*1*^*Independant Researcher*, ^*2*^*Newcaslte University*, ^*3*^*Durham University*

**Background:** Platinum chemotherapy has an evolving role in the treatment of BRCA mutant and triple negative breast cancers (TNBC). Platinums (Pt) exert their cytotoxic effect by forming DNA crosslinks (Pt-DNA adducts), which impair DNA function if left unrepaired. AZD6738 is an oral inhibitor of ataxia telangiectasia and rad-3-related (ATR) protein kinase, a key component of the DNA damage response (DDR). ATR signals to prevent the onward transmission of DNA damage at cell division. Whilst the rationale for combining AZD6738 with Pt is one of potential enhanced efficacy, a greater understanding of the mechanism of AZD6738-Pt interaction, particularly with regards to the formation and repair of Pt-DNA adducts, may help guide the optimum dose and scheduling in the clinic.

**Method:** The cytotoxicity of AZD6738 alone, and in combination with cisplatin was assessed by clonogenic survival assay in three human breast cell lines: MCF7 (BRCA1 heterozygous), HCC1806 (TNBC, BRCA wildtype) and T47D (BRCA wildtype). Pt-DNA adduct formation in MCF7 cells treated with cisplatin and AZD6738 was measured by inductively-coupled plasma mass spectrometry (ICP-MS).

**Results:** Sensitivity to AZD6738 monotherapy varied: LC50s ranged from 0.25µM +/- 0.07 in MCF7 to 0.64µM +/- 0.04 in HCC1806. AZD6738 in combination with cisplatin was synergistic in MCF7 and HCC1806 cells. Neither pre-exposure, co-exposure nor post-exposure of AZD6738 in relation to cisplatin affected total numbers or longevity of Pt-DNA adducts formation in MCF7 cells.

**Conclusion:** The synergistic action of AZD6738 with cisplatin in non-pathogenic BRCA mutant cells supports their combination in the clinic. However, more work is required to understand the mechanism of interaction. To this end, this synergistic effect is under investigation in additional cell lines. Investigation of baseline expression of key DDR proteins as a determinant of AZD6738 sensitivity is ongoing and will be presented.

Disclosure: Funded by Newcastle University

Corresponding author: Charlie Huins

### **110. Developing new paradigms for overcoming drug resistance in cancer using novel humanised mouse models**

#### Colin Henderson^1^, Yury Kapelyukh^1^, Kenneth MacLeod^2^, De Lin^1^, Aileen McLaren^1^, Roland Wolf^1^

##### ^*1*^*University of Dundee*, ^*2*^*Concept Life Sciences*

**Background:** Use of molecularly-targeted agents in cancer treatment has significantly improved patient survival, but is severely constrained by toxicity and rapid onset of drug resistance. This can be attributed in part to the high potency of such drugs, which are used at doses (close to MTD), which can be several orders of magnitude > IC_50_. A key question is whether treatment regimens can be changed to retain efficacy and reduce the onset of drug resistance, greatly facilitating the development of effective combination treatments. Such studies require new animal models which more closely reflect human responses to drugs.

**Method:** To address the limitations of mouse models in predicting drug efficacy in man, we developed transgenic mice nulled or humanised for the key P450 enzymes involved in drug disposition in man. We have characterised these models and carried out pharmacokinetic and drug/drug interaction studies with targeted anti-cancer drugs.

**Results:** Whereas murine Cyp2d enzymes metabolise the EGFR inhibitor osimertinib, we have shown that CYP3A4 is the major human enzyme involved, also uncovering a novel pathway of disposition involving human CYP1A1. Preliminary data using our 8HUM model - where 32 mouse P450s are replaced with six human enzymes accounting for >90% of P450-catalysed drug metabolism in man - found in vivo PK of the BRAF inhibitor dabrafenib to closely match that in patients, and that induction or inhibition of CYP3A4, the major human P450 involved in dabrafenib disposition, significantly alters dabrafenib PK. We also demonstrated that dabrafenib is a potent CYP3A4 inducer which will markedly affect its own disposition and that of co-medications.

**Conclusion:** We have validated a powerful new in vivo model system for determining PK/PD relationships for novel drug combinations and for the study of drug resistance. These models have significant potential in the personalisation of cancer treatment using patient-derived xenografts.

Disclosure: Funded by Cancer Research UK, Medical Research Council

Corresponding author: Colin Henderson

### **111. Results of CALIBER: A phase II randomised feasibility trial of chemoablation with MMC versus surgical management in low risk non-muscle invasive bladder cancer (NMIBC)**

#### Hugh Mostafid^1^, Joanne Cresswell^2^, Leyshon Griffiths^3^, John Kelly^4^, Allen Knight^5^, James Catto^6^, Kim Davenport^7^, Andrew Feber^8^, Margaret Knowles^9^, John McGrath^10^, Peter Cooke^11^, Shikohe Masood^12^, Aicha Goubar^13^, Steven Penegar^13^, Nuria Porta^13^, Laura Wiley^13^, Rebecca Lewis^13^, Emma Hall^13^

##### ^*1*^*Royal Surrey County Hospital NHS Foundation Trust*, ^*2*^*South Tees Hospitals NHS Foundation Trust*, ^*3*^*University Hospitals of Leicester NHS Trust*, ^*4*^*University College London Hospital*, ^*5*^*Patient Representative*, ^*6*^*University of Sheffield*, ^*7*^*Gloucestershire Hospitals NHS Foundation Trust*,, ^*8*^*University College London*, ^*9*^*Leeds Institute of Cancer and Pathology*, ^*10*^*Royal Devon and Exeter NHS Foundation Trust*, ^*11*^*The Royal Wolverhampton Hospitals NHS Trust*, ^*12*^*Medway NHS Trust*, ^*13*^*Institute of Cancer Research*

**Background:** Mitomycin C (MMC) chemotherapy has a well-defined safety profile and is used in the treatment of intermediate and high risk NMIBC. CALIBER aimed to demonstrate that intravesical MMC (chemoablation) had sufficient activity to warrant further investigation as an alternative to surgery for low risk NMIBC.

**Method:** CALIBER has a Simon two-stage design, incorporating a surgical control group to test feasibility of randomisation. Patients with recurrent low risk NMIBC were randomised 2:1 to chemoablation (4x 40mg weekly MMC) vs. surgery (standard of care). The primary endpoint was complete response (CR) to chemoablation by visual assessment and histological biopsy at 3 months post-treatment, aiming at excluding CR rate <45% (Stage 1). Quality of life (QoL) at 3, 6 and 12 months post-treatment was measured using EORTC QLQ-C30 and QLQ-NMIBC24.

**Results:** 82 participants (54 chemoablation, 28 surgery) were recruited from 37 UK centres (28/01/2015 to 04/09/2017). Feasibility of randomisation was demonstrated with acceptance rates of 55%. Stage 1 CR criterion was not met and the trial closed to recruitment in September 2017. Estimated 3 month CR rate is 37.0% (95% CI: 24.3, 51.3) in the chemoablation group and 80.8% (95% CI: 60.6, 93.4) in the surgery group. No grade 3-4 toxicities were reported in either group. 70/78 (89.7%) patients participated in the optional QoL sub-study and completed baseline questionnaires (49 chemoablation, 21 surgery). 6-months mean change from baseline in QLQ-C30 global health status was +1.5 surgery vs -4.8 chemoablation (no statistically significant difference).

**Conclusion:** Chemoablation with MMC is safe, however it did not meet pre-specified activity levels to pursue further investigation. Whilst surgery appears more effective in this setting, the proportion of patients with residual disease at 3 months suggests surgery alone may be suboptimal. Further research is required to determine the role of chemoablation with other agents in patients with low risk NMIBC.

Disclosure: Funded by National Institute for Health Research

Corresponding author: Laura Wiley

### **112. 12 week PET-CT scans post-radiotherapy for Human Papillomavirus (HPV) positive Oropharygeal Squamous Cell Cancers (OPSCC) have low positive predictive value (PPV) for lymph node (LN) residual disease**

#### Robert Rulach^1^, Suyun Zhou^1^, Fraser Hendry^2^, David Stobo^2^, Mary Frances Dempsey^2^, Derek Grose^1^, Carolynn Lamb^1^, Allan James^1^, Stefano Schipani^1^, Christina Wilson^1^, Mohammed Rizwanullah^1^, Claire Paterson^1^

##### ^*1*^*The Beatson West of Scotland Cancer Centre*, ^*2*^*NHS Greater Glasgow and Clyde*

**Background:** The PET-NECK study showed that a complete metabolic response on PET-CT 12 weeks after radiotherapy (RT) spared neck dissections (ND) with no resultant reduction in survival. As HPV-positive OPSCC respond later on anatomical imaging, it remains unclear whether an immediate ND is necessary for patients with equivocal responses on the 12 week PET-CT (12wk PET-CT).

**Method:** 12wk PET-CT scans of patients treated with RT/ChemoRT for OPSCC were evaluated retrospectively by a radiologist to categorise incomplete, equivocal or complete responses (IR/EQR/CR) in LNs. Patient details were obtained from electronic records.

**Results:** 154 patients treated with chemo/radiotherapy were identified (116 males, 38 females, median age 58 (range 39-78)). HPV status was as follows: HPV-positive (126), HPV-negative (21), HPV-unknown (7). Median follow up was 24.4 months (range 3–52 months). 38 patients (24.7%) had an EQR. 17 EQR patients (44.7%), all HPV-positive, had a second PET-CT scan at a median of 90 days after the 12wk PET-CT. These scans showed 12 late CRs (70.6%), 2 continued EQRs (both are recurrence-free) and 3 late IRs (two patients were recurrence-free, one had distant metastases). For HPV-positive patients, the PPV and the negative predictive value (NPV) of the 12wk PET-CT is 27.9% and 97.4% respectively, derived from relapse data in Table 1. For HPV-negative patients, the PPV and NPV are 77.8% and 87.5% respectively.

**Conclusion:** 12wk PET-CT scans have a high NPV for residual/recurrent disease OPSCC regardless of HPV status. The PPV of an IR/EQR for HPV-positive OPSCC is low so the optimal surveillance/salvage strategy for these patients requires further clarification.

Disclosure: None declared

Corresponding author: Robert Rulach

Table 1 [Abstract 112]. 12wk PET-CT responses and relapse by HPV status. *Includes distant metastases and residual disease on ND

**Table:** 

HPV-positive			
	No Relapse	Relapse*	Total
CR	75	2	77
EQR/IR	31	12	43
	106	14	120
HPV-negative			
	No Relapse	Relapse*	
CR	7	1	8
EQR/IR	2	7	9
	9	8	17

### **113. PET-CT surveillance post radiotherapy in advanced head and neck squamous cell cancer (HNSCC) - real life application of the PET-Neck protocol**

#### Suyun Zhou^1^, Robert Rulach^1^, Fraser Hendry^1^, David Stobo^1^, Mary Frances Dempsey^1^, Derek Grose^1^, Carolynn Lamb^1^, Allan James^1^, Stefano Schipani^1^, Mohammed Rizwannullah^1^, Christina Wilson^1^, Claire Paterson^1^

##### ^1^*Greater Glasgow and Clyde NHS Scotland*

**Background:** The PET-NECK study demonstrated surveillance CT-PET scan 12 weeks post-radiotherapy for HNSCC was non-inferior to planned neck dissection (ND). We evaluated this practice in our centre.

**Method:** Patients with node positive HNSCC treated with radiotherapy between January 2013 and September 2016 were identified from the PET-CT database. CT-PET responses were classified retrospectively as complete (CR), incomplete (ICR) or equivocal (EQR) by a radiologist. Patient demographics and clinical outcomes were obtained from electronic patient records.

**Results:** 187 patients with HNSCC were identified, 74.8% male, mean age 59 years. 8%(15/187) with N2a, 57%(107/187) with N2b, 16%(29/187)with N2c, and 5%(9/187)with N3 disease. 80.2% received chemoradiotherapy. 81%(154/187) of patients had oropharyngeal cancer, 80.5%(124/154) were HPV-positive. Median follow-up was 30 months (IQR 21.6-39.7). Median time from end of radiotherapy to PET scan was 90days.

59.4%(111/187) had CR, 17.6%(33/187) ICR, and 23%(43/187) EQR nodal response. 21(11%) NDs were performed, 57.1% were pathologically positive. 1-year recurrence was 9.3%, 29.4% and 7.7% for CR, ICR and EQR groups respectively (p=0.04). 2 year survival was 91.9%, 50.0% and 87.5% respectively (p<0.001). No statistical differences in recurrence and survival rates between CRs and EQR at 1-year and 2-years. Only 10 NDs were carried out for the EQR subset with 50% pathological involvement. 20 patients with EQR underwent a repeat PET resulting in a further 12 CRs, suggesting the comparable outcomes between EQRs and CRs are not due to salvage ND and more likely to be related to slowly responding disease.

Overall locoregional control at 2 years was 94.8%(95% CI, 89.6-97.9) and 2 years survival was 83.5%(95% CI, 76.6-88.3).

**Conclusion:** Real life application of the PET-NECK protocol has resulted in similar outcomes to that seen in the study. Most patients are spared ND and disease control is maintained with PET-CT surveillance post-radiotherapy.

Disclosure: Funded by Beatson West of Scotland

Corresponding author: Suyun Zhou

### **114. Aspirin use after radical cancer therapy – feasibility and toxicity data from the Add-Aspirin trial**

#### Nalinie Joharatnam^1^, Fay Cafferty^1^, Alistair Ring^2^, Howard Kynaston^3^, Richard Wilson^4^, Duncan Gilbert^5^, David Cameron^6^, Farhat Din^6^, Richard Hubner^7^, Anne Thomas^8^, Daniel Swinson^9^, Janusz Jankowski^10^, Sam Rowley^11^, Martin Scott-Brown^12^, Chris Price^13^, Alex Walther^14^, David Eaton^15^, Nicola Ainsworth^16^, Rachel Kerr^17^, Luke Hughes-Davies^18^, Max Parmar^1^, Conjeeveram Pramesh^19^, Sudeep Gupta^19^, Ruth Langley^1^

##### ^*1*^*MRC Clinical Trials Unit at UCL*, ^*2*^*The Royal Marsden NHS Foundation Trust*, ^*3*^*Cardiff and Vale University Health Board*, ^*4*^*Queen's University Belfast*, ^*5*^*Brighton and Sussex University Hospitals NHS Trust*, ^*6*^*The University of Edinburgh*, ^*7*^*The Christie NHS Foundation Trust*, ^*8*^*University Hospitals of Leicester*, ^*9*^*Leeds Teaching Hospitals NHS Trust*, ^*10*^*University of Central Lancashire*, ^*11*^*Medical Research Council Clinical Trials Unit at University College London*, ^*12*^*University Hospitals of Coventry and Warwickshire NHS Trust*, ^*13*^*Worcestershire Acute Hospitals NHT Trust*, ^*14*^*University Hospitals Bristol NHS Foundation Trust*, ^*15*^*University Hospitals of Morecambe Bay NHS Foundation Trust*, ^*16*^*The Queen Elizabeth Hospital King's Lynn NHS Foundation Trust*, ^*17*^*Oxford University Hospitals NHS Trust*, ^*18*^*Cambridge University Hospitals NHS Foundation Trust*, ^*19*^*Tata Memorial Centre*

**Background:** Pre-clinical, observational and randomised evidence suggests aspirin may prevent or delay the development of cancer and metastases, and is strongest for colorectal and gastro-oesophageal cancer. However, concerns around feasibility, adherence and tolerability (particularly serious bleeding) have limited aspirin use for cancer chemoprevention.

**Method:** Add-Aspirin is a double-blind, randomised-controlled trial encompassing 4 individually powered phase III trials in early-stage breast, prostate, colorectal and gastro-oesophageal cancer, evaluating the effect of aspirin after radical therapy. All participants initially take open-label aspirin (100mg daily) for 8 weeks (run-in), to assess adherence and toxicity prior to randomisation (1:1:1, aspirin 300mg, aspirin 100mg or matched placebo for ≥5 years). A pre-planned feasibility analysis was performed to assess tolerability and adherence when >2000 participants had completed the run-in period.

**Results:** Between October 2015 and October 2017, 3494 of a targeted 11000 participants were registered from 165 sites in the UK; recruitment rates differed across tumour sites compared to predictions. Run-in data (n=2253) showed good adherence: 95% took 6-7 tablets/week and 85% proceeded to randomisation, with rates consistent across tumour cohorts. Main reasons for not proceeding to randomisation were toxicity (mostly minor, grade 1/2) and/or patient choice, with only 0.7% (16/2253) of participants experiencing toxicity requiring discontinuation during the run-in. Fewer than 1% (13/2253) experienced a grade > 3 toxicity (including one lower gastrointestinal bleed in a prostate cancer participant; no upper gastrointestinal bleeds).

**Conclusion:** The data demonstrate that aspirin is well-tolerated over an 8-week run-in, and acceptable to patients after radical cancer therapy, with low toxicity rates in all tumour cohorts, including gastro-oesophageal participants. A run-in approach may be useful in adjuvant (or prevention) studies for reducing the risk of non-adherence and participant attrition at a later date. Trial recruitment continues with >5000 participants now registered from the UK and India.

Disclosure: Funded by Cancer Research UK (C471 /A15015); the National Institute for Health Research Health Technology Assessment Programme (12/01/38); MRC Clinical Trials Unit at University College London; Sir Dorabji Tata Trust

Corresponding author: Nalinie Joharatnam

### **115. FAST-Forward phase 3 RCT of 1-week hypofractionated breast radiotherapy: 3-year normal tissue effects**

#### A Murray Brunt^1^, Joanne Haviland^2^, Mark Sydenham^2^, Abdulla Alhasso^3^, David Bloomfield^4^, Charlie Chan^5^, Mark Churn^6^, Susan Cleator^7^, Charlotte Coles^8^, Marie Emson^2^, Andrew Goodman^9^, Clare Griffin^2^, Adrian Harnett^10^, Penny Hopwood^2^, Anna Kirby^11^, Cliona Kirwan^12^, Carolyn Morris^13^, Elinor Sawyer^14^, Navita Somaiah^2^, Isabel Syndikus^15^, Maggie Wilcox^13^, Zotova Rada^16^, Duncan Wheatley^17^, Judith Bliss^2^, John Yarnold^2^

##### ^*1*^*University Hospitals of North Midlands NHS Trust and Keele University*, ^*2*^*The Institute of Cancer Research*, ^*3*^*The Beatson West of Scotland Cancer Centre*, ^*4*^*Brighton and Sussex University Hospitals NHS Trust*, ^*5*^*Nuffield Health Cheltenham Hospital*, ^*6*^*Worcestershire Acute Hospitals NHS Trust*, ^*7*^*Imperial College Healthcare NHS Trust*, ^*8*^*Cambridge University Hospitals NHS Foundation Trust*, ^*9*^*Royal Devon and Exeter NHS Foundation Trust*, ^*10*^*Norfolk and Norwich University Hospitals NHS Foundation Trust*, ^*11*^*The Royal Marsden Hospital NHS Foundation Trust*, ^*12*^*Manchester University NHS Foundation Trust*, ^*13*^*Independent Cancer Patients' Voice*, ^*14*^*Guy's and St Thomas' NHS Foundation Trust*, ^*15*^*Wirral University Teaching Hospital NHS Foundation Trust*, ^*16*^*RTTQA Mount Vernon Hospital*, ^*17*^*Royal Cornwall Hospitals NHS Trust*

**Background:** FAST-Forward aims to identify a 1-week 5-fraction schedule of adjuvant radiotherapy as effective and safe as the UK standard 15-fraction regimen for early breast cancer. We report normal tissue effects (NTEs) up to 3 years (shown in previous breast radiotherapy trials to predict comparisons at 5+ years).

**Method:** The FAST-Forward trial (ISRCTN19906132; NIHR-HTA 09/01/47) randomised patients (pT1-3 pN0-1 M0) following breast conservation surgery or mastectomy (immediate reconstruction permitted) to 40Gy/15 fractions/3 weeks (control), 27Gy or 26Gy/5 fractions/1 week to whole breast or chest wall. A tumour bed boost of 16Gy/8 fractions or 10Gy/5 fractions was given where indicated. NTEs were assessed annually by clinicians, by patients at baseline, 3, 6, 12 and 24 months and from photographs at 2 years compared with a pre-radiotherapy baseline. Schedules were compared using cross-sectional and survival analyses. A lymphatic sub-study is ongoing.

**Results:** From 09/2011-06/2014, 4096 patients were recruited (40Gy: 1361, 27Gy: 1367, 26Gy: 1368). Median follow-up is 48 months (IQR 37-50). Marked NTEs at 2 or 3 years were uncommon (<5% for clinician and photographic assessments, <15% for patient assessments). Clinician assessments of individual NTEs at 3 years and patient assessments at 2 years were similar between schedules. 2-year prevalence of mild/marked change in photographic breast appearance was statistically significantly higher for 27Gy (16.1%, 95%CI 12.9-19.9%) compared with 40Gy (8.3%, 6.0-11.5%), but similar for 26Gy (10.6%, 8.0-13.9%) and 40Gy. 3-year cumulative incidence rates of any clinician-assessed moderate/marked NTE in the breast/chest wall were highest for 27Gy (28.8%, 26.4-31.4%) but similar for 26Gy (21.8%, 19.6-24.2%) and 40Gy (20.8%, 18.6-23.2%).

**Conclusion:** Levels of marked NTEs were low for all schedules. Late effects after 26Gy in 5 fractions over 1 week were similar to 40Gy in 15 fractions over 3 weeks.

Disclosure: Funded by National Institute for Health Research Health Technology Assessment Programme (09/01/47)

Corresponding author: Joanne Haviland

### **116. Adjuvant therapy with nivolumab versus ipilimumab after complete resection of stage III/IV melanoma: Updated results from a phase 3 trial (CheckMate 238)**

#### Jeffrey S. Weber^1^, Mario Mandala^2^, Michele Del Vecchio^3^, Helen Gogas^4^, Ana M. Arance^5^, C. Lance Cowey^6^, Stéphane Dalle^7^, Michael Schenker^8^, Vanna Chiarion-Sileni^9^, Ivan Marquez-Rodas^10^, Jean-Jacques Grob^11^, Marcus Butler^12^, Mark R. Middleton^13^, Michele Maio^14^, Victoria Atkinson^15^, Reinhard Dummer^16^, Veerle de Pril^17^, Anila Qureshi^17^, James Larkin^18^, Paolo A. Ascierto^19^

##### ^*1*^*New York University (NYU) Perlmutter Cancer Center, USA*, ^*2*^*Papa Giovanni XXIII Hospital*, ^*3*^*Medical Oncology, National Cancer Institute*, ^*4*^*University of Athens*, ^*5*^*Hospital Clinic de Barcelona*,, ^*6*^*Texas Oncology–Baylor Charles A. Sammons Cancer Center*, ^*7*^*Hospices Civils de Lyon*, ^*8*^*Oncology Center Sf Nectarie Ltd*., ^*9*^*Oncology Institute of Veneto IRCCS*, ^*10*^*Hospital Gregorio Marañón*,, ^*11*^*Hôpital de la Timone*, ^*12*^*Princess Margaret Cancer Centre*, ^*13*^*Churchill Hospital*, ^*14*^*Center for Immuno-Oncology, University Hospital of Siena*, ^*15*^*Gallipoli Medical Research Foundation and University of Queensland*, ^*16*^*University Hospital of Zurich*, ^*17*^*Bristol-Myers Squibb*, ^*18*^*The Royal Marsden NHS Foundation Trust*, ^*19*^*Istituto Nazionale Tumori Fondazione Pascale*

**Background:** Previously, at 18 months' minimum follow-up, nivolumab demonstrated significantly longer recurrence-free survival (RFS) vs ipilimumab in patients with resected stage III/IV melanoma in the phase 3 CheckMate 238 trial. Here, we report updated 24-month efficacy results.

**Method:** Eligible patients included those ≥15 years of age who underwent complete resection of stage IIIB/C or IV melanoma. 906 patients were randomized 1:1 (stratified by disease stage and 5% PD-L1 expression status) to receive nivolumab 3 mg/kg Q2W (N = 453) or ipilimumab 10 mg/kg Q3W for 4 doses, then Q12W (N = 453) for up to 1 year, or until disease recurrence or unacceptable toxicity. The primary endpoint was RFS with an exploratory endpoint of distant metastasis-free survival (DMFS).

**Results:** At 24 months' follow-up, RFS remained significantly longer for nivolumab vs ipilimumab (hazard ratio 0.66, P<0.0001), with 171/453 vs 221/453 events, respectively. The patient subgroup 24-month RFS rates were higher for nivolumab vs ipilimumab (Table). DMFS remained significantly longer for nivolumab vs ipilimumab; 24-month rates were 70.5% and 63.7%, respectively (hazard ratio 0.76, P = 0.034).

**Conclusion:** With extended follow-up, nivolumab demonstrated sustained efficacy benefit vs ipilimumab in patients with resected stage III/IV melanoma at high risk of recurrence, regardless of disease stage, PD-L1 expression, or BRAF mutation status.

Acknowledgement: These results have been previously presented at the American Society for Clinical Oncology (ASCO) Annual Meeting, June 1-5, 2018, Chicago, IL, USA, and published in the conference proceedings (Abstract 9502) See http://abstracts.asco.org/214/AbstView_214_214567.html for original and CC-BY license.

Disclosure: This study was funded by Bristol-Myers Squibb. Medical writing assistance was provided by StemScientific.

Corresponding author: Jeffrey S. Weber

Table 1 [Abstract 116].

**Table:** 

	Nivolumab	Ipilimumab
RFS, 24-month rates; % (N)		
ITT	62.6% (453)	50.2% (453)
Stage IIIB	70.8% (165)	60.7% (148)
Stage IIIC	58.0% (203)	45.4% (218)
Stage IV	58.0% (82)	44.3% (87)
PD-L1 ≥5%	75.5% (152)	58.4% (154)
PD-L1 <5%	55.2% (275)	45.5% (286)
*BRAF* mutant	61.9% (187)	51.7% (194)
*BRAF* wild-type	63.5% (197)	46.2% (212)

### **117. Hepatic resection following selective internal radiotherapy in the FOXFIRE clinical trial: survival, safety, and histopathology**

#### Pradeep S. Virdee^1^, Helen Winter^2^, Joe Rassam^3^, Rob Goldin^3^, Harpreet S. Wasan^4^, Ricky A. Sharma^5^

##### ^*1*^*Centre for Statistics in Medicine, University of Oxford*, ^*2*^*Green Templeton College, University of Oxford*, ^*3*^*Centre for Pathology, Imperial College*, ^*4*^*Imperial College Healthcare NHS Trust and Imperial College, Hammersmith Hospital*, ^*5*^*University College London Hospitals Biomedical Research Centre, UCL Cancer Institute*

**Background:** Colorectal cancer (CRC) commonly metastasises to the liver and is a leading cause of cancer-related death. The FOXFIRE trial compared the safety and efficacy of radiosensitising chemotherapy (OxMdG: oxaliplatin, 5-fluorouracil and folic acid) with selective internal radiotherapy (SIRT) using yttrium-90 resin microspheres (SIR-Spheres®; Sirtex Medical Limited) to OxMdG alone as first-line management for liver-dominant metastatic CRC. In patients downsized to potentially-curative hepatic resection, we explored survival, safety, and histopathological findings.

**Method:** FOXFIRE (ISRCTN83867919) was an open-label, multicentre, randomised (1:1) trial of 12 cycles of OxMdG with or without SIRT (Wasan HS et al. *Lancet Oncol* 2017). Eligible patients provided written informed consent and were considered untreatable by surgical resection or local ablation. Suitability for surgery was reassessed after 3 months of treatment. Surgical complications were graded using Dindo D et al. *Annals of Surgery* 2004. Cox models and a 5% significance level were used.

**Results:** FOXFIRE randomised 364 patients: 182 per treatment group. Seventy-one (20%) underwent hepatic resections following first-line treatment: 38 (21%) in the OxMdG+SIRT and 33 (18%) in the OxMdG group. Among those resected, 22 in the OxMdG+SIRT and 19 in the OxMdG group had primary tumour in situ. Common surgeries performed were right hepatectomy (n = 22) and segmentectomy (n = 12). Among those resected, overall survival from the time of resection did not differ significantly between groups (OxMdG median = 25.2 months; OxMdG+SIRT median = 21.9 months; HR = 1.55, 95% CI = 0.83-2.89). Twenty patients had grade I/II complications: 11 in the OxMdG and 9 in the OxMdG+SIRT group. Less viable tumour was histologically observed in patients receiving SIRT. Zonal analysis demonstrated that median microsphere density was highest at the tumour periphery and lowest in non-neoplastic tissue.

**Conclusion:** This study reports that hepatic resection following SIRT with OxMdG chemotherapy has an acceptable safety profile and demonstrates the histopathological distribution of microspheres in liver metastases.

Disclosure: Funded by Bobby Moore Fund of Cancer Research UK; Sirtex Medical Limited, Australia

Corresponding author: Pradeep S. Virdee

### **118. A Cancer Research UK phase I/IIa trial of BT1718 (a first in class Bicycle Toxin Conjugate) given intravenously in patients with advanced solid tumours**

#### Udai Banerji^1^, Natalie Cook^2^, T.R. Jeffry Evans^3^, Irene Moreno Candilejo^1^, Patricia Roxburgh^3^, Claire Kelly^2^, Narmatha Sabaratnam^1^, Rashmi Passi^1^, Sawretse Leslie^4^, Sidath Katugampola^4^, Lisa Godfrey^4^, Neil Tremayne^4^, Gavin Halbert^5^, Gavin Bennett^6^, Maria Koehler^6^, Gillian Langford^6^, Marc Pittman^4^, Stefan Symeonides^7^

##### ^*1*^*Institute of Cancer Research & Royal Marsden NHS Foundation Trust*, ^*2*^*University of Manchester & Christie NHS Foundation Trust*, ^*3*^*University of Glasgow & Beatson West of Scotland Cancer Centre*, ^*4*^*Cancer Research UK Centre for Drug Development*, ^*5*^*Cancer Research UK Formulation Unit, University of Strathclyde*, ^*6*^*Bicycle Therapeutics*, ^*7*^*Cancer Research UK Centre for Drug Development & University of Edinburgh*

**Background:** Membrane type I matrix metalloproteinase (MT1-MMP) is a member of the matrix metalloproteinase (MMP) family involved in tissue remodelling through proteolysis of extracellular matrix components. Overexpression of MT1-MMP is seen in multiple tumour types including non-small cell lung cancer (NSCLC), triple negative breast cancer (TNBC) and sarcoma. BT1718 is a novel first in class bicyclic targeting peptide that binds MT1-MMP and is linked to the maytansinoid tubulin inhibitor DM1 by a cleavable disulfide linker. Bicyclic peptides have a low molecular weight in comparison to other conjugated toxin approaches, enabling rapid penetration and a short systemic half-life, potentially reducing toxicity.

**Method:** This is an open label first in human phase I/IIa study. The primary objective is to propose a recommended phase 2 dose (RP2D) and schedule of BT1718. Secondary objectives include pharmacokinetic (PK) parameters and preliminary clinical responses in biomarker pre-defined cohorts of patients. Tertiary objectives include correlative studies related to predictive biomarkers of response.

Dose escalation (phase I)

Stage 1: Exploring an initial twice-a-week schedule. There will be single patient cohorts until either Grade 2 drug related toxicity or dose exceeding 6 mg/m^2^ twice a week. A 3+3 design will then be followed until the RP2D.

Stage 2: Exploring a once-weekly schedule, using a 3+3 design until the RP2D for this schedule has also been established.

Dose expansion (phase IIa)

Part A: 14 patients with MT1-MMP expressing NSCLC or TNBC, treated with BT1718 at the twice-a-week RP2D. At least 6 will have pre- and post-treatment biopsies.

Part B: 14 patients with MT1-MMP expressing NSCLC or TNBC, treated with BT1718 at the once-weekly RP2D. At least 6 will have pre- and post-treatment biopsies.

Part C/D: following parts A & B, a decision will be made to explore the selected schedule in tumour-specific cohort(s) of around 15 patients, with refined MT1-MMP biomarker selection.

Acknowledgement: “© 2018 American Society of Clinical Oncology, Inc. Reused with permission. This abstract was accepted and previously presented at the 2018 ASCO Annual Meeting. All rights reserved see http://abstracts.asco.org/214/AbstView_214_223089.html for original and CC-BY license.

Disclosure: Funded by Cancer Research UK

Corresponding author: Udai Banerji

### **119. Vertebral fractures in patients treated with FOLFIRI-Cetuximab at the Edinburgh Cancer Centre**

#### Amanda Swan, Sally Clive, Lesley Dawson, Donna McGowan, Catriona McLean, Hamish Phillips, Maria Sakala, Mark Zahra, Ewan Brown

##### *Edinburgh Cancer Centre*

**Background:** Osteoporosis in patients with cancer is common and considered multi-factorial in aetiology. A high frequency of symptomatic fractures was observed in patients treated with chemotherapy and cetuximab at our institution. We therefore performed a retrospective analysis of patients with metastatic colorectal cancer treated with cetuximab in combination with chemotherapy to determine the incidence and potential risk factors for the development of fractures.

**Method:** Consecutive patients treated with fluorouracil, folinic acid and irinotecan in combination with cetuximab (FOLFIRI-cetuximab) at the Edinburgh Cancer Centre were retrospectively analysed. Baseline characteristics, number treatment cycles and cumulative steroid dose were collected. FRAX scores were used to calculate 10-year probability of major fracture. Fractures were assessed by reviewing serial CT scan reports during treatment. Patients treated with capecitabine and oxaliplatin (CAPOX) were chosen as a comparison group.

**Results:** 31 patients treated with FOLFIRI-cetuximab and 30 patients treated with CAPOX were reviewed. Patients received a median of 9 two weekly cycles of FOLFIRI-cetuximab (range 1-32) and 6 three weekly cycles of CAPOX (range 1-18). One patient in the FOLFIRI-cetuximab group and no patients in the CAPOX group had a fracture documented prior to treatment. Median FRAX score in the FOLFIRI-cetuximab group was 8%. Ten patients developed new non-metastatic fractures in the FOLFIRI-cetuximab group (28%), 90% were symptomatic. One patient in the CAPOX group (3%) developed a new vertebral fracture. Median cumulative steroid dose for the patients in the FOLFIRI-cetuximab group was dexamethasone 306mg and for the CAPOX group 204mg.

**Conclusion:** Patients with metastatic colorectal cancer treated with FOLFIRI-cetuximab demonstrate a high incidence of symptomatic fractures with significant morbidity. The high incidence may be related to higher steroid exposure although an effect of the chemotherapy regime cannot be excluded. The introduction of bone protection and changes to anti-emetic protocols to reduce cumulative steroid dose should be considered.

Disclosure: None declared

Corresponding author: Amanda Swan

### **120. PERSEPHONE: A randomised phase 3 non-inferiority trial of 6 versus 12 months (m) of adjuvant trastuzumab in patients with HER2 positive (+) early breast cancer (EBC)**

#### Helena Earl^1^, Louise Hiller^2^, Anne-Laure Vallier^1^, Shrushma Loi^3^, Donna Howe^2^, Helen Higgins^2^, Karen McAdam^1^, Luke Hughes-Davies^1^, Adrian Harnett^4^, Mei-Lin Ah-See^5^, Richard Simcock^6^, Daniel Rea^7^, Janine Mansi^8^, Jean Abraham^1^, Carlos Caldas^1^, Claire Hulme^9^, David Miles^5^, Andrew Wardley^10^, David Cameron^11^, Janet Dunn^2^

##### ^*1*^*Cambridge University Hospitals*, ^*2*^*University of Warwick*, ^*3*^*University of Birmingham*, ^*4*^*Norfolk and Norwich University Hospital*, ^*5*^*Mount Vernon Cancer Centre*, ^*6*^*Royal Sussex County Hospital*, ^*7*^*Sandwell and West Birmingham Hospitals NHS Trust*, ^*8*^*Guy's Hospital*, ^*9*^*University of Leeds*, ^*10*^*Christie Research Facility*, ^*11*^*University of Edinburgh*

**Background:** Adjuvant trastuzumab has significantly improved outcomes for HER2+ EBC patients, using the 12m duration empirically adopted from the pivotal registration trials. A shorter duration could reduce toxicities and cost whilst providing similar efficacy. No reduced-duration trial to date has demonstrated non-inferiority.

**Method:** PERSEPHONE is a randomised phase 3 non-inferiority trial comparing 6 to 12m trastuzumab, the largest reduced-duration non-inferiority trial internationally. Mapping onto standard UK practice, all HER2+ EBC patients were eligible. Stratification is by ER status, chemotherapy type, and chemotherapy and trastuzumab timing. The primary endpoint is DFS from diagnosis (first relapse or death). Randomising 4000 patients (1:1) enabled the trial to assess non-inferiority of 6m (5% 1-sided significance, 85% power), defined as ‘no worse than 3%’ below the 12m arm’s assumed 80% 4-year DFS. The pre-planned definitive DFS analysis required 500 events.

**Results:** 4089 patients were randomised from 152 UK sites (Oct’07–Jul’15). ER+ 69%; chemotherapy - 42% anthracycline (A)-based / 48% A and taxane (T)-based / 10% T-based; adjuvant chemotherapy 85%; sequential trastuzumab 53%. At 5.4 years median follow-up, there were 512 (13%) DFS events. 12m and 6m 4-year DFS rates were 89.8% (95%CI 88.3–91.1) and 89.4% (95%CI 87.9–90.7) respectively. The HR of 1.07 (90%CI 0.93–1.24) demonstrated non-inferiority (HR<1.32) of 6m trastuzumab (1-sided p = 0.01). Congruent results were found for overall survival (OS) and within the pre-planned DFS and OS landmark analyses (after 6m of trastuzumab). Heterogeneity was observed in some stratification variables. Cardiac events were reduced in 6m patients (4% v 8% of 12m patients stopping treatment due to cardiotoxicity (p<0.0001)).

**Conclusion:** PERSEPHONE demonstrates 6m of trastuzumab as non-inferior to 12m with an observed difference in DFS of only 0.4% at 4 years. Given cardiac and other toxicities during months 7-12 of treatment, our results support a reduction of trastuzumab duration to 6m.

Acknowledgement: A version of this abstract has been published previously, see http://abstracts.asco.org/214/AbstView_214_217191.html for original and CC-BY license.

Disclosure: Funded by NIHR HTA

Corresponding author: Helena Earl

### **121. Use of Theoretical Domains Framework to identify psychosocial determinants associated with adjuvant hormonal treatment adherence among breast cancer population: mixed method systematic review**

#### Haley Ong, Christine Campbell, David Weller

##### *University of Edinburgh*

**Background:** Adjuvant hormonal therapy (AHT) is a standard treatment for all HR-positive breast cancer patients upon completion of primary systemic therapy. Despite the proven efficacy of AHT in reducing cancer recurrence by 65 %, adherence rates fall to below 50% by the end of the 5year course of therapy. Suboptimal adherence rate not only jeopardies the treatment outcome but also incurs additional healthcare costs. This systematic review aims to summarise the evidence regarding the psychosocial determinants associated with medication-compliance behaviours for adherence and persistence measures among both female and male breast cancer populations using Theoretical Domains Framework.

**Method:** The protocol of this systematic review followed PRISMA-P guideline. We searched MEDLINE (OVID interface), EMBASE (OVID interface), Web of Science, Cochrane Library, Cochrane Central Register of Controlled Trials (CENTRAL, Wiley interface), PsycINFO (OVID interface), PsycARTICLE (OVID interface) and Cumulative Index for Nursing and Allied Health Literature (CINAHL) for studies that examined association between at least one psychosocial domains (cognitive or health beliefs, behavioral or coping, emotional, and social or interpersonal) and at least one medication-taking behavior (adherence, persistence or discontinuation). Papers published from January 1998- April 2018 were included.

**Results:** 1229 titles and abstracts were screened; after title and abstract screening and full-text review, 60 eligible papers were included for narrative synthesis. Negative health beliefs, low perceived efficacy, limited social support are the main psychosocial barriers among female breast cancer patients whereas distress and stigma are commonly cited among male breast cancer patients. Efficient patient-oncologist communication and involvement of patients in decision making greatly decrease patient psychological distress and reduce the misunderstanding on the treatment.

**Conclusion:** AHT medication adherence is an important lever in reducing cancer mortality rates due to recurrence. Identifying the potentially modifiable psychosocial factors will inform comprehensive interventions to improve patient adherence in this ever-growing breast cancer population.

Disclosure: Funded by University of Edinburgh

Corresponding author: Haley Ong

### **122. Tackling early morbidity and mortality in myeloma (TEAMM): assessing the benefit of antibiotic prophylaxis and its effect on healthcare associated infections in 977 patients**

#### Mark Drayson^1^, Stella Bowcock^2^, Tim Planche^3^, Gulnaz Iqbal^4^, Kerry Raynes^4^, Guy Pratt^1^, Kwee Yong^5^, Peter Hawkey^1^, Helen Higgins^4^, Jill Wood^4^, Janet Dunn^4^

##### ^*1*^*University of Birmingham*, ^*2*^*King’s College NHS Trust*, ^*3*^*St George's University Hospitals NHS Trust*, ^*4*^*University of Warwick*, ^*5*^*UCL Cancer Institute*

**Background:** TEAMM was a randomised, double-blind, placebo-controlled multi-centre phase III trial assessing benefits of antibiotic prophylaxis and effects on healthcare associated infections (HCAI). Infection is the biggest cause of early deaths in myeloma. Levofloxacin is effective against the common bacterial infections in myeloma but there is concern about the development of HCAI.

**Method:** Patients were randomised to receive 500 mg levofloxacin or placebo tablets once daily for 12 weeks, dose adjusted for renal function. Patients were eligible if >21 years old with newly diagnosed symptomatic myeloma, intention to treat myeloma actively, and were +/- 14 days into a programme of anti-myeloma treatment. Primary endpoint was the number of febrile episodes (temperature ≥38°C treated with anti-infectives) and/or death by any cause in the first 12 weeks obtained using Kaplan-Meier curves censored at 12 weeks. Febrile episodes, faecal and throat samples were collected at clinic visits every 4 weeks. Patients included in an intention to treat analyses.

**Results:** TEAMM recruited 977 patients from August 2012-April 2016 from 92 centres within the UK. Median age 67 years, 63% male, 76% eGFR>50 ml/min, 54% planned high dose chemotherapy with autologous-stem cell transplantation, 93% ECOG performance status ≤2, 71% with bone disease. Primary endpoint showed a significant benefit for the use of levofloxacin with 95 of 489 patients (19%) on levofloxacin reporting events (87 febrile episodes; 4 deaths; 4 febrile episodes and death) versus 134 of 488 patients (27%) on placebo (112 febrile episodes; 15 deaths; 7 febrile episodes and death); hazard ratio 0.66 (95%CI 0.51-0.86) p=0.002. There was no increased carriage or infection with HCAI on Levofloxacin versus placebo.

**Conclusion:** Prophylactic use of 12 weeks levofloxacin for patients undergoing treatment for active myeloma significantly reduces febrile episodes and deaths without increasing HCAI.

Acknowledgement: A version of this abstract has been published previously, see http://web.oncoletter.ch/id-59th-ash-annual-meeting-and-exposition/oral-sessions/myeloma-therapy-II.html for original and CC-BY license.

Disclosure: Funded by NIHR HTA

Corresponding author: Janet Dunn

### **123. Improving treatment and care for older people with cancer**

#### Rose Gray^1^, Kerry Allen^2^

##### ^*1*^*Cancer Research UK*, ^*2*^*University of Birmingham*

**Background:** 36% of cancer diagnoses in 2015 were in people 75 and over. By 2035, this will rise to 46%. Cancer services in the UK must meet the needs of the patients they serve and adapt to a changing population.

However, cancer survival is generally lower for older patients, older patients are also more likely to be diagnosed in an emergency and are less likely to receive many different treatments.

The Cancer Strategy highlighted that current methods of assessing older patients are not fit for purpose, meaning older people’s needs are not considered.

CRUK commissioned the University of Birmingham to identify solutions for improving the quality of decision-making.

**Method:** A literature review to understand the current evidence base : (1) 15 qualitative interviews with national policymakers, 80 interviews with health professionals. (2) Three UK surveys of primary and secondary care health professionals and older patients. (3)Clinical observations of MDT meetings and multidisciplinary clinics.

**Results:** Older patients have more complex medical and social needs, however the most appropriate support is not always available; including social care, or Clinical Nurse Specialists. Health professionals are likely to include a wide range of clinical factors in treatment decision-making for older patients, but Comprehensive Geriatric Assessments (CGA) are not used consistently in primary or secondary care. Systemic issues also prevent the right information being included in decisions, including with links between primary and secondary care, or links into cancer MDTs. This group will also be hardest hit by wider pressures - a lack of time for consultations, and workforce shortages. Older patients are also typically under-represented in clinical trials, meaning there is limited evidence to support treatment choices.

**Conclusion:** Cancer services are not meeting the needs of older patients and without mitigation, this will worsen. Addressing this is vital if we are to achieve our ambition of world-class cancer outcomes in the UK.

Disclosure: Funded by Cancer Research UK

Corresponding author: Rose Gray

### **124. Results of POUT - A phase III randomised trial of peri-operative chemotherapy versus surveillance in upper tract urothelial cancer (UTUC)**

#### Alison Birtle^1^, Mark Johnson^2^, Roger Kockelbergh^3^, Francis Keeley, Jr.^4^, James Catto^5^, Rik Bryan^6^, Rob Jones^7^, John Chester^8^, David Dolling^9^, Jenny Donovan^10^, Ann French^11^, Chris Harris^12^, Thomas Powles^13^, Rachel Todd^9^, Lucy Tregellas^9^, Caroline Wilson^10^, Andrew Winterbottom^14^, Rebecca Lewis^9^, Emma Hall^9^

##### ^*1*^*Lancashire Teaching Hospitals NHS Foundation Trust*, ^*2*^*Newcastle upon Tyne Hospitals NHS Trust*, ^*3*^*University Hospitals Leicester*, ^*4*^*North Bristol NHS Trust*, ^*5*^*The University of Sheffield*, ^*6*^*University of Birmingham*, ^*7*^*Beatson West of Scotland Cancer Centre*, ^*8*^*Cardiff University/Velindre Cancer Centre*, ^*9*^*The Institute of Cancer Research's Clinical Trials and Statistics Unit*, ^*10*^*University of Bristol*, ^*11*^*Southend University Hospital NHS*, ^*12*^*Consumer Representative*, ^*13*^*St Barts & the London NHS Trust*, ^*14*^*Consumer representative/ Fight Bladder Cancer*

**Background:** The role of chemotherapy post nephro-ureterectomy for UTUC is unclear. POUT addresses whether adjuvant chemotherapy improves disease free survival (DFS) for patients with histologically confirmed pT2-T4 N0-3 M0 UTUC.

**Method:** Patients (up to 345), ≤90 days post NU, were randomised (1:1) to 4 cycles gemcitabine-cisplatin (gemcitabine-carboplatin if GFR 30-49ml/min) or surveillance. Primary endpoint was DFS. The trial was powered to detect a hazard ratio (HR) of 0.65 (improvement in 3yr DFS from 40% to 55%; 2-sided alpha 5%, 80% power) with Peto-Haybittle early stopping rules. Secondary endpoints included toxicity (CTCAE v4) and quality of life (QL; measured using QLQ-C30 and EQ-5D).

**Results:** 261 participants (127 surveillance; 134 chemotherapy) were randomised between 31/05/2012-10/11/2017, at 57 centres. 252 participants joined the QL study. In Oct 2017, independent trial oversight committees recommended POUT recruitment closed as data collected met the early stopping rule for efficacy in favour of chemotherapy. At time of interim analysis (5/09/2017), median follow-up was 17.6mths (IQR 7.5-33.6). Median age 69yrs, 30% pT2, 65% pT3; 91% pN0. Most common grade ≥3 toxicities for chemotherapy pts during treatment were neutropenia 29% (0% surveillance) & thrombocytopenia 7% (0%). 47/123 (surveillance) & 29/125 (chemotherapy) DFS events were reported; unadjusted HR = 0.47 (95%CI: 0.29, 0.74) in favour of chemotherapy (log-rank p = 0.0009). The difference in the mean change from baseline in QLQ-C30 global health status at 3 months was 7.7 (99% CI: 0.9, 14.6; p<0.001) in favour of surveillance and there was no difference between arms at 12 months (6.1 in favour of chemotherapy; 99% CI: -5.3, 17.4; p = 0.08).

**Conclusion:** Adjuvant chemotherapy improved DFS in UTUC without adversely impacting QL. Recruitment to POUT was stopped early due to efficacy favouring chemotherapy; follow-up for OS continues. Results of POUT, the largest UTUC randomised trial, support adjuvant chemotherapy as a new standard of care.

Acknowledgement: A version of this abstract has been published previously, see http://ascopubs.org/doi/abs/10.1200/JCO.2018.36.6_suppl.407 for original and CC-BY license.

Clinical Trial Registry: CRUK/11/027; NCT01993979 – https://clinicaltrials.gov/ct2/show/NCT01993979

Disclosure: Funded by Cancer Research UK (CRUK/11/027)

Corresponding author: Alison Birtle

### **125. PARTNER: Randomised, phase 2/3 trial evaluating safety & efficacy of adding olaparib to platinum-based neoadjuvant chemotherapy in triple negative and/or germline BRCA mutated breast cancer patients**

#### Jean Abraham^1^, Anne-Laure Vallier^2^, Wendi Qian^2^, Andrea Machin^2^, Louise Grybowicz^2^, Stanly Thomas^2^, Melanie Weiss^2^, Caron Harvey^2^, Karen McAdam^2^, Luke Hughes-Davies^2^, Anne Roberts^2^, Rebecca Roylance^3^, Ellen Copson^4^, Karen Pinilla^1^, Anne Armstrong^5^, Elena Provenzano^2^, Marc Tischkowitz^1^, Emma McMurtry^6^, Helena Earl^7^

##### ^*1*^*University of Cambridge, Cambridge, Cambridgeshire*, ^*2*^*Cambridge University Hospitals NHS Foundation Trust, Cambridge, Cambridgeshire*, ^*3*^*University College London, London*, ^*4*^*University of Southampton*, ^*5*^*The Christie NHS Foundation Trust, Manchester*, ^*6*^*AstraZeneca*, ^*7*^*University of Cambridge and NIHR Cambridge Biomedical Research Centre*

**Background:** No specific targeted therapies are available for Triple Negative Breast Cancers (TNBC), an aggressive and diverse subgroup. The basal TNBC sub-group share some phenotypic and molecular similarities with germline BRCA (gBRCA) tumours. In gBRCA patients, and potentially other homologous recombination deficiencies, these already compromised pathways may allow drugs called PARP inhibitors (Olaparib) to work more effectively. Aims: To establish if the addition of olaparib to neoadjuvant platinum based chemotherapy for basal TNBC and/or gBRCA breast cancer is safe and improves efficacy (pathological complete response (pCR)).

**Methods:** Trial design: 3-stage open label randomised phase II/III trial of neoadjuvant paclitaxel and carboplatin +/− olaparib, followed by clinicians' choice of anthracycline regimen. Stage 1 and 2: Randomisation (1:1:1) to either control (3 weekly carboplatin AUC5/weekly paclitaxel 80mg/m^2^ for 4 cycles) or one of two research arms with the same chemotherapy regimen but with two different schedules of olaparib 150mg BD for 12 days. Stage 3: Patients are randomised (1:1) to either control arm or to the research arm selected in stage 2.

**Results:** Stage 1: Safety; Stage 2: Schedule selection using pCR rate and completion rate of olaparib using a “pick-the-winner” design. Stage 3: pCR rate. Enrichment design is applied with an overall significance level 0.05(α) and 80% power. A total of 527 patients will be included to detect an absolute improvement of 15% (all patients) and 20% (gBRCA patients) by adding olaparib to platinum based chemotherapy.

**Conclusion:** PARTNER has been recruiting in UK since 27th May 2016. IDSMC recommended to continue the trial without change after reviewing the Stage 1 safety data. The recruitment of stage 2 was completed in April 2018 and results to be reviewed by the IDSMC in early 2019. The trial is open and enrolling patients to national and international sites.

Acknowledgement: A version of this abstract has been published previously, see http://ascopubs.org/doi/abs/10.1200/JCO.2017.35.15_suppl.TPS591 for original and CC-BY license.

Disclosure: Funded by Cancer Research UK, AstraZeneca

Corresponding author: Jean Abraham

### **126. Propensity Score-Matched Comparison of Focal High Intensity Focused Ultrasound (HIFU) to Laparoscopic Radical Prostatectomy (LRP) for Clinically Significant Localised Prostate Cancer**

#### Daniel Ball^1^, Na Hyun Kim^1^, Ashley McFarlane^1^, Taimur Shah^2^, Max Peters^3^, Enrique Gomez^4^, Saiful Miah^2^, Stephanie Guillaumier^5^, Naveed Afzai^6^, Tim Dudderidge^7^, Feargus Hosking-Jervis^8^, David Eldred-Evans^8^, Richard Hindley^9^, Henry Lewl^10^, Neil McCartan^5^, Caroline Moors^5^, Manit Arya^11^, Raj Nigam^12^, Chris Ogden^13^, Raj Persad^14^, Karishma Shah^5^, Jaspal Virdi^15^, Mark Emberton^5^, Hashim Ahmed^16^, Mathias Winkler^16^

##### ^*1*^*Imperial College Healthcare*, ^*2*^*Imperial Health and Imperial College London (ICL)*, ^*3*^*University Medical Center Utrecht*, ^*4*^*University of Cordoba*, ^*5*^*University College London*, ^*6*^*Dorset County Hospital NHS trust*, ^*7*^*University Hospital Southampton NHS Trust*, ^*8*^*Imperial College London*, ^*9*^*Basingstoke and North Hampshire Hospital*, ^*10*^*Springfield Hospital*, ^*11*^*Princess Alexandra Hospital, Harlow and University College London Hospitals NHS Trust*, ^*12*^*Royal Surrey County Hospital*, ^*13*^*Royal Marsden Hospital*, ^*14*^*Southmead Hospital*, ^*15*^*Princess Alexandra Hospital NHS Strust*, ^*16*^*Imperial health and Imperial College London (ICL)*

**Background:** Focal HIFU is an emerging minimally invasive treatment option for localised prostate cancer. Phase II clinical trials and prospective databases have shown promising oncological outcomes. However, there is a paucity of data comparing HIFU to standard of care therapies such as radical prostatectomy.

**Method:** All consecutive men undergoing either focal HIFU (n = 625) or laparoscopic radical prostatectomy (LRP) (n = 571) between 2007 – 2017 had their pre- and post- operative data collected in prospective databases.

A propensity score was constructed with groups matched 1:1 using nearest neighbour matching based on the propensity score.

Inclusion criteria: PSA <20 ng/ml; Gleason score ≤ 7; T-stage ≤T2c

Exclusion criteria: 3-month nadir of > 0.02 in the LRP cohort; Patients with any missing matching variables [Table 1].

Primary outcome was Failure Free Survival (FFS) defined as transition to salvage or systemic therapy. Up to two focal-HIFUs was part of this intervention as defined in NCRN PART.

**Results:** After inclusion/exclusion criteria, 425 HIFU and 194 LRP patients remained with matching leading to 86 in each cohort. At all-time points, there was no significant difference in FFS (p = 0.29). The actuarial FFS for those undergoing HIFU was 91% at 4-years and 86% for those undergoing LRP Figure 1 and Table 2].

**Conclusion:** Our data show medium-term disease control following focal-HIFU that appears comparable to laparoscopic radical prostatectomy.

Disclosure: Funded by Imperial College Healthcare

Corresponding author: Daniel Ball

### **127. Safety of outpatient management of cancer patients at risk of neutropenic sepsis using MASCC score at the Norfolk and Norwich University Hospital**

#### Ahmed Eltinay^*1*^, Gill Gray^*1*^, Saif Ahmad^*1*^

##### ^*1*^*Norfolk and Norwich University Hospital*

**Background:** Neutropenic sepsis (NS) is a potentially fatal complication of chemotherapy. Patients with febrile neutropenia or neutropenia with other infective symptoms are at risk of NS. The MASCC score (Multinational Association for Supportive Care in Cancer) stratifies potential NS patients as low (≥21) or high-risk (<21) and has NICE approval. Patients at low-risk may not require admission.

**Method:** This study evaluated the use of MASCC score in the management of NS at Norfolk and Norwich University Hospital. Patients with favourable clinical and social circumstances and a low-risk MASCC score were discharged with oral antibiotics. An electronic register was used to study all emergency presentations of possible NS between April 2016 and April 2018 to Acute Oncology (AOS), Medical Admissions (MAU) and A&E.

**Results:** In total, 242 patients presented with potential NS. 170 attended AOS and 72 attended MAU/A&E. The most common primary tumours were breast (64%), lung (13%) and prostate (9.6%). The most common regimens were carboplatin and docetaxel (72%) and FEC (17%). Among AOS attendees 83 patients (49%) were discharged, all of whom had a low-risk MASCC score. Within these 83 patients, median age was 60, and 64 patients (77%) were female. 11 patients (13%) post-discharge were re-admitted within a week; none of these patients died or were admitted for more than one week. Significantly, those patients who were re-admitted had a lower MASCC score at the time of assessment (22 vs 24; p = 0.0028). Among non-AOS attendees, only 2 patients (2.7%) were not admitted suggesting that early discharge using MASCC score had not yet been adopted outside of AOS.

**Conclusion:** MASCC score accurately identifies patients at risk of NS who can be safely managed at home. Re-admission of these patients is more likely if the absolute MASCC score nears 21 and this should inform counseling and follow-up in this patient group.

Disclosure: None declared

Corresponding author: Ahmed Eltinay

### **128. Structure-based virtual screening identifies pranlukast as a CD49f antagonist that reduces stemness in MDA-MB-231 breast cancer cells**

#### Marco Velasco-Velazquez^1^, Inés Velázquez-Quesada^1^, Angel Ruiz-Moreno^1^, Diana Casique-Aguirre^1^, Charmina Aguirre-Alvarado^1^, Fabiola Cortés-Mendoza^1^, Marisol de la Fuente-Granada^1^, Mayra Pérez-Tapia^2^, Aliesha Gonzalez-Arenas^1^, Aldo Segura-Cabrera^3^

##### ^*1*^*National Autonomous University of Mexico*, ^*2*^*National Polytechnic Institute*, ^*3*^*The European Bioinformatics Institute (EMBL-EBI)*

**Background:** Breast cancer is the neoplastic disease with higher mobility and mortality in women worldwide. The Cancer Stem Cell (CSC) model proposes that a subpopulation at the top of the tumor cell hierarchy drives the initiation, maintenance, and therapy response in breast cancer. CD49f is an integrin subunit that is expressed in breast CSCs and promotes maintenance of stemness. Thus, blockade of CD49f is a potential therapeutic approach for targeting breast CSCs.

**Method:** To repurpose drugs as CD49f antagonists, we performed consensus molecular docking using a subdomain critical for heterodimerization and a collection of 13,000+ drugs. Five drugs were selected for in vitro biological validation using MDA-MB-231 cells. We performed limiting-dilution xenotransplantation using the drug with better CSC-selectivity.

**Results:** Pranlukast, a cysteinyl leukotriene receptor 1 (CysLTR) antagonist that is used to treat asthma, inhibited cell adhesion to laminin and decreased the mammosphere-forming efficiency but had no impact on the viability of bulk tumor cells. Short exposure of breast cancer cells to pranlukast reduced CD49f-downstream signaling, including Focal Adhesion Kinase (FAK) and phosphatidylinositol 3-kinase (PI3K) activation. Pranlukast-treated cells showed reduced transactivation of Sox2 promoter and decreased tumorigenicity in vivo, indicating that this drug decreases the number of CSCs.

**Conclusion:** Pranlukast antagonizes CD49f, impairing CSC-associated functions. Since the pharmacokinetics and toxicology of pranlukast are known, it is a potential adjuvant therapy for breast cancer patients.

Disclosure: This work was supported by CONACYT 221103 and PAPIIT IN228616

Corresponding author: Marco Velasco-Velazquez

### **129. A systematic review of eligibility criteria for phase II clinical trials for patients with lung cancer**

#### Gary Doherty^1^, Innocent Ogunmwonyi^2^, Rohan Shotton^1^

##### ^*1*^*Cambridge University Hospitals NHS Foundation Trust*, ^*2*^*University of Cambridge*

**Background:** Significant barriers exist for the successful enrolment of Oncology patients in early phase clinical trials. These include restrictive eligibility criteria that often exclude patients with intracranial disease, abnormal organ function or laboratory tests, and particular co-morbidities. To investigate these further, we performed a systematic review of the eligibility criteria in recent phase II clinical trials for patients with lung cancer.

**Method:** We searched the clinicaltrials.gov database for trials registered in 2016 using the criteria “lung cancer” and “phase 2.” 223 trials were identified. Duplicate/incomplete entries, and those not investigating systemic anti-cancer agents, were excluded. Eligibility criteria were then determined for the remaining 173 trials. Results were matched against known pharmacokinetic/toxicity data for the relevant agents, trial outcomes, sponsorship type, and country. Further analyses were performed to determine temporal trends.

**Results:** Common eligibility criteria were highly variable. Highly significant variability existed for the exclusion of patients with liver function derangement (used in 79.2%; 12.7% even without known hepatic drug metabolism/hepatotoxicity), renal function derangement (used in 79.2%; 14.5% even without known renal drug clearance/nephrotoxicity), low blood cell/haemoglobin counts (used in 78.6%), or coagulation abnormalities (used in 16.8%). Wide ranges of cut-offs and methodologies were used to determine eligibility. While 89.6% of trials permitted patients with brain metastases, 41.2% of these excluded patients taking concomitant corticosteroids. Successfully suppressed HIV/HepB/HepC was permitted in 41.6, 82.7% and 81.5%, respectively. Comprehensive eligibility criteria analyses, pairwise correlations with drug metabolism/toxicities, temporal trends, and correlation between eligibility restrictiveness and trial outcomes, will be presented.

**Conclusion:** The safety of patients participating in clinical trials is paramount. However, our results suggest that the significant variability in eligibility criteria cannot be wholly explained by *a priori* knowledge of the investigational agents’ metabolism and known toxicities, which may lead to irrational and unjustifiable exclusion of patients in a high-need population from access to experimental therapies.

Disclosure: None declared

Corresponding author: Gary Doherty

### **130. The CamGFR model for renal function in patients with cancer: Validation and extension for use with data from isotope mass dilution spectrometry creatinine assays**

#### Edward Williams^1^, Cameron Whitley^1^, Jamie Weaver^2^, Claire Connell^1^, Reed Stratton Geisler^1^, Daniel Giglio^3^, Simon Tavaré^1^, Duncan Jodrell^1^, Tobias Janowitz^1^

##### ^*1*^*Cancer Research UK Cambridge Institute, Cambridge*, ^*2*^*Christie NHS Foundation Trust, Mancherster*, ^*3*^*University of Gothenburg*

**Background:** Estimation of renal function using glomerular filtration rate (GFR) is important for the management of patients with cancer and is often performed using serum creatinine measurements. These are increasingly determined using isotope dilution mass spectrometry (IDMS) assays. We present validation of the CamGFR model that we developed originally with non-IDMS data (JCO, 2017) and a validated extension using IDMS creatinine data.

**Method:** The study was approved by the relevant ethics committees. Data on age, sex, height, weight, serum creatinine, and results for GFR from ^51^Cr-EDTA excretion measurements were obtained from adult patients with cancer from one Swedish and two UK centres. Data were split 4:1 into development and validation datasets. For IDMS data, we refitted the CamGFR model using interaction terms between all creatinine terms and the creatinine measurement type. We assessed bias, accuracy, and precision for GFR using median residuals, root-mean-squared-error (RMSE), and residual interquartile range (IQR). A comparison of carboplatin dosing accuracy based on an absolute percentage error more than 20% (APE > 20%) was undertaken.

**Results:** Data from 6200 patients were obtained, 1913 of these contained IDMS creatinine data. The CamGFR model was the most accurate (RMSE 15.1, 95% CI 14.4 to 15.8) and least biased (median residual 0.85, 95% CI 0.01 to 1.64) model for estimating GFR compared with all other published models for IDMS creatinine data. Body surface area (BSA) adjusted CKD-EPI was the second most accurate model (RMSE 17.0, 95% CI 16.2 to 17.8). Importantly, the CamGFR model reduced the fraction of patients with a carboplatin dose APE >20% to 0.150 (95% CI 0.131 to 0.170) from 0.190 (95% CI, 0.168 to 0.213) for the BSA CKD-EPI.

**Conclusion:** The CamGFR model represents a better standard for estimation of GFR in patients with cancer, in particular when using IDMS creatinine data.

Disclosure: Funded by Cancer Research UK Cambridge Institute

Corresponding author: Cameron Whitley

### **131. Glyoxalase 1 overexpression associated multidrug resistance in cancer chemotherapy**

#### Hafsa Abbas, Dr Mingzhan Xue, Dr Naila Rabbani, Professor Paul Thornalley

##### *University of Warwick*

**Background:** Overexpression of Glo1 induces anti-cancer multi-drug resistance (MDR) in human tumour cells lines and human tumour cells in primary culture. We hypothesize that the cytotoxicity of anticancer drugs is mediated, in part, by inducing increase of MG to cytotoxic levels. This may be achieved by drug-induced increased MG formation and/or decreased MG metabolism; the latter achieved by drug-induced direct or indirect Glo1 inhibition.

**Method:** Using a model human tumour cell line, HEK293 cells were stably vector-transfected to overexpress Glo1 and with empty vector as control. The effect of anticancer drugs on growth and toxicity of HEK293 cells in three conditions (wild-type, Glo1-ioverexpression and empty vector) was studied in vitro and median growth inhibitory concentration (GC50) values determined. The effect of cell permeable Glo1 inhibitor, S-p-bromobenzylglutathione cyclopentyl diester (BBGCp2) on the potency of anticancer drugs was also studied. The glyoxalase system and dicarbonyl metabolism were characterised by measuring cellular activities of Glo1, Glo2, MG reductase and MG dehydrogenase. The flux of formation of D-lactate – a surrogate indicator of flux of MG formation, glucose consumption and net L-Lactate formation were measured in HEK293 cells cultures by end-point enzymatic assays.

**Results:** Doxorubicin, mitomycin C, paclitaxel, mechlorethamine and methotrexate had the highest resistance conferred by Glo1 overexpression: MDR was 16-fold, 15-fold, 8-fold, 7-fold and 7-fold, respectively. BBGCp2 potentiated the cytotoxicity of anti-cancer drugs. There was an increase in flux of formation of D-lactate and L-lactate and consumption of D-glucose in HEK293 cells treated with mechlorethamine, doxorubicin, paclitaxel and methotrexate, compared to untreated cells. However, cells treated with mitomycin C had a decrease and increase in D-lactate and L-lactate formation respectively.

**Conclusion:** Stable increased Glo1 expression in HEK293 cells conferred MDR to clinical anticancer drugs. In most cases, MG was increased by drug-induced increased glycolysis and increased flux of MG formation. Anticancer drugs were not direct Glo1 inhibitors.

Disclosure: None declared

Corresponding author: Hafsa Abbas

### **132. Prehabilitation is feasible during neoadjuvant chemoradiotherapy and may minimize physical deterioration: Results from The REx randomised controlled trial**

#### Susan Moug^1^, Nanette Mutrie^2^, Sarah Barry^3^, Graham Mackay^1^, Robert Steele^4^, Charles Boachie^3^, Annie Anderson^4^

##### ^*1*^*NHS Greater Glasgow and Clyde*, ^*2*^*University of Edinburgh*, ^*3*^*University of Glasgow*, ^*4*^*University of Dundee*

**Background:** Rectal cancer patients undergoing NACRT (neo-adjuvant chemoradiotherapy) experience physical deterioration and reductions in their quality of life. This feasibility study assessed pre-habilitation (a walking intervention) before, during and after NACRT to inform a definitive multi-centred RCT.

**Method:** Patients planned for NACRT then potentially curative surgery were approached (August 2014 - March 2016) (www.isrctn.com; 62859294). Baseline physical and psycho-social measures were performed before NACRT. Participants were randomised to either the intervention (exercise counselling session followed by 13-17 weeks telephone-guided walking programme) or control group (standard care). Follow-up testing was undertaken 1-2 weeks before surgery.

**Results:** Of 296 screened patients, 78 were eligible (26%) and 48 were recruited (62%): 65% male; mean age 65.9 years (range 33.7-82.6). Mean intervention duration was 14 weeks with 75% adherence. 83% of participants completed follow-up testing and both groups recorded reductions in daily walking, however, the reduction was less in the Intervention group. Participants reported high satisfaction and fidelity to trial procedures.

**Conclusion:** This study demonstrates that prehabilitation is feasible in rectal cancer patients undergoing NACRT. Good recruitment, adherence, retention and patient satisfaction rates support the development of a fully powered trial. The effects of the intervention on physical outcomes were promising.

Disclosure: Funded by Chief Scientist Office, Scotland.

Corresponding author: Susan Moug

### **133. Progression free survival as a surrogate endpoint for overall survival in first-line therapy of advanced ovarian cancer: A Gynecologic Cancer InterGroup (GCIG) individual patient-level meta-analysis**

#### Rosalind Glasspool^1^, Liz-Anne Lewsley^2^, Gennaro Daniele^3^, Adrian David Cook^4^, Nozomu Yanaihara^5^, Anna Tinker^6^, Gunnar Kristensen^7^, Petronella Ottevanger^8^, Gerasimos Aravantinos^9^, Ingrid A. Boere^10^, Robert Fruscio^11^, Anna K.L. Reyners^12^, Eric Pujade-Lauraine^13^, Andrea Harkin^2^, Sandro Pignata^14^, Tatsuo Kagimura^15^, Stephen Welch^16^, Eleni, Karamouza^17^, Stan Kaye^18^, Timothy Perren^19^, Susana N Banerjee^20^, Xavier Paoletti^21^

##### ^*1*^*Beatson West of Scotland Cancer Centre*, ^*2*^*Cancer Research UK Clinical Trials Unit, Institute of Cancer Research, University of Glasgow, Glasgow*, ^*3*^*The Fondazione IRCCS - Istituto Nazionale dei Tumori*, ^*4*^*Medical Research Council Clinical Trials Unit at University College London*, ^*5*^*The Jikei University School of Medicine, Tokyo*, ^*6*^*British Columbia Cancer Agency, Vancouver, BC*, ^*7*^*Norwegian Radium Hospital, Oslo*, ^*8*^*Department of Medical Oncology, Radboud University Medical Center, Nijmegen*, ^*9*^*Hellenic Cooperative Oncology Group (HeCOG), Athens*, ^*10*^*Daniel den Hoed Cancer Center, Erasmus University Medical Center, Rotterdam*, ^*11*^*University of Milan & Bicocca San Gerardo Hospital, Monza*, ^*12*^*University Medical Center, University of Groningen*, ^*13*^*Universite Paris-Descartes, Hôpital Armand Trousseau, Paris*, ^*14*^*National Cancer Institute of Naples, Naples*, ^*15*^*Foundation for Biomedical Research and Innovation, Translational Research Informatics Center, Kobe*, ^*16*^*London Regional Cancer Program, London, ON*, ^*17*^*Ligue Nationale Contre le Cancer Meta-Analysis Platform, Department of Biostatistics and Epidemiology, Gustave-Roussy Cancer Campus, Villejuif*, ^*18*^*The Institute of Cancer Research and The Royal Marsden Hospital, Sutton*, ^*19*^*Leeds Teaching Hospitals NHS Trust, Leeds*, ^*20*^*The Royal Marsden NHS Foundation Trust, London*, ^*21*^*Gustave Roussy Institute, Villejuif*

**Background:** Overall survival (OS) is considered the gold standard endpoint for controlled clinical trials but it requires extended follow-up (median OS> 40 months for first line therapy) and large sample sizes. The UK contributed 3 trials to this Gynaecological Cancer Intergroup (GCIG) meta-analysis. The objective was to evaluate whether progression free survival (PFS) based on CA125 measurements confirmed by radiological exam or combined GCIG criteria is a surrogate endpoint for OS in advanced ovarian cancer (AOC).

**Method:** Using the meta-analytic approach on trials published after 2000, correlations between PFS and OS at the individual level, and between treatment effects on PFS and on OS at the trial level, were estimated using Kendall’ tau and copula bivariate (R^2^_Copula_) models respectively. Criteria for PFS surrogacy required R^2^_Copula_ ≥ 0.80.

**Results:** We analyzed individual patient data (IPD) from 10,502 patients in 16 randomized first line trials of standard (n = 7), intensification (n = 5) and maintenance (n = 4) . No heterogeneity in the treatment effects across trials was detected. High correlations were found at the individual level (tau = 0.77) but low correlation at the trial level (R^2^_Copula_ = 0.2). Sub-group analyses led to similar results (see table 1).

**Conclusion:** This large IPD meta-analysis did not establish PFS as a surrogate endpoint for OS in first line treatment of AOC. The analysis was limited by the narrow range of treatment effects observed and/or post study treatment.

Disclosure: Funded by Programme Hospitalier pour la Recherche Clinique, French Ministry of Health

Corresponding author: Rosalind Glasspool

Table 1 [Abstract 133].

**Table:** 

Endpoint / Trial type	Trials N (pts)	tau^a^	R^2^_Copula_^a^	R^2^
Overall	16 (10502)	0.77	0	0.2
CA125 confirmed by radiological exam	10 (5319)	0.75	0.01	0.24
GCIG criteria	5 (4076)	0.78	0.14	0.04
Carbo-Tax as control	10(7336)	0.72	0	0.2
Standard or intensification	12 (7704)	0.77	0.20	0.24
Maintenance	4 (2798)	0.64	0.14	0.01

^a^ tau and R^2^ values range from 0 (no association) to 1 (perfect correlation)

### **134. Precision oncology in surgery: Patient selection biomarkers for operable pancreatic cancer**

#### Stephan Dreyer^1^, Nigel Jamieson^1^, Mark Pinese^2^, Rosie Upstill-Goddard^1^, Colin McKay^1^, Fraser Duthie^1^, Andrew Biankin^1^, David Chang^1^

##### ^*1*^*University of Glasgow*, ^*2*^*Garvan Institute of Medical Research*

**Background:** About 80% of patients with pancreatic cancer (PC) succumb to the disease despite curative resection, many of whom recur within 6 months of surgery. This suggests current staging is inadequate and there is a need to better define the biology and clinical behaviour of PC prior to surgery. This study aimed to develop and validate gene signature sets and biomarkers that accurately predicts disease recurrence patterns in patients undergoing resection for PC.

**Method:** Disease patterns were defined as early recurrence after surgery (< 12 months), liver metastases, lung metastases (no evidence of liver recurrence) and local recurrence only. The molecular features of clinical disease patterns were investigated using transcriptomic analysis and immunohistochemistry (IHC). These were correlated with recurrence patterns, disease presentation (localised, locally advanced and metastatic) and molecular subtypes of PC.

**Results:** Early recurrence, liver metastases and metastatic presentation were strongly associated with gene expression sets that define the squamous subtype of PC *(P* < 0.001). Lung recurrence, localised disease and long-term survival were associated with the classical pancreatic subtype and an anti-tumour immune response *(P* < 0.001). High S100A2 and S100A4 IHC expression was associated with the squamous subtype of PC and were independent poor prognostic factors in 3 independent patient cohorts of PC (totalling *n* = 1184 patients). Using these biomarkers, a molecular prognostic nomogram was generated to identify poor prognostic PC and validated using 3 independent cohorts of PC.

**Conclusion:** Gene signature and biomarker expression sets generated from biological relevant processes and known poor prognostic features of PC can allow accurate prognostication of patients with operable PC. Defining poor prognosis can allow stratification towards systemic neoadjuvant therapy in operable disease. Conversely, if a patient is predicted to have a favourable prognosis, more aggressive and extensive surgery in the setting of borderline resectable or locally advanced disease could be justified.

Disclosure: Funded by Cancer Research UK

Corresponding author: Stephan Dreyer

### **135. Improving patient experience of immunotherapy treatment for melanoma: the Leeds nurse-led immunotherapy telephone clinic**

#### Maria Marples, Helen Jackson, Helen Nicholson, Beverley Ryder, Karen Ingham, Jane Hook

##### *Leeds Cancer Centre*

**Background:** Immunotherapy has transformed the outcomes of metastatic melanoma, but the burden on patients of frequent hospital visits for assessments is high. We devised, implemented, developed and evaluated a nurse-led telephone clinic to assess patients being treated with immunotherapy.

**Method:** We piloted a questionnaire, enquiring about a range of potential immunotherapy toxicities, as well as symptoms of progressive disease and medication changes. This formed the basis of a telephone clinic starting in November 2016, where patients who were established on pembrolizumab immunotherapy were telephoned by a clinical nurse specialist up to 1 week prior to treatment. Outcomes were emailed to a consultant for a prescription, blood tests were done locally, and annotations saved in the electronic patient record. In 2017, patients being treated with nivolumab were added to the clinics. A separate code was created for patients being monitored for immunotherapy toxicity (mainly hepatitis). We evaluated the clinic with a patient satisfaction questionnaire and a detailed review of 6 months' clinical activity.

**Results:** In the first 6 months of the clinic, monthly assessments increased from 14 to 49. Patient satisfaction was high, with 98% patients reporting that the clinic was convenient and saved them time, and 100% that concerns were addressed and they and their GP had the necessary information. Review of the work done in the subsequent 6 months showed that 50% of calls resulted in no additional action, around 40% required telephone advice, and only 4% required medical review that week.

**Conclusion:** Patients on immunotherapy can be safely and effectively assessed on the telephone, which is well-received by patients, and saves transport and clinic costs. This approach is applicable to other tumour types which are treated with immunotherapy, with nurses cross-covering each other to increase service resilience.

Disclosure: None declared

Corresponding author: Maria Marples

### **136. Cost effectiveness analyses of 6 versus 12 months of adjuvant trastuzumab in patients with HER2 positive early breast cancer: Results from the PERSEPHONE trial**

#### Claire Hulme^1^, Peter Hall^2^, Beth Shinkins^3^, Fadi Chehadah^3^, Chris McCabe^4^, Janet Dunn^5^, Louise Hiller^5^, Helena Earl^6^

##### ^*1*^*University of Leeds*, ^*2*^*University of Edinburgh*, ^*3*^*University of Leeds*, ^*4*^*Institute of Health Economics, Alberta*, ^*5*^*University of Warwick*, ^*6*^*Addenbrooke's Hospital, Cambridge*

**Background:** Adjuvant trastuzumab has significantly improved outcomes for HER2 positive early breast cancer patients, using the 12 month duration empirically adopted from the pivotal registration trials. Given an annual per patient cost of trastuzumab treatment of over £30,000 (Euro35,000), a shorter duration has the potential to improve cost-effectiveness if efficacy is maintained.

**Method:** The within trial cost effectiveness analyses uses data collected as part of the PERSEPHONE trial, a randomised phase 3 non-inferiority trial comparing 6 to 12 month trastuzumab, the largest reduced-duration non-inferiority trial internationally. Comprehensive health service activity and costs were collected. Quality of life was measured using the EQ-5D. The analyses adopted the perspective of the health and social care sector over 2 years follow-up. Incremental cost effectiveness ratios (ICER) present the cost per quality adjusted life year (QALY). Additional analysis presents the ICER using the primary outcome of the trial, disease free survival at 4-years. Uncertainty around the ICER is estimated using the non-parametric bootstrap method.

**Results:** 4088 patients were randomised between 4th October 2007 and 31st July 2015. The results of the cost effectiveness analyses will be presented.

**Conclusions:** The cost effectiveness analyses will provide evidence to demonstrate the value for money of 6 versus 12m trastuzumab. The results will inform decisions around reduction of standard trastuzumab duration to 6 month.

Disclosure: Funded by NIHR HTA

Corresponding author: Peter Hall

### **137. Cancer Medicines Outcomes Programme (CMOP): Better use of existing data to understand outcomes in a local population**

#### Jennifer Laskey^1^, Julie Clarke^1^, Kelly Baillie^1^, Yvonne Semple^1^, Olivia Wu^2^, Christine Crearie^1^, Rob Jones^1^, Ashita Waterston^1^, Tanja Mueller^3^, Jiafeng Pan^3^, Marion Bennie^3^

##### ^*1*^*NHS Greater Glasgow and Clyde*, ^*2*^*University of Glasgow*, ^*3*^*University of Strathclyde*

**Background:** Cancer medicines comprise the highest proportion of new medicines introduced within NHS Scotland. There is increasing interest in ‘real world’ data to understand local population outcomes. The Cancer Medicines Outcomes Programme (CMOP) aims to maximise use of routinely collected healthcare data to determine outcomes in the local population.

**Method:** Melanoma and prostate cancer were year 1 exemplar projects. Quality and quantity of data available from electronic record linkage in a safe haven were compared to individual case note review. Outcomes were compared with trial data.

**Results:** Record linkage data appeared broadly comparable to individual case note review (table 1).Outcomes were generally inferior to clinical trial results. Differences in baseline demographics, associated with poorer outcomes, may contribute to this.

**Conclusion:** This demonstrates a more efficient process of describing local population outcomes. Future work includes further validation of datasets in other cancer types.

Disclosure: Funded by Scottish Government

Corresponding author: Julie Clarke

Table 1 [Abstract 137]

**Table:** 

Dataset	Source (electronic record linkage)	Comparable with individual case note review?
Diagnosis		
Diagnosis	SMR06 – initial diagnosis only	✕*
Line of treatment	CEPAS	✓ **
Demography		
Age / Gender	NRS	✓
Scottish Index Multiple Deprivation 2012	CHI	
Performance Status (ECOG PS)	CEPAS	
Baseline blood results	SCI store	
Previous treatments	CEPAS/ARIA^®^/PIS/ SMR00/SMR01	
Co-morbidities	PIS/SMR00/SMR01	✕
Treatment		
Dose information; duration	CEPAS	✓
Toxicity/Reason for discontinuation	CEPAS	✕
Outcome		
Overall survival	NRS	✓***
Biochemical response	SCI store	
Other e.g. time to radiotherapy/opioid prescribing/other treatment	CEPAS/ARIA^®^/PIS/ SMR00/SMR01	

*except metastatic status, **number of previous treatments prescribed, ***except time to radiotherapy

SMR06 = Scottish Cancer Registry; CEPAS= Chemotherapy Prescribing System; NRS = National Records Scotland; CHI = community health index; SCI = Scottish Care Information; PIS = Prescribing Information System; SMR00 & 01 = Scottish Morbidity Records for outpatients & inpatients / day case; ARIA=radiotherapy records

